# Rational Design of Atomically Dispersed Metal Site Electrocatalysts for Oxygen Reduction Reaction

**DOI:** 10.1002/advs.202203391

**Published:** 2023-01-30

**Authors:** Kechuang Wan, Tiankuo Chu, Bing Li, Pingwen Ming, Cunman Zhang

**Affiliations:** ^1^ Clean Energy Automotive Engineering Center and School of Automotive Studies Tongji University 4800 Cao'an Road Shanghai 201804 China

**Keywords:** atomically dispersed metal sites, catalytic mechanism, oxygen reduction reaction, rational design, theoretical calculation

## Abstract

Future renewable energy supply and a cleaner Earth greatly depend on various crucial catalytic reactions for the society. Atomically dispersed metal site electrocatalysts (ADMSEs) have attracted tremendous research interest and are considered as the next‐generation promising oxygen reduction reaction (ORR) electrocatalysts due to the maximum atom utilization efficiency, tailorable catalytic sites, and tunable electronic structures. Despite great efforts have been devoted to the development of ADMSEs, the systematic summary for design principles of high‐efficiency ADMSEs is not sufficiently highlighted for ORR. In this review, the authors first summarize the fundamental ORR mechanisms for ADMSEs, and further discuss the intrinsic catalytic mechanism from the perspective of theoretical calculation. Then, the advanced characterization techniques to identify the active sites and effective synthesis methods to prepare catalysts for ADMSEs are also showcased. Subsequently, a special emphasis is placed on effective strategies for the rational design of the advanced ADMSEs. Finally, the present challenges to be addressed in practical application and future research directions are also proposed to overcome the relevant obstacles for developing high‐efficiency ORR electrocatalysts. This review aims to provide a deeper understanding for catalytic mechanisms and valuable design principles to obtain the advanced ADMSEs for sustainable energy conversion and storage techniques.

## Graphical abstract

In this review, authors systematically summarize effective strategies for the rational design of atomically dispersed metal site electrocatalysts (ADMSEs), and it will provide the readers with a deeper understanding for catalytic mechanisms and valuable design principles for the development of advanced ADMSEs in fuel cells and Zn–air batteries.

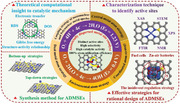

## 1 Introduction

In view of the increasing energy crisis and environmental pollution caused by the consumption of fossil fuels, much attention has been given to the development of renewable and affordable energy sources.^[^ ^1^ ^]^ Recently, sustainable energy conversion and storage techniques have aroused great interests from researchers, and their development mainly relies on a number of crucial electrochemical reactions, such as oxygen reduction reaction (ORR), hydrogen evolution reaction (HER), oxygen evolution reaction (OER), carbon dioxide (CO_2_) reduction reaction, and nitrogen reduction reaction.^[^ ^2^ ^]^ The core of these electrochemical reactions generally involves various electrocatalysts.^[^ ^3^ ^]^ Therefore, the rational design of the electrocatalysts is crucial for the development of advanced energy conversion and storage techniques.

Among various sustainable energy technologies, fuel cells and Zn–air batteries are currently considered as the next‐generation promising energy conversion and storage techniques due to the advantages of absolute zero‐emission and high energy efficiency.^[^ ^4^ ^]^ As the energy conversion devices, Fuel cells have many unique advantages, including long driving range (>500 km), short refueling time (<3 min), low cold starting temperature (about −30 °C), and low operating temperature (<100 °C).^[^ ^5^ ^]^ In addition, Zn–air batteries also show the merits of high theoretical energy density (1086 Wh kg^−1^) and environmental friendliness.^[^ ^2b^ ^]^ Generally, the core of fuel cells and Zn–air batteries mainly involves the ORR processes of electrocatalysts. The oxygen reduction process contains a number of reaction steps regarding the adsorption and desorption of different oxygen‐containing intermediates (such as O^*^, OH^*^, and OOH^*^), and the ideal electrocatalysts require the optimal binding energy at each reaction step. Especially, the binding energy between the electrocatalysts and oxygen‐containing intermediates affects each reaction step, which is closely related to the catalytic activity.^[^ ^6^ ^]^ In fact, the exploration for ORR electrocatalysts can date back to 1970s, and the most attentions have mainly been paid to Pt‐based electrocatalysts.^[^ ^7^ ^]^ At present, Pt‐based electrocatalysts are still the benchmark for ORR due to their higher catalytic activity and more outstanding stability.^[^ ^8^ ^]^ However, there are still many hindrances for the commercial application of fuel cells and Zn–air batteries owing to the high cost, scarcity and sluggish oxygen reduction kinetics of Pt‐based catalysts, especially the cost of Pt usage accounting for 40% of the total fuel cell stack cost and the poor resistance of Pt to poison, such as methanol, carbon monoxide (CO), and chloride ions. Therefore, exploring the low‐cost and efficient ORR electrocatalysts is of vital importance for the development of sustainable society.

In fact, the relevant catalytic reactions usually occur on the surface of the catalysts, and only a few surface atoms from nanoparticles are involved during the catalysis.^[^ ^9^, ^10^ ^]^ The size effect plays a crucial role in the development of advanced ORR electrocatalysts.^[^ ^11^ ^]^ Smaller nanoparticles significantly expose a higher proportion of surface atoms for the catalytic reaction. In particular, reducing the catalyst size to atomic level can effectively increase atomic efficiency, improve the surface selectivity of catalysts, and achieve the maximization of the sensitivity for the support.^[^ ^9^, ^12^ ^]^ Moreover, the well‐defined active site structure with atomic level significantly promotes the exploration of intrinsic ORR mechanism. In fact, Thomas et al.^[^ ^13^ ^]^ first constructed the single atom catalysts in 1995 by dispersing the isolated Ti atoms on the mesoporous Si for the epoxidation of cyclic alkenes. However, the concept of single atom catalysts had not received enough attention for the lack of advanced characterization techniques. As a typical representation of ADMSCs, the concept of “single atom catalyst” was further proposed in 2011.^[^ ^14^ ^]^ Pt_1_/FeO*_x_* was reported for the oxidation of CO by anchoring the isolated Pt sites on the FeO*_x_* nanocrystallites, and the electronic structures of the atomically dispersed Pt atoms were modulated by the electron transfer between the Pt and the FeO*_x_*, which enhanced the catalytic activity and stability of Pt_1_/FeO*_x_*. At present, atomically dispersed metal site electrocatalysts (ADMSEs) have been extensively studied because of many advantages, including the maximum atom efficiency (100%), distinct active sites, and outstanding catalytic performance, which is considered as a potential frontier direction for the development of high‐efficiency electrocatalysts.^[^ ^15^ ^]^ Unlike traditional electrocatalysts such as nanoparticle and nanocluster materials, ADMSEs with atomic scale are generally constructed by dispersing the isolated metal species on the support materials, which possesses a more impressive catalytic performance.^[^ ^11^, ^16^ ^]^ Moreover, ADMSEs as an emerging catalyst are not only a bridge between homogeneous and heterogeneous catalysts with the advantages of high selectivity in homogeneous catalysis and the uniform and controllable atomically dispersed active sites in heterogeneous catalysis, but also considered as a good platform to understand the intrinsic structure–activity relationship at the atomic level.^[^ ^17^ ^]^

Up to now, ADMSEs have been widely reported and demonstrated as efficient electrocatalysts for ORR.^[^ ^2^, ^6^, ^18^ ^]^ Generally, the atomic metal species are dispersed on support, and the interaction between the metal species and support plays a critical role in the regulation of catalytic performance, especially since different supports usually trigger different electronic properties for ADMSEs.^[^ ^6^, ^19^ ^]^ As the important support substrates, carbon‐based substrates with the advantages of good electrical conductivity and large specific surface area are regarded as the first option for constructing ADMSEs due to the strong interaction between the isolated metal species and the supports.^[^ ^20^ ^]^ The strong metal‐support interaction can provide confining and anchoring effects to prevent the agglomeration of metal atoms, improving the stability of electrocatalysts.^[^ ^2^, ^21^ ^]^ Moreover, the interaction is closely related to the electronic structure of isolated metal sites. Rationally designing the metal site and local atomic environments can effectively modulate the density of state (DOS) and the d orbital states to strengthen the metal‐support interaction, optimizing the adsorption free energy of oxygen intermediates on ADMSEs.^[^ ^2^, ^22^ ^]^ In addition, the isolated metal atom directly dispersed on noncarbon‐based substrates is also an effective strategy to promote electron properties of metal species, increasing the ORR activity of the catalysts.^[^ ^23^ ^]^ To date, though some reviews have been published about ADMSEs, most of them only focused on the recent advances in preparation methods and unique catalytic performance of single atom catalysts, especially with little attention being paid to systematically summarizing strategies for the rational design of ADMSEs toward ORR.^[^ ^24^ ^]^ Therefore, developing a timely review to understand the effective design principles is necessary for ADMSEs, but also challenging.

**Figure 1:**
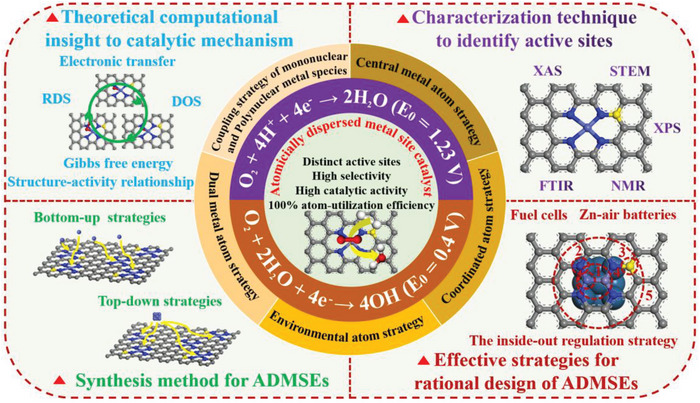
The schematic illustration of the rational design of ADMSEs for ORR summarized in this review.

In this review, the authors aim to provide a valuable guideline for the rational design of the advanced ADMSEs with a special emphasis on regulation strategies of active sites in fuel cells and Zn–air batteries (**Figure** **1**). The review begins with a fundamental catalytic mechanism for ORR, and the theoretical calculations such as density functional theory (DFT) and machine learning (ML) are also discussed to better understand catalytic mechanism for ADMSEs. Then, the advanced characterization techniques for determining the atomic structure and the effective synthesis methods for preparing ADMSEs are also showcased. Subsequently, the authors highly emphasize the strategies for achieving the rational design of the advanced ADMSEs for ORR, in which part of atomic engineering for active sites is particularly discussed to understand the structure–activity relationship for ADMSEs (such as the modulation of central metal atoms, coordinated atoms, environmental atoms and dual metal atoms, and the coupling of mononuclear and polynuclear metal species). Finally, the authors put forward present challenges to be addressed and future research directions for the practical application of ADMSEs in fuel cells and Zn–air batteries.

## 2 Kinetics and Mechanisms for Oxygen Reduction Reaction

ORR is considered as one of the most important electrocatalytic reactions in energy conversion and storage techniques.^[^ ^25^ ^]^ At present, ORR electrocatalysts still face great challenges for the practical application due to the sluggish reaction kinetics.^[^ ^26^ ^]^ Therefore, revealing the intrinsic ORR mechanism is of great importance for the rational design of the advanced electrocatalysts.

### 2.1 Possible Oxygen Reduction Reaction Mechanism for Atomically Dispersed Metal Site Electrocatalysts

In general, the electroreduction of O_2_ mainly includes the adsorption and desorption of oxygen‐containing intermediates, the cleavage and formation of bonds and the transfer of electrons and protons. ORR mechanisms mainly involve two reaction pathways including the four‐electron and two‐electron electrocatalytic pathways, and the product of oxygen reduction greatly depends on the pathways that the reaction follows.^[^ ^27^ ^]^ O_2_ can be directly reduced to H_2_O by a four‐electron electrocatalytic pathway. The multi‐step two‐electron mechanism, however, tends to lead first to the production of hydrogen peroxide, then further to the reduction to H_2_O.^[^ ^28^ ^]^

In fact, the electroreduction of oxygen follows different reaction pathways. The possible electrocatalytic mechanisms for ORR in acidic and alkaline solutions are summarized as follows.

**Figure 2:**
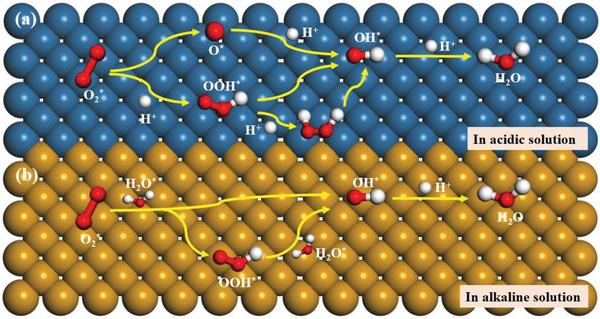
The schematic diagram of the proposed pathways for ORR mechanisms in a) acidic and b) alkaline solutions.

In acidic solution (**Figure** **2**a):

Four‐electron pathway:

(1) $$O_{2}+4H^{+}+4e^{-}\to 2H_{2}O\left(E^{0}=1.23V\right)$$

O_2_ dissociation mechanism:

(2) $$O_{2}^{*}\to O^{*}+O^{*}$$

(3) $$O^{*}+H^{+}+e^{-}\to {\mathrm{OH}}^{*}$$

(4) $${\mathrm{OH}}^{*}+H^{+}+e^{-}\to H_{2}O^{*}$$

O_2_ association mechanism:

(5) $$O_{2}^{*}+H^{+}+e^{-}\to {\mathrm{OOH}}^{*}$$

(6) $${\mathrm{OOH}}^{*}\to O^{*}+{\mathrm{OH}}^{*}$$

(7) $$O^{*}+H^{+}+e^{-}\to {\mathrm{OH}}^{*}$$

(8) $${\mathrm{OH}}^{*}+H^{+}+e^{-}\to H_{2}O^{*}$$

Two‐electron pathway:

(9) $$O_{2}^{*}+2H^{+}+2e^{-}\to 2{\mathrm{HOOH}}^{*}\left(E^{0}=0.67V\right)$$

(10) $${\mathrm{HOOH}}^{*}+2H^{+}+2e^{-}\to 2H_{2}O^{*}\left(E^{0}=1.77V\right)$$

In alkaline solution (Figure 2b):

Four‐electron pathway:

(11) $$O_{2}^{*}+2H_{2}O^{*}+4e^{-}\to 4{\mathrm{OH}}^{*}\left(E^{0}=0.4V\right)$$

Two‐electron pathway:

(12) $$O_{2}^{*}+H_{2}O^{*}+2e^{-}\to {\mathrm{HO}}_{2}^{*}+{\mathrm{OH}}^{*}\left(E^{0}=-0.07V\right)$$

(13) $${\mathrm{HO}}_{2}^{*}+H_{2}O^{*}+2e^{-}\to 3{\mathrm{OH}}^{*}\left(E^{0}=0.87V\right)$$

where * represents a catalytic active site. The ORR can take place in acidic and alkaline solutions via four‐electron and two‐electron electrocatalytic pathways.^[^ ^29^ ^]^ In acidic solution, there are many pathways to achieve electroreduction of O_2_ to H_2_O, including the O_2_ dissociation and O_2_ association mechanisms through a direct four‐electron pathway (Equation (1)). For the O_2_ dissociation mechanism, the adsorbed O_2_ is first dissociated into two O^*^ in catalytic active sites (Equation (2)), then the O^*^ further combines a hydrogen proton and an electron to form OH^*^ (Equation (3)) and H_2_O molecule is thus produced (Equation (4)). As for the O_2_ association mechanism, the O_2_^*^ first combines a hydrogen and an electron into OOH^*^ (Equation (5)), and the OOH^*^ is dissociated in to O^*^ and OH^*^ by cleaving the O—O bond (Equation (6)), then the O^*^ continues to react with a hydrogen and an electron to produce OH^*^ (Equation (7)). Finally, the OH^*^ is reduced to a H_2_O molecule by combing the proton and electron (Equation (8)). For the two‐electron pathway for ORR, the O_2_ electroreduction process shows lower catalytic efficiency than the four‐electron pathway due to the generation of the intermediate HOOH^*^. Unlike the four‐electron pathway, the adsorbed O_2_^*^ consecutively combines two hydrogen protons and two electrons to form the intermediate HOOH^*^ (Equation (9)), then the OH^*^ is produced by cleaving the O—O bond, and the intermediate superoxide is further reduced into a H_2_O molecule by combing a hydrogen and an electron (Equation (10)).^[^ ^30^ ^]^ The possible mechanism for O_2_ electroreduction in alkaline solution is also proposed, and is widely applied to the discharge process of the rechargeable Zn–air batteries.^[^ ^31^ ^]^ In the case of the four‐electron pathway, the O_2_^*^ continuously reacts with a H_2_O molecule and an electron to form the intermediate OH^*^ by a direct four‐electron pathway (Equation (11)). For the two‐electron pathway, the adsorbed O_2_^*^ first reacts with a H_2_O^*^ molecule and an electron to generate the intermediate HO_2_^−^ (Equation (12)), and the O^*^ produced by breaking the O—O bond of the intermediate HO_2_^−^ further combines with a H_2_O^*^ molecule and an electron to form OH^*^ species (Equation (13)).^[^ ^32^ ^]^

(14) $$\Delta G_{\mathrm{OH}}=\Delta G_{\mathrm{OOH}}-3.2\mathrm{eV}$$

(15) $$\Delta G_{\mathrm{OH}}=\frac{1}{2}\Delta G_{O}$$

**Figure 3:**
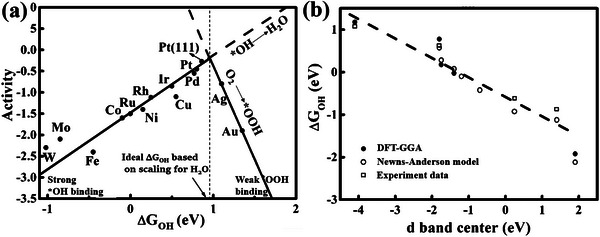
a) The relationship of ORR activity versus Δ*G*_OH_. Reproduced with permission.^[^ ^35^ ^]^ Copyright 2004, American Chemical Society. b) The relationship between d band center and Δ*G*_OH_. Reproduced with permission.^[^ ^39^ ^]^ Copyright 2000, Elsevier.

Based on the description and analysis, the electroreduction of O_2_ into H_2_O includes multiple elementary steps, and involves multi‐electron transfer and reaction intermediates.^[^ ^33^ ^]^ Generally, the oxygen reduction efficiency is closely related to the rate‐determining step (RDS) of catalytic reaction, and the overpotential mainly depends on the change of adsorption free energy (Δ*G*) of the intermediates. In fact, advanced electrocatalysts should have appropriate binding energy for the adsorption of OOH^*^, O^*^, and OH^*^ intermediates, neither too strong nor too weak.^[^ ^34^ ^]^ Taking the four‐electron ORR electrocatalysis of Pt as an example, the four‐electron pathway is considered as the most ideal and efficient O_2_ electroreduction process. The ORR reaction mechanism mainly involves the OOH^*^, O^*^, and OH^*^ intermediates or O^*^ and OH^*^ intermediates, and the binding free energy of OH^*^ intermediate is usually used to evaluate the ORR performance (**Figure** **3**a).^[^ ^35^ ^]^ A linear relationship between the catalytic activity and the binding free energies of OOH^*^, O^*^, and OH^*^ intermediates have been constructed with the aim of determining the optimal binding free energy of OH^*^ intermediate. As a whole, Δ*G*_OH_ = 1/2Δ*G*_O_ (Equation (14)), and Δ*G*_OH_ = Δ*G*_OOH_ – 3.2 eV (Equation (15)).^[^ ^36^ ^]^ When the binding ability is too strong, it is conducive to the adsorption of OOH^*^, O^*^, and OH^*^ intermediates, but inconducive to desorption, which becomes the RDS. The cleavage of the O—O bond and the reduction of O_2_^*^ into O^*^ or OOH^*^ intermediates are blocked when the binding ability is too weak. It will cause the low efficiency of catalytic active sites, and the electrocatalytic activity will be limited, becoming the RDS for ORR.^[^ ^36^, ^37^ ^]^ In addition, the adsorption of O_2_ is crucial to ORR catalytic activity, and the binding ability is closely associated with the oxygen coverage which obviously affects the O_2_ electrocatalytic mechanism.^[^ ^35^, ^38^ ^]^ To understand the effect of electronic structure on the ORR catalytic activity, Jens K. Nørskov et al.^[^ ^39^ ^]^ creatively introduced the d‐band model to study the intrinsic relationship between the electronic structure and the adsorption free energy of intermediates (Figure 3b). The oxygen is first chemisorbed on the catalyst surface, and a strong interaction is produced after the oxygen and d band electron of metal are coupled, which leads to different states of oxygen resonance, affecting the catalytic activity. Therefore, the optimal d‐band electronic structure is crucial for the advanced electrocatalyst.^[^ ^40^ ^]^

For ADMSEs, unlike bulk catalysts, the O_2_ electroreduction process mainly depends on the surrounding coordination environment, and follows the OOH^*^ dissociation mechanism (Equations 5, –8).^[^ ^41^ ^]^ The isolated metal sites play a vital role in the ORR process, and many works have revealed the O_2_ electroreduction mechanism of ADMSEs in depth.^[^ ^42^ ^]^ Ramaswamy et al. provided a fundamental understanding for the O_2_ reduction of the pyrolyzed Fe‐N_4_/C catalysts. It was proved that excessive adsorption energy seriously affected the adsorption efficiency of oxygen, optimizing the binding ability between the metal sites and the adsorbed intermediates by modulating the electronic property of the carbon substrate. The biomimetic active sites ensured the adsorption of O_2_ on Fe‐N_4_, and the solvated O_2_ with the OH^*^ adsorbed on Fe‐N_4_ active sites would limit the activity.^[^ ^42a^ ^]^ Recent studies have also indicated that lots of OH^*^ intermediates are mainly adsorbed on the FeN_4_ center sites from 0.28 to 1.00 V. It is worth noting that OH^*^ intermediates can combine with FeN_4_ to form new active moieties (Fe(OH)N_4_), and regulate the binding strength between the intermediates and the Fe sites.^[^ ^42b^ ^]^ In general, the actual ORR mechanism is closely related to various factors such as the unique metal site structure, the substrate and reaction environment, the adsorption and desorption of the intermediates produced from O_2_ reduction, which significantly affect the electrocatalytic activity.^[^ ^19^, ^25^, ^34^ ^]^ Therefore, the actual ORR mechanism still needs to be further studied.

### 2.2 Theoretical Computational Insight to Catalytic Mechanism for Atomically Dispersed Metal Site Electrocatalysts

At present, ADMSEs have been greatly developed, and more efforts have been devoted to the exploration of intrinsic ORR mechanism for ADMSEs. Until now, many theoretical calculation methods have been developed for unveiling the real catalytic mechanism of ADMSEs, including DFT calculations, ML, ab initio molecular dynamics simulations and microkinetic modeling combined with the experiments,^[^ ^18^, ^43^ ^]^ and especially DFT is widely applied to reveal the reaction mechanism in electrocatalytic field.^[^ ^34^, ^44^ ^]^ DFT was first established by Hohenberg and Kohn in 1964, which was mainly applied to explain the electronic behavior in a system.^[^ ^45^ ^]^ Generally, many valuable results are outputted from DFT, including the electronic density, DOS, magnetic property, energy band structure, and energy change, and these results need to be further analyzed in order to explore the catalytic mechanism. Especially for the catalytic field, both the theoretical calculation and advanced characterization techniques are combined to comprehensively reveal the real ORR catalytic mechanism for ADMSEs, including the adsorption free energy, DOS, electronic structure, and reaction pathway, which are essential for the rational design of the efficient ADMSEs. In addition, the theoretical calculation simulation can also provide the valuable guideline for the design of the experiment to avoid the huge experimental workload.^[^ ^43^, ^46^ ^]^

#### 2.2.1 Determining the Optimal Reaction Path for Atomically Dispersed Metal Site Electrocatalysts

**Figure 4:**
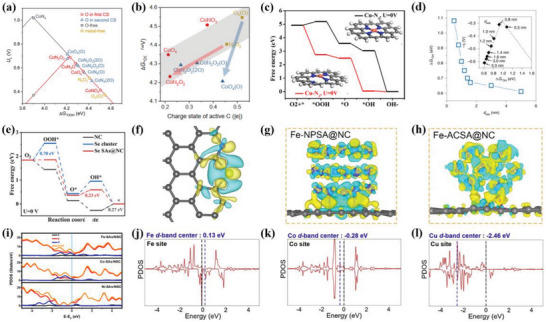
a) ORR activity volcano plots for the 2e^−^ (red color) and 4e^−^ (black) pathway of SACs with different configurations. b) Correlation between the Δ*G*_OOH*_ and charge state of the active site (O‐ adjacent C atom). Reproduced with permission.^[^ ^48^ ^]^ Copyright 2021, American Chemical Society. c) Free energy diagram of Cu‐N_4_ and Cu‐N_3_ sites for ORR. Reproduced with permission.^[^ ^18b^ ^]^ Copyright 2021, American Chemical Society. d) The linear between Δ*G*_OH*_ from the DFT calculation and *d*_site_. Inset: Volcano plot of the ORR overpotentials versus Δ*G*_OH*_. Reproduced with permission.^[^ ^6b^ ^]^ Copyright 2021, Springer Nature. e) Free energy diagram of various electrocatalysts at 0 V. f) Differential charge density for Se SA@NC. Yellow and blue regions represent electron accumulation and depletion, respectively. Reproduced with permission.^[^ ^34a^ ^]^ Copyright 2021, Wiley‐VCH. g) Differential charge density of Fe‐NPSA@NC electrocatalyst. h) Differential charge density of Fe‐ACSA@NC electrocatalyst (the yellow and blue represent the increase and decrease of charge density, respectively). Reproduced with permission.^[^ ^57^ ^]^ Copyright 2022, Wiley‐VCH. i) The computed PDOS of different electrocatalysts with the aligned Fermi level. Reproduced with permission.^[^ ^17b^ ^]^ Copyright 2019, American Chemical Society. The PDOS for j) Fe, k) Co, and l) Cu of FeSA@HNCN*_x_*, CoSA@HNCN*_x_*, and CuSA@HNCN*_x_*, respectively. The blue and black dashed lines represent the d‐band center and the Fermi level, respectively. Reproduced with permission.^[^ ^58^ ^]^ Copyright 2020, Elsevier.

The oxygen reduction efficiency correlates with the RDS of the electrocatalytic reaction, and reducing the energy barrier of the RDS between the active center and the oxygen‐containing intermediate can effectively improve the ORR performance.^[^ ^6^, ^47^ ^]^ Currently, DFT calculation has extensively been applied to investigate the RDS during the electrocatalysis, and the precise identification of the RDS provides a valuable guideline for the rational design of ADMSEs. ORR selectivity of the single atom catalysts could be precisely regulated by controlling the first and second coordination domains, and there was a volcanic plot relationship between the Δ*G*_OOH*_ and thermodynamic limiting potential (*U*_L_) by considering 12 different models (**Figure** **4**a).^[^ ^48^ ^]^ The Δ*G*_OOH*_ controlled the ORR activity, while the kinetic barriers of OOH^*^ protonation and dissociation were closely related to the selectivity. The introduction of O in the first and second coordination domains significantly weakened the adsorption of OOH^*^, and tended to cause a two‐electron pathway for the reaction, where CoN_2_O_2_ and CoO_4_(O) models were located at the peak of volcanoes. Moreover, the functional groups from the second coordination domain effectively regulated the electronic structure and adsorption strength of carbon sites, optimizing the adsorption of OOH^*^ to the volcano peak of two‐electron ORR (Figure 4b). In addition, the functional groups also shifted the OOH^*^ intermediates toward the central metal atoms due to steric hindrance and electrostatic repulsion, further stabilizing the adsorption of the intermediates. The difference of coordinated atom numbers can effectively reduce the energy barrier and optimize the RDS for ORR. The transfer of dynamic behavior occurred from Cu‐N_4_ to Cu‐N_3_, and the Cu‐N_3_ sites were considered to be responsible for ORR.^[^ ^18b^ ^]^ Noteworthily, Cu^+^‐N_3_ sites exhibited a lower Δ*G* in each elementary step than that of Cu^2+^‐N_4_ from the Gibbs free energy diagram. Especially for the first reaction step of the transfer of O_2_ to OOH^*^, there was an energy barrier of 0.28 eV to be overcome for Cu^2+^‐N_4_, while Cu^+^‐N_3_ sites showed an exothermic energy of −2.28 eV, indicating more outstanding catalytic activity for the Cu^+^‐N_3_ sites (Figure 4c). The site distance (*d*_site_) plays a key role in the ORR activity of Fe‐N_4_ monatomic catalysts.^[^ ^6b^ ^]^ The DFT calculation showed that the magnetic moment significantly decreased when the *d*_site_ was less than 1.6 nm, which was demonstrated by the relationship between the calculated Δ*G*_OH*_ and the *d*_site_. The overpotential was closely related to the *d*_site_, and the appropriate binding strength between the Fe site and OH^*^ could be observed in the *d*_site_ of 0.8–1.6 nm, indicating better ORR kinetic (Figure 4d). N and P dual coordination can also optimize the adsorption and desorption of oxygen‐containing intermediates from the central metal sites, enhancing the reaction kinetics of ORR.^[^ ^49^ ^]^ The Cu‐N_2_ with low coordination number was proved to show a lower energy barrier than Cu‐N_4_, indicating a better ORR performance.^[^ ^47^ ^]^ The Δ*G* was also used to reveal the influence of edge‐N on the distortion of central metal sites and the binding mode of oxygen‐containing intermediates with metal sites, and demonstrated that the C—N configuration significantly changed the electronic properties of central metal sites.^[^ ^50^ ^]^ The catalytic efficiency of electrochemical reactions generally depends on the RDS, and lowering the energy barrier of the RDS between the active center and the oxygen‐containing intermediate can further optimize ORR performance.^[^ ^6^, ^25^, ^51^ ^]^ The synergistic effect between the dual metal sites and the intermediates was also investigated by the Δ*G* from the DFT.^[^ ^52^ ^]^ Compared with Co‐N_4_ and Zn‐N_4_ sites, the CoZnN_6_ showed lower energy barriers for catalytic reactions under different potentials (*U* = 0, 0.4, and 0.6 V), especially following the four‐electron path, and showed a smaller dissociation barrier (0.15 eV) for the conversion of O_2_H^*^ into O^*^ and OH^*^ at 0.4 V, while there were higher barriers of 0.15 and 0.60 eV for the transformation of H_2_O_2_^*^ to O^*^ and H_2_O in the Co‐N_4_ and Zn‐N_4_ sites respectively, indicating the best ORR performance for the CoZnN_6_. Similarly, at low potential of 0 V, the free energy pathways for NC, Ni—N, and Co—Ni—N were exothermic and could proceed spontaneously, and the limiting potential followed the trend of NC (0.14 V) < Ni—N (0.6 V) < Co—Ni—N (0.88 V).^[^ ^53^ ^]^ However, the Co—Ni—N showed a lower limiting barrier of 0.35 eV for RDS of the protonation of OH^*^ than that of NC (1.09 eV) < Ni—N (0.63 eV) with RDS of hydrogenation of O_2_ under potential of 1.23 V, indicating the theoretical evidence for the better bifunctional property of the Co‐Ni‐N catalysts. In addition, Fe—Mn,^[^ ^2b^ ^]^ Fe—Ni,^[^ ^54^ ^]^ Pt—Fe,^[^ ^55^ ^]^ and Co—‐Co^[^ ^56^ ^]^ dual site configurations are also explored by DFT.

#### 2.2.2 Revealing Electronic Interaction for Atomically Dispersed Metal Site Electrocatalysts

The catalytic performance generally depends on the electronic structure, since the electronic interaction for ADMSEs effectively triggers the charge redistribution, modulates the valence state and optimizes the binding strength between the active sites and the reaction intermediates during the electrocatalysis, enhancing the catalytic performance of ADMSEs.^[^ ^2^, ^26^ ^]^ The atomically dispersed Se sites had the RDS from the hydrogenation protonation of O^*^ into OH^*^, and exhibited an energy barrier of 0.23 eV, which was lower than that of the Se clusters (0.7 eV) (Figure 4e).^[^ ^34a^ ^]^ Noteworthily, according to the differential charge density diagram, the monodispersed Se sites could trigger stronger electronic interaction in Se single atom catalysts than Se clusters, which effectively regulated the adsorption and hybridization of reactants (Figure 4f). Fe clusters effectively induced electron redistribution and facilitated OH^*^ desorption from the isolated Fe metal centers.^[^ ^59^ ^]^ Remarkably, the electron transfer around the Fe centers to the adjacent C significantly increased the positive charges of the metal centers, and weakened the adsorption of OH^*^ (Figure 4g,h). Compared with Fe nanoparticles coupling single metal sites (0.64 and −3.6 eV) and isolated Fe sites (0.75 and −3.13 eV), Fe clusters functionalizing isolated metal sites on a N‐doped carbon substrate had the highest Bader charge (0.79) and the weakest OH^*^ adsorption energy (−2.79 eV), which was favorable for OH^*^ desorption. Moreover, the relationship between the half‐wave potential (*E*_1/2_) and bond length further proved the result mentioned above. The electronic transfer between the central metal sites and support substrates generally appeared due to the different electronegativity.^[^ ^60^ ^]^ The electronic transfer from the Co sites of Co‐N_4‐_ *_x_* C*_x_* to carbon support could be found by the differential charge density diagram.^[^ ^60^ ^]^ The heteroatoms introduced in the first or second coordination shell generally acted as the electron donor, and regulated the electronic distribution.^[^ ^2^, ^22^, ^26^, ^61^ ^]^ From the electron structure of the N_2_‐Zn‐B_2_, the Zn had a lower Bader charge of 0.75 than other catalysts, while the Bader charge of B reached 3, indicating that the introduction of B atom could give the N atom more electrons than the Zn, and make the Zn 4s hold more electrons.^[^ ^26b^ ^]^ The Fe sites with S and N coordination were constructed for efficient ORR catalysis, and the S was proved as a donor to donate electrons to the central Fe site from the differential charge density diagram, regulating the electronic structure of Fe site and weakening adsorption of OH^*^.^[^ ^22^ ^]^ The introduction of Cl could also regulate the charge of central Fe sites, optimizing the bonding strength of OH^*^ on active sites.^[^ ^61^ ^]^ The S on the second coordination shell could also induce the charge redistribution around the central Ru sites.^[^ ^2a^ ^]^ S acted as an electron donor, and electrons were generally transferred from the S to the Ru site with N as the bridge, leading to a huge difference on charge density. The effect of support substrate on the electronic structure of ADMSEs was also explored.^[^ ^6a^ ^]^ The substrate strain from CuN_2_C_2_ catalysts could trigger more significant electronic interaction between the Cu sites and the adsorbed intermediates.^[^ ^6a^ ^]^ Unlike the conventional model, more electrons were transferred from the central Cu sites to the adsorbed O_2_^*^ due to the geometric distortion of substrate. Notably, the Cu single site catalysts on carbon nanotube with the diameter of 8 nm (Cu/CNT‐8) showed lager electronic transfer of −0.041 than Cu/CNT‐4 (−0.023) and Cu/G with graphene as support (−0.037), further facilitating reduction of adsorbed species. Similar result can be found from Co nanoparticle‐Co‐N_4_ composite sites (Co NP‐CoN_4_).^[^ ^62^ ^]^ The researchers found that the synergistic effect between Co NP‐CoN_4_ composite sites and deformed CoN_4_ could effectively enhance the oxygen reaction kinetic, and the nano‐geometric deformation of substrate significantly regulated the electronic transfer between the active sites and the adsorbed oxygen‐containing intermediates than the plane‐symmetric Co‐N_4_. The edge site could induce more beneficial electronic effect for ORR.^[^ ^63^ ^]^ The FeN_4_ edge sites were more active for catalytic reactions (ORR and OER) than the in‐plane sites. The edge sites resulted in the transfer of more electrons from the central Fe sites to the surrounding N due to the reduced N coordination number. Moreover, the Fe‐N_4_ edge sites showed lager Bader charges of 1.17 than the in‐plane sites (0.97), further demonstrating the result above. Also, the introduction of defect further triggered the asymmetric redistribution of charges around the central metal sites, facilitating the synergistic effect between the defect and the coordinated atom.^[^ ^64^ ^]^ Different types of N coordination also cause different charge distribution, and the pyrrolic N generally exhibits weaker electron depletion than the pyridinic N, indicating more positive charge for the pyrrole‐type Fe‐N_4_.^[^ ^44^ ^]^ The atomically dispersed dual metal sites also lead to the charge redistribution, achieving the optimization of electronic structure.^[^ ^65^ ^]^

#### 2.2.3 Exploring Binding Strength between Active Sites and Intermediates

DOS generally depends on the number of states allowed to be occupied by electrons in a particular energy level state. The binding strength, d‐band center and the antibonding orbitals of ADMSEs can be revealed by DOS, which is of vital importance for the understanding of the catalytic mechanism. For example, Zhang et al.^[^ ^17b^ ^]^ conducted the projected DOS (PDOS) to investigate the effect of the local coordination environment of metal sites anchored on porous N,S‐codoped carbon (M‐SAs/NSC) (M = Fe, Co, and Ni) for ORR performance. Noteworthily, compared with Co‐SAs/NSC and Ni‐SAs/NSC catalysts, the Fe‐SAs/NSC exhibited the highest density states near the Fermi level with a higher charge density of 0.11 than Co of CoN_3_S_1_ (0.09) and Ni of NiN_3_S_1_ (0.03), further demonstrating the highest conductivity and electronic transfer for the Fe‐SAs/NSC (Figure 4i). Similarly, PDOS was used to reveal the catalytic mechanism of the Cu—N single atom catalysts for the Zn–air batteries.^[^ ^58^ ^]^ Compared with the Fe single atoms on hollow nano‐spheroids of N‐deficient carbon nitride frameworks (FeSA@HNCN*_x_*) (0.13 eV) and CoSA@HNCN*_x_* (−0.28 eV), CuSA@HNCN*_x_* showed a lowest d‐band center of −2.46 eV, and similar to the Pt (−2.67 eV). It was remarkable that the partially filled anti‐bonding state could endow the CuSA@HNCN*_x_* with an appropriate binding energy with reaction intermediates, while the empty anti‐bonding states for the FeSA@HNCN*_x_* and CoSA@HNCN*_x_* generally caused stronger binding strength, which were inconducive to desorption of intermediates. The isolated Fe and Co sites coordinated with the pyrrolic N also showed higher DOS near the Fermi level than Ni single atom catalysts (Figure 4j–l).^[^ ^66^ ^]^ The DOS was also applied to evaluate the effect of local coordination environment on binding strength and the antibonding orbitals of ADMSEs. Compared with the conventional In‐N_4_, the lowest unoccupied molecular orbitals (LUMO) of In‐N_3_S and In‐N_4_B were located at the lower energy of 1.53 eV and the higher energy of 2.31 eV, respectively.^[^ ^34b^ ^]^ It was worth noting that the In‐N_3_SB showed a middle LUMO, indicating the optimization of the binding strength for In‐N_3_SB due to the introduction of B and S heteroatoms. From the PDOS diagram, the d‐band center of Co from the CoN_4_ was located at 0.86 eV, while the dual coordination of P and S significantly decreased the d‐band center to −1.98 eV for the CoN_3_PS catalysts on hollow carbon, and in particular, S had more impact on the electronic structure.^[^ ^67^ ^]^ Similarly, Qin et al.^[^ ^2a^ ^]^ also proved the introduction of S could lower the d‐band center of Ru atoms by the DOS diagram, optimizing the binding strength between the Ru sites and the adsorbed intermediates. Changing N coordination numbers also affected the binding strength between the active sites and the intermediates. The FeN*_x_* with low N coordination generally showed higher hybrid strength, leading to the strong binding between the metal sites and the intermediates. The weak hybridization between the O 2p and the Fe 3d appeared for the FeN_5_, and the FeN_4_ catalyst had the medium‐strength Fe—OH bond of 0.77 eV, which was similar to the Pt (111) surface, indicating the best ORR activity for the FeN_4_ catalysts.^[^ ^68^ ^]^ Different types of N generally endowed ADMSEs with different ORR activities, such as pyridinic N, graphitic N and pyrrolic N. The synergistic effect between the Co‐N_4_ and the coordinated N could effectively optimize the d‐band center, and the isolated Co site coordinated with the graphitic N showed the lowest d‐band center of −1.91 eV compared with the conventional Co‐N_4_ site (−1.699 eV).^[^ ^28b^ ^]^ DOS is also used to prove the hybridization between the isolated metal sites and the oxygen‐containing intermediates.^[^ ^16^, ^69^ ^]^ Compared with Cu‐N_4_ (−1.060 eV) and Zn‐N_4_ (1.552 eV), the dual metal sites Cu‐N_4_/Zn‐N_4_ showed a significant negative shift for energy, lowering to −1.733 eV, which indicated that the synergistic effect between the dual metal sites effectively regulated the electronic structure and optimized the binding strength between the active sites and intermediates.^[^ ^70^ ^]^

Apart from the DFT method, other theoretical calculation simulations have also been applied to reveal the catalytic mechanism and predict the potential catalytic materials, further accelerating the exploration and design of new materials.^[^ ^43^, ^71^ ^]^ Although it's difficult for current experiments to fully achieve the prediction of the theoretical calculation, the process of exploration can provide new candidates and ideas to construct the advanced ADMSEs for ORR. Moreover, to achieve batch production and commercialization of the lab‐scale synthetic material, the process can narrow down the study scope and screen the optimal electrocatalysts to avoid many unnecessary experimental exploration steps.^[^ ^43^, ^71^ ^]^

**Figure 5:**
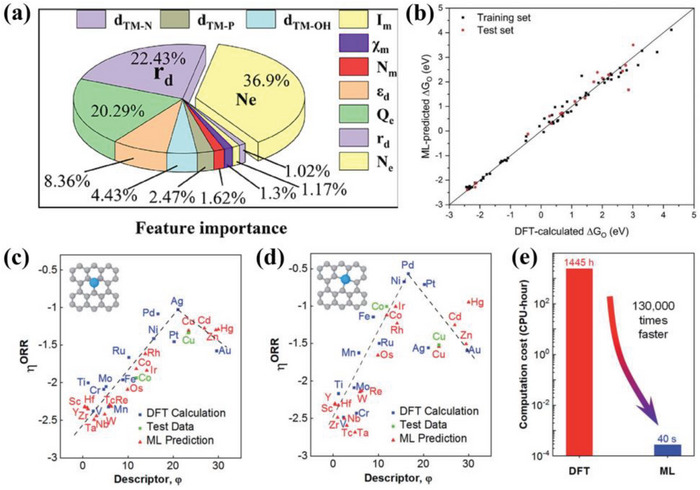
a) Feature importance of each descriptor with the corresponding proportion. Reproduced with permission.^[^ ^72^ ^]^ Copyright 2022, American Chemical Society. b) Comparison of DFT‐calculated and ML‐predicted Δ*G*_O_. Black and red points represent the data of the training set and test set, respectively. Reproduced with permission.^[^ ^43b^ ^]^ Copyright 2021, The Royal Society of Chemistry. The volcano plot relationship of ORR activity versus descriptor 4 of SACs on c) a SV‐site and d) a DV‐site, respectively. e) Comparison of the computation time for predicting the ORR performance from DFT calculations and ML prediction. Reproduced with permission.^[^ ^43a^ ^]^ Copyright 2020, American Chemical Society.

The defective AlP (aluminum phosphide) system based on single transition metals (TM) had been proposed to verify bifunctional oxygen electrocatalysis (ORR/OER) by both the DFT and ML methods.^[^ ^72^ ^]^ The result showed that the catalytic activity was improved by replacing two P atoms with two N atoms in the Al vacancy of TM‐anchored AlP monolayer. Based on DFT and ML result of a series of single atom catalysts, the Co atoms anchored on the substrate of two N atoms replacing two P atoms with an Al vacancy (Co@VAl‐2NP‐AlP) and Ni@VAl‐2NP‐AlP systems had the best OER (0.38, 0.24 V) and ORR overpotential (0.25, 0.39 V), respectively. In addition, Fe@VAl‐2NP‐AlP had an ORR overpotential of 0.48 V, which was also an ideal electrocatalyst for ORR. Moreover, ML method based on gradient lifting regression model also proved that d electron number, atom radius and charge transfer of TM atom were also the main descriptors related to adsorption behavior (**Figure** **5**a). Both DFT and ML were also applied to reveal the activity origin of the bifunctional oxygen single atom catalyst on the C_2_N.^[^ ^43b^ ^]^ The researchers tested 27 single atom catalyst models, and the Au and Pd@C_2_N with an overpotential of 0.38 and 0.40 V were proved to be the potential ORR electrocatalysts. Remarkably, there was a linearity between the Δ*G*_O_ from DFT and ML, indicating the ML could achieve the precise prediction without DFT calculation (Figure 5b). Similarly, the DFT‐ML hybridization also demonstrated the potential catalytic activity of RhPc, Co‐N‐C, and Rh‐C_4_N_3_ with lower overpotential than other precious metals, and the electron number of the d orbital was the crucial descriptor for the single atom catalysts on N‐doped carbon for ORR/OER. This work provides the universal guideline for the rational design of the desired ORR electrocatalysts.^[^ ^46^ ^]^ Also, the TM atom dispersed on the defective g‐C_3_N_4_ with N vacancy (TM/VN‐CN) was proved to be the potential electrocatalyst, and the Rh/VN‐CN showed the optimal catalytic activity for ORR/OER. Moreover, the volcano plots and contour maps were built to reveal the activity origin by both DFT and ML, and the enhanced ORR/OER activity had a close relationship with the d‐band center and the electron number of d‐orbital for the TM.^[^ ^73^ ^]^ The theoretical calculation simulation was also applied to explore the thermodynamic stability of ADMSEs. Wu et al.^[^ ^43a^ ^]^ demonstrated that the decomposition energy barrier mainly dominated the stable dispersity of the isolated metal atoms on the carbon support, and the intrinsic overpotential descriptor could better connect the ORR activity with the single atom catalysts. The prediction resulting from the DFT and ML calculations exhibited a good volcano relationship for the single atom catalysts on carbon support with vacancy and no defect, indicating that the ML calculation efficiently promoted the understanding of the intrinsic catalytic mechanism for ORR (Figure 5c,d). In addition, the ML calculation showed higher computational efficiency than the DFT (Figure 5e). Similarly, different models were established to predict the aggregation energy between the isolated Cu site and O^*^ by both DFT and ML calculations.^[^ ^74^ ^]^ The theoretical simulation was used to screen the most potential catalyst from 104 graphene‐supported single atom catalysts models, and achieved the direct prediction for the limiting potentials of ORR/OER/HER.^[^ ^71^ ^]^ Besides, DFT and ML hybridization are also applied to explore the ORR catalytic mechanism for the dual site catalysts. Deng et al.^[^ ^75^ ^]^ established 26 homonuclear and 55 heteronuclear dual site catalyst models and obtained the activity volcano relationship by both DFT and ML. The result showed that the heteronuclear dual site catalysts had more promising ORR activity than the homonuclear catalysts. It was worth noting that only one descriptor was unable to accurately reflect catalytic activity, and the activity origin was mainly dominated by many simple geometric parameters. Zhu et al.^[^ ^76^ ^]^ also proved that the activity origin for ORR was mainly ascribed to the electron affinity, electronegativity, and radii of the metal atoms, and established the reliable predictor equations to precisely describe the ORR activity of dual site catalysts.

## 3 Characterization Techniques to Identity Active Sites for Atomically Dispersed Metal Site Electrocatalysts

Nowadays, ADMSEs have been widely applied for ORR, providing a comprehensive understanding that ADMSEs are significant and indispensable for the development of advanced electrocatalysts.^[^ ^25^, ^34^ ^]^ Due to the limited atomic resolution, common techniques cannot provide more detailed atomic structure information for ADMSEs. Many advanced characterization techniques at atomic level have rapidly developed and are used to elucidate the intrinsic catalytic mechanism and understand the structure–activity relationship.^[^ ^2^, ^25^ ^]^ Atomic resolution characterization techniques are commonly used to directly observe the location of the isolated metal sites and identify the coordination environment, binding mode, oxidation states of isolated metal sites, and provide the detailed structural information for the construction of the theoretical model.^[^ ^6^, ^29^, ^63^, ^77^ ^]^ In addition, characterization techniques are also applied to investigate the synergistic effect between the metal atom and support for ADMSEs.^[^ ^6^, ^34^ ^]^ The development of advanced characterization techniques with atomic level for the catalyst materials can better identify the real catalytic site and understand the reaction mechanism of ADMSEs.^[^ ^18^, ^25^ ^]^ The following subsection will introduce many crucial characterization techniques in detail such as X‐ray absorption spectroscopy (XAS), aberration‐corrected scanning transmission electron microscopy (AC‐STEM), and Mössbauer spectroscopy, and briefly discuss some traditional characterization techniques including nuclear magnetic resonance spectroscopy (NMR), X‐ray photoelectron spectroscopy (XPS), and Fourier‐transform infrared spectroscopy (FTIR).

### 3.1 X‐Ray Absorption Spectroscopy

The synchrotron‐based XAS is a powerful characterization technique, and extensively applied to reveal the chemical state and local coordination information of isolated metal atoms.^[^ ^6^, ^78^ ^]^ The operation of XAS requires the synchrotron radiation due to the high energy. When the material is scanned by the incident X‐ray with a specific energy, the core electrons are excited into the unoccupied orbitals with an energy range of 5–150 eV. And the excited electrons are scattered by the surrounding atoms, which will cause the enhanced absorption at the edge and oscillatory structures in a common XAS spectrum. In general, XAS can be divided into X‐ray absorption near edge structure spectroscopy (XANES) and the complementary extended X‐ray absorption fine structure spectroscopy (EXAFS) according to the relative energy to the absorption edge of the specific element.^[^ ^79^ ^]^ Recent years have witnessed the wide utilization of XAS characterization technique via the combination of XANES and EXAFS to reveal the atomic and electronic structure of atomically dispersed metal species and determine the model for theoretical calculation, providing a better understanding for the catalytic mechanism of ADMSEs.^[^ ^6^, ^41^, ^80^ ^]^ The XANES is usually applied to reveal the electronic structure of the probed atoms. However, the EXAFS is sensitive to the coordination environment, and the detailed geometry information of the coordinated atoms around the metal center can be obtained, such as the coordinated atom type, coordinated atom number, and bond length.

#### 3.1.1 X‐Ray Absorption near Edge Structure Spectroscopy

**Figure 6:**
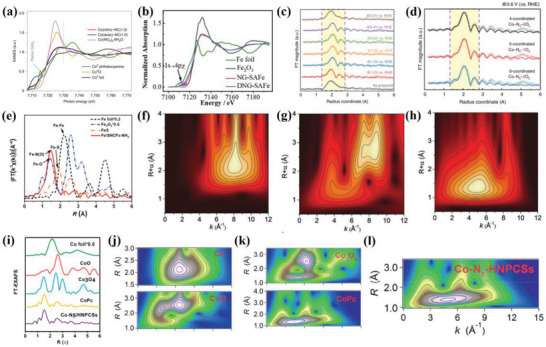
a) Co K‐edge XANES spectrum for different samples. Reproduced with permission.^[^ ^3b^ ^]^ Copyright 2020, Springer Nature. b) K‐edge XANES spectrum of Fe, FT‐EXAFS spectrum of NG‐SAFe, DNG‐SAFe, and the reference samples. Reproduced with permission.^[^ ^83^ ^]^ Copyright 2020, Wiley‐VCH. c) The phase‐corrected FT‐EXAFS (k^3^‐weighted) obtained under different operando biases. d) The fitted EXAFS spectrum at a bias of 0.6 V (vs RHE) for the as‐prepared samples. The fitting region was labeled with soybean color. Reproduced with permission.^[^ ^84^ ^]^ Copyright 2020, Springer Nature. e) Fe K‐edge k^3^‐weighted FT‐EXAFS spectrum of different catalysts. Reproduced with permission.^[^ ^85^ ^]^ Copyright 2021, Wiley‐VCH. The Fe K‐edge k^3^‐weighted WT‐EXAFS spectrums of f) Fe foil, g) Fe_2_O_3_, and h) Fe/N‐G‐SAC. Reproduced with permission.^[^ ^63^ ^]^ Copyright 2020, Wiley‐VCH. i) The FT‐EXAFS spectrum at R space for Co‐N_5_ catalysts. j–l) WT‐EXAFS spectrums of Co‐N_5_ catalysts. Reproduced with permission.^[^ ^86^ ^]^ Copyright 2018, American Chemical Society.

XANES can provide more detailed information about the electronic structures and spatial arrangements of coordinated atoms and oxidation states as well as coordination chemistries of the probed atoms by using standard samples as a comparison.^[^ ^2^, ^15^ ^]^ In the process of analysis, many significant main peaks in XANES include more information for investigating the unoccupied orbits of the probed atoms, and are generally referred to as the white line (WL). The variations in intensity and shape of WL are usually used to probe the chemical states and reveal the electronic interaction of ADMSEs.^[^ ^6^, ^34^ ^]^ For example, Xie et al.^[^ ^3b^ ^]^ obtained the Co K‐edge XANES spectrums of Co single atom catalysts with Co(mIm)_4_ (Co(mIm)‐NC(1.0)), Co(acac_)3_ (Co(acac)‐NC(1.0)), and other Co^II^ reference samples and revealed the valence states of Co atoms (**Figure** **6**a). The result showed the Co(mIm)‐NC(1.0) had similar Co K‐edge XANES spectrums with other references (Co^II^), indicating the same oxidation state of +2 for Co in Co(mIm)‐NC(1.0). The higher pre‐edge peak intensity for Co(mIm)‐NC(1.0) at the pre‐edge region could be found compared with octahedral Co^II^(NO_3_)_2_·6H_2_O and square planar Co^II^ phthalocyanine, suggesting less centrosymmetric coordination environment for Co(mIm)‐NC(1.0). Similarly, Wu et al.^[^ ^81^ ^]^ also proved that the valence state of Co atom from Co‐N_4_/C was between 0 and +2 with the XANES spectrums of Co foil, CoO, and Co_3_O_4_ as references. In addition, the XANES spectrum is also applied to investigate the oxidation state of Fe species in Fe SACs.^[^ ^82^ ^]^ The Fe_2_O_3_, Fe foil and iron phthalocyanine (FePc) were used as the references, and the Fe SACs showed the absorption edge was highly closed to the Fe_2_O_3_ from the XANES spectrum, indicating the oxidation state of +3 for the Fe species of Fe SACs. The XANES characterization was also used to the exploration of dual metal site catalysts. He et al.^[^ ^29a^ ^]^ also explored the atomic structure of the Fe‐Co dual site by the XANES spectrums. The result showed that the oxidation state of Fe species in FeCo dual site catalysts was at between 0 and +3 by observing the edge position of the targeted sample. Moreover, the researchers further demonstrated the Co‐N coordination structure of Co species in FeCo dual site catalysts due to the same intensity and position of XANES spectrums. Additionally, XANES characterization technique is also applied to explore the atomic and electronic structure of other isolated metal species.^[^ ^6^, ^18^, ^26^, ^34^ ^]^

In addition to the oxidation state, the XANES is also used to explore the characteristic peak and electronic interaction between ADMSEs.^[^ ^25^, ^83^ ^]^ Zhao et al.^[^ ^25c^ ^]^ observed the same absorption edge position from the Fe species among the poly(FePc)with a weak‐field ligand I (PFePc‐I), strong‐field ligand (PFePc‐NCS), and Fe_2_O_3_ samples in the XANES spectrum. The result showed that the targeted Fe species had a similar valence state of +3 with the standard sample. Noteworthily, the researchers also proved the absence of electric dipole transition of 1s→4pz for the Fe species due to the transformation for the local coordination symmetry of Fe sites caused by the axial ligand. Similarly, Ni et al.^[^ ^83^ ^]^ obtained Fe K‐edge synchrotron radiation‐based XANES spectra to investigate the valence state and the local bonding symmetry of the isolated Fe site catalysts on defect‐rich graphene‐like porous carbon (DNG‐SAFe) (Figure 6b). The NG‐SAFe and defect‐rich DNG‐SAFe catalysts showed the similar and overlapping WL intensity in XANES spectrum, implying the similar valence states and coordination structures of Fe atoms. And the near‐edge absorption energies of NG‐SAFe and DNG‐SAFe lied in between the Fe foil and Fe_2_O_3_, which suggested that the valence state of Fe was between 0 and +3. It was worth noting that the small peak located at the energy of 7114 eV was observed, which could be ascribed to the transition of 1s to 4p_z_ and electron transfer between the metal atom and the ligand. The XANES is also used to preliminarily distinguish the difference of electronic properties between the metal/Pt and Pt site catalysts by combining the XPS characterization, facilitating the understanding for electronic structure and local coordination environment.^[^ ^66^ ^]^

#### 3.1.2 X‐Ray Absorption Fine Structure Spectroscopy Spectroscopy

To further reveal the geometry structure of ADMSEs, EXAFS is usually applied to investigate the local coordination information of the probed metal atom, including coordination numbers, bond length, and neighboring atomic species.^[^ ^51^, ^61^, ^87^ ^]^ The EXAFS is considered as a wave‐phase behavior, and the generation of EXAFS is closely related to the scattering of excited photoelectrons of the metal atoms and neighboring atoms. The obtained EXAFS raw data needs to be processed and interpreted by the Fourier transformation (FT) and wavelet transformation (WT).^[^ ^88^ ^]^ FT is a fundamental step to converse EXAFS signal into the radial structure function (R space), and separate backscattering atoms from the probed metal atoms by radial locations for investigating the coordination environment. By fitting the experimental data with the standard EXAFS equation, the quantitative local structures, such as coordination distance and the number of absorption atoms, are further obtained. In addition, the useful information directly obtained by FT‐EXAFS is the detection of coordination number changes, which is closely associated with the R‐space intensity. For example, Lien et al.^[^ ^84^ ^]^ studied the local coordination environments of isolated Co atoms in py‐B12 by the EXAFS spectrum. The EXAFS raw data was processed into FT‐EXAFS spectrum, and the bond length and coordination numbers of Co‐N sites in the prepared py‐B12 were 1.95 Å and 4 obtained from the FT k^3^‐weighted EXAFS spectrum, respectively (Figure 6c). Compared with the precatalytic states, the bond length of Co—N at the potential of 1–0.4 V versus reversible hydrogen electrode (RHE) elongated by 3%, which was mainly ascribed to the distortion of Co‐N_4_/C structure caused by the displacement of Co with oxygen‐containing species. For the r‐space, the Co‐N_4_‐O_2_ was considered as the most rational geometry structure obtained from the corresponding fitting data (Figure 6d). Similarly, the main peaks located at 1.41 and 1.68 Å were attributed to the first coordination shell of Co—N and Co—O from EXAFS, respectively.^[^ ^6c^ ^]^ The central Co metal atom coordinated with three N and one O atoms, and the corresponding DFT result also proved the stability of the Co‐N_3_O_1_ configuration. No metallic Fe—Fe bonds were observed from the FT‐EXAFS of high‐purity pyrrole‐type FeN_4_ catalysts (HP‐FeN_4_), highly according with the aberration‐corrected high‐angle annular dark field scanning transmission electron microscopy characterization (HAADF‐STEM) results of the monodispersed Fe sites anchored on the graphene substrates. In addition, the first‐shell fitting result of the FT‐EXAFS also demonstrated that the atomically dispersed Fe sites were coordinated with four N atoms, indicating the presence of the Fe‐N_4_ coordination configuration in HP‐FeN_4_ catalysts.^[^ ^44^ ^]^ Yang et al.^[^ ^85^ ^]^ also revealed the different coordination atoms of the isolated Fe sites by the EXAFS. Compared with the Fe—Fe (2.2 Å) and Fe—S (1.9 Å) of FeS and Fe foil, only the main peak located at 1.5 Å could be observed from the FT‐EXAFS spectrum, which was ascribed to the Fe—N(O) coordination (Figure 6e). Moreover, the researchers further proved that the atomically dispersed Fe sites were directly four N and one O atoms in Fe single atom catalysts.

In fact, there are some limitations for the FT‐EXAFS. Especially when the atoms making up the coordination shell are at the same distances, FT‐EXAFS will not be used to analyze the coordination environments of the probed atoms. However, WT‐EXAFS can provide a strong resolution in both R‐space and k‐space, and the atomic species of backscattering atoms can be deciphered even if the R space is overlapping, making up for the deficiency of the FT‐EXAFS.^[^ ^89^ ^]^ Xiao et al.^[^ ^63^ ^]^ reported a Fe single site on a highly graphitic nanosheet (Fe/N‐G‐SAC) catalyst for rechargeable Zn–air batteries, and revealed the geometry structure of FeN_4_ edge sites by EXAFS spectrum. Compared with the Fe foil and the Fe_2_O_3_, the main peak for the FT k^3^‐weighted Fe K‐edge EXAFS spectrum of the Fe/N‐G‐SAC was at 1.5 Å, and no metallic Fe—Fe bond could be found (2.2 Å). The WT‐EXAFS spectrum was conducted to identify the atomic distribution of isolated Fe species in the Fe/N‐G‐SAC, and the result was in good agreement with the FT‐EXAFS spectrum. The Fe foil and Fe_2_O_3_ had higher intensity of 8.2 Å^−1^, which belonged to the Fe—Fe metallic bonds. However, the main peak located at about a lower intensity of 5 Å^−1^ was observed from the WT of Fe K‐edge EXAFS oscillations, suggesting the presence of the isolated Fe atoms in the Fe/N‐G‐SAC (Figure 6f–h). The local coordination environment of isolated Co sites is also explored by EXAFS.^[^ ^86^ ^]^ The main peak at 2.2 Å could be clearly observed from FT‐EXAFS spectrum, corresponding to the first coordination shell (Co—N) (Figure 6i). Compared with many standard samples including Co foil, CoO, Co_3_O_4_, and CoPc, the WT‐EXAFS spectrum of Co‐N_5_ catalysts exhibited the maximum intensity at about 5 Å^−1^, which well corresponded the Co—N coordination bond, and no Co—Co metallic bond was found (Figure 6j–l). Qin et al.^[^ ^2a^ ^]^ reported a single‐atom Ru‐N‐C catalyst to reveal the effect of ligand fields on ORR performance. It was worth noting that the main peak at ∼1.59 Å from the FT‐EXAFS spectrum of Ru single atom catalysts was observed to be located at between the Ru—N peak (≈1.50 Å) of Ru single atom catalysts and the Ru—S bond (≈1.81 Å), which was, hence, ascribed to the Ru—N peak due to the great location difference between Ru—N and Ru—S bonds. Moreover, the WT‐EXAFS well distinguished the Ru—N bonds of the Ru single atom catalysts from the Ru—Ru bonds of Ru metal foil and the Ru—O bonds of Ru(acac)_3_ and Ru/ZIF‐8 by the intensity maximum, demonstrating the presence of isolated Ru sites. In addition, the EXAFS is widely applied to reveal the atomic structure of atomically dispersed dual site catalyst for ORR.^[^ ^55^, ^56^, ^90^ ^]^ Cui et al.^[^ ^90^ ^]^ constructed the isolated Fe‐Mn dual site catalysts (FeMn‐DSAC) for a flexible Zn–air batteries. No Fe—Fe, Mn—Mn, and Fe—Mn metallic bonds could be observed from the FT‐EXAFS spectrum of FeMn‐DSAC, and the dominant peaks located at 1.5 Å of Fe/Mn K‐edge were attributed to metal‐N coordination. The WT‐EXAFS was conducted to further investigate the bond length and coordination numbers of isolated Fe and Mn sites. Unlike the traditional MN_4_ configuration, the FeMn‐DSAC catalysts showed lower coordination numbers and bond length. Both Fe and Mn sites were coordinated with four N and two O, located on side‐on or two end‐on of metal centers. The result was also further proved by other characterizations.

### 3.2 Aberration‐Corrected Scanning Transmission Electron Microscopy

**Figure 7:**
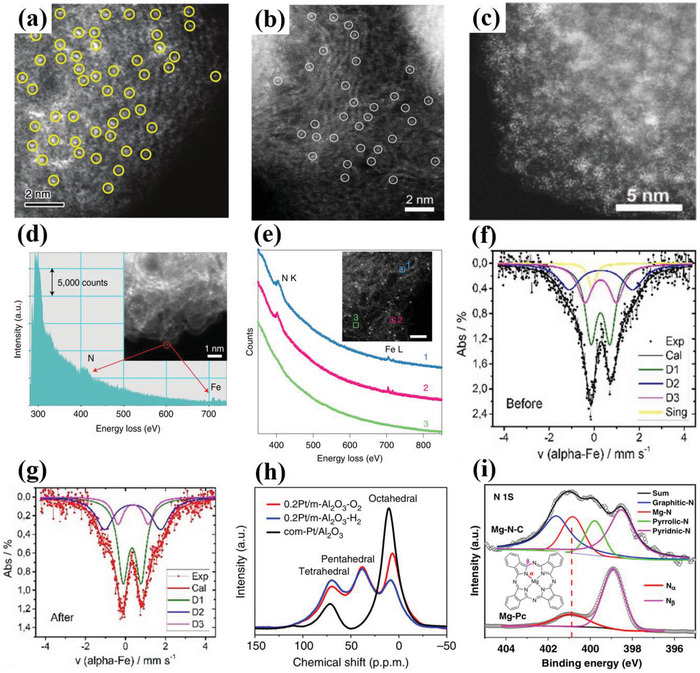
a) HAADF‐STEM image for the SA‐Fe‐NHPC. Single Fe atoms are highlighted by yellow circles. Reproduced with permission.^[^ ^77^ ^]^ Copyright 2020, Wiley‐VCH. b) HADDF‐STEM image of the CoNC. isolated Co sites are labeled with white circles. Reproduced with permission.^[^ ^48^ ^]^ Copyright 2021, American Chemical Society. c) HAADF‐STEM image of Cu/Zn@NC. Reproduced with permission.^[^ ^70a^ ^]^ Copyright 2021, Wiley‐VCH. d) EELS spectrum of three regions labeled by 1, 2, and 3 in the inset. Scale bar, 1 nm. The curves were offset for clarity. NK and FeL represent N K‐edge and Fe L‐edge, respectively. Reproduced with permission.^[^ ^6b^ ^]^ Copyright 2021, Springer Nature. e) EELS spectrum of the N k‐edge (N_k_) and Fe L‐edge (Fe_L_) obtained from a bright dot in the inset. Reproduced with permission.^[^ ^15a^ ^]^ Copyright 2021, Springer Nature. f) Moßbauer spectrum of the (Fe, Fe)_1_ electrocatalyst (before). g) Moßbauer spectrum of the (Fe,Fe)_1_ + N_2_/H_2_ (after). Reproduced with permission.^[^ ^95^ ^]^ Copyright 2016, American Chemical Society. h) The Al MAS‐NMR spectrum for 0.2Pt/m‐Al_2_O_3_‐O_2_, 0.2Pt/m‐Al_2_O_3_‐H_2_, and commercial Pt/Al_2_O_3_. Reproduced with permission.^[^ ^96^ ^]^ Copyright 2017, Springer Nature. i) The N 1s XPS spectrum for Mg‐N‐C and MgPc samples. Reproduced with permission.^[^ ^97^ ^]^ Copyright 2020, Springer Nature.

Traditional transmission electron microscopy (TEM) cannot characterize ADMSEs with atomic scale due to insufficient resolution. With the development of advanced electron microscope techniques, AC‐STEM shows higher resolution for characterizing the material. AC‐STEM with Å level resolution is a straightforward morphology to identify ADMSEs on account of its strong differentiating ability between the isolated metal sites and the support substrates.^[^ ^91^ ^]^ The large atomic number (symbol Z) difference between the isolated metal atom and the substrate is crucial for catalysts to be clearly visualized.^[^ ^92^ ^]^ Particularly, the HAADF‐STEM, by combining the AC‐STEM and the HAADF, can provide precise and direct observation for the isolated metal species on the support materials to further optimize the catalyst materials, which facilitates a better understanding for the real catalytic mechanism of ADMSEs.^[^ ^17^, ^93^ ^]^ For example, Chen et al.^[^ ^77^ ^]^ investigated the structural morphology of Fe sites on hierarchically porous carbon (SA‐Fe‐NHPC) catalysts via the HAADF‐STEM spectrum. The result exhibited the Fe species with Å level by yellow circles were evenly dispersed on the carbon support, indicating the presence of the isolated Fe metal sites (**Figure** **7**a). Tang et al.^[^ ^48^ ^]^ used the HAADF‐STEM spectrum to characterize the distribution of Co atoms in Co single atom catalysts. No nanoparticles or clusters were observed, and the isolated Co metal species by white circles were atomically dispersed on the onionlike graphitic layers (Figure 7b), which indicated the presence of Co single atom catalysts. Similarly, the morphology of isolated Co atoms was also observed by HAADF‐STEM spectrum.^[^ ^94^ ^]^ The Co species with atomic scale evenly dispersed on the heteroatoms‐doped carbon matrix (Co_1_‐N_3_PS/HC) were found, confirming the successful synthesis of atomically dispersed Co metal species in the Co_1_‐N_3_PS/HC.

Apart from the isolated site catalysts, the HAADF‐STEM spectrum is also applied to directly observe the dual metal site configuration for further optimizing the experimental conditions.^[^ ^15^, ^55^, ^70^ ^]^ Tong et al.^[^ ^70a^ ^]^ constructed the dual metal site catalyst with Cu‐N and Zn‐N sites (Cu/Zn‐NC) for exploring the intrinsic ORR mechanism. The HAADF‐STEM spectrum was conducted to investigate the morphological structure of Cu/Zn‐NC catalysts, and the highly dispersed atomic white dots could be directly observed, demonstrating the presence of the isolated Cu or Zn sites on the carbon support (Figure 7c). HAADF‐STEM spectrum is also used to identify different metal sites from ADMSEs. Han et al.^[^ ^15b^ ^]^ developed a Pt‐Fe dual site electrocatalyst (Fe‐N_4_/Pt‐N_4_@NC) for ORR. Many dual dots could be observed from the HAADF‐STEM spectrum of Fe‐N_4_/Pt‐N_4_@NC, suggesting the presence of atomically dispersed Pt or Fe sites. Noteworthily, due to the different contrasts of Pt and Fe atoms, the relatively brighter and darker dots could be ascribed to the Pt and Fe sites, respectively. In addition, the measured distance between the Pt and Fe sites was also considered as a stable catalyst structure. Similarly, Pt‐Fe dual metal site configuration could be also observed from HAADF‐STEM spectrum directly.^[^ ^55^ ^]^ The adjacent dual bright sites were found from the isolated Pt‐Fe dual sites dispersed on the carbon support, and the atomically dispersed Pt and Fe sites were further distinguished due to the different contrast of the Z‐contrast image from the Pt and Fe atoms.

Revealing the local atomic structure of the isolated metal atoms is crucial for understanding the catalytic mechanism of ADMSEs. HAADF‐STEM is usually conducted by coupling electron energy loss spectroscopy (EELS) to further understand the atom‐level chemical information of ADMSEs, such as the local chemical composition and bonding type.^[^ ^6^, ^18^, ^98^ ^]^ For example, the isolated Fe metal sites were evenly distributed in the few‐layer graphene sheet, and proved by the bright spots from the HAADF‐STEM image.^[^ ^99^ ^]^ The isolated Fe metal sites could be clearly observed due to the large Z value between the Fe metal atoms and the graphene substrates. Compared with the position 3 without bright spot in few‐layer graphene sheets, Fe and N elements could be directly observed in position 1 and 2 from the EELS spectrum, which well indicated the presence of Fe—N association, proving the Fe sites coordinated with N in graphene sheets. Jin et al.^[^ ^6b^ ^]^ also conducted the EELS spectrum to investigate the quantitative composition and chemical information of light Fe species in Fe‐N_4_ single‐atom catalysts (Fe‐N_4_ SACs) (Figure 7d). The spectrums of many regions with and without isolated metal sites identified by HAADF‐STEM result were shown, demonstrating the presence of Fe and N elements in both regions 1 and 2. However, no corresponding Fe and N elements could be observed from the EELS spectrum of region 3. This proved the coordinated environment of Fe‐N in Fe‐N_4_ SACs catalysts. The abundant dots could be directly observed from the HAADF‐STEM image, which further proved the proximity of Fe and N by the EELS spectrum (Figure 7e).^[^ ^15a^ ^]^ Moreover, the HAADF‐STEM coupled with EELS is also applied to further assist the interpretation of the catalytic mechanism.^[^ ^15^, ^18^ ^]^

### 3.3 Mössbauer Spectroscopy

Mössbauer spectroscopy is a fingerprint technique to characterize the coordination state, electron spin states, and electronic environments of metal species in catalysts.^[^ ^95^ ^]^ Especially for the Fe‐containing compounds, Mössbauer spectroscopy can be used to resolve the complex composition in compounds by detecting the changes of energy states for Fe nuclei, and effectively distinguish various Fe species with similar coordination environments.^[^ ^100^ ^]^ In addition, Mössbauer spectroscopy also can provide a more detailed understanding for the coordination environments of the isolated metal atoms by combining other characterization techniques.^[^ ^95^, ^101^ ^]^ For example, Kramm et al.^[^ ^95^ ^]^ investigated the local coordination environments of the (Fe,Fe)_1_ and (Fe,Fe)_1_ + N_2_/H_2_ catalysts by Mössbauer spectrums. Three doublets and a singlet could be observed from the (Fe,Fe)_1_ catalyst spectrum (before) (Figure 7f), while only two states appeared and the singlet disappeared in the spectrum after the purification treatment (Figure 7 g). The singlet corresponded to the small Fe particles, which implied that the inorganic Fe phase was removed after the purification. The three doublets corresponded to the FeN_4_ sites in different environments, resulting in one ferrous low spin (D1) and two ferrous medium spin (D2 and D3) sites. It was worth noting that the Mößbauer parameters of D2 and D3 were, respectively, closed to FePc and ferrous mid‐spin porphyrins, and the main difference between D2 and D3 was their local environment. In addition, the axial interaction leading to the formation of the doublet D2 might be a result from the action of N, and the pseudo‐sixfold coordination of Fe center with N might be the main reason that the Fe center didn't contribute highly to the improvement of ORR activity for the catalysts. Zhao et al.^[^ ^25c^ ^]^ revealed the role of axial ligands in the ORR catalytic activity of single Fe‐N site catalysts, and used the Mössbauer spectroscopy to prove the electron‐spin states of Fe species. Different electron‐spin states could be observed in both FePc and FePc‐I samples. Noteworthily, compared to the FePc, the FePc‐I showed a higher proportion of high‐spin Fe(III), implying that the Fe species could be transformed into a high‐valence and high‐spin state by the axial coordination. In addition, the Fe Mössbauer spectroscopy is also applied to understand the catalytic mechanism. Deng et al.^[^ ^101a^ ^]^ used the Fe Mössbauer spectroscopy to investigate the effect of the g‐C_3_N_4_ on the ORR catalytic performance. The corresponding Fe Mössbauer spectroscopy could be deconvoluted into low spin, mid‐spin, and high‐spin moieties, indicating the presence of Fe—N coordination. The Fe sites mixed with g‐C_3_N_4_ showed higher moiety contents than that of pure Fe sites, proving that the introduction of g‐C_3_N_4_ could facilitate the formation of Fe‐N_4_ moieties, which was closely related to the ORR activity. Jin et al.^[^ ^6b^ ^]^ applied the Mössbauer spectroscopy to reveal the origin of ORR activity, and proved that the enhanced catalytic activity related with site distance mainly originated from the variation of spin state of the Fe species. The Fe Mössbauer spectroscopy is also used to demonstrate the presence of O_2_ ligand on the sites (O_2_‐Fe(III)‐N_4_‐C_12_ moiety), indicating the e gas‐phase accessibility of Fe sites.^[^ ^15a^ ^]^

### 3.4 Other Characterizations

Apart from those characterization techniques mentioned above, other characterization techniques such as NMR, XPS, and FTIR are also applied to investigate the structural information and reveal the structure–activity relationship of catalysts.^[^ ^30^, ^60^, ^102^ ^]^

Solid state magic‐angle spinning NMR characterization has been conducted to probe the valuable structural information of metal species, since NMR can provide binding ligands of metal species and determine ADMSEs. For example, Zhang et al.^[^ ^96^ ^]^ performed the solid‐state magic‐angle spinning nuclear NMR (MAS‐NMR) spectrum to reveal the binding between the isolated Pt sites and the g‐Al_2_O_3_, and demonstrated the isolated Pt atoms were anchored on g‐Al_2_O_3_ support via oxygen bridges. Compared with commercial Pt/Al_2_O_3_, many main peaks located at 7, 38, and 70 p.p.m from NMR spectrums of 0.2Pt/m‐Al_2_O_3_‐H_2_ and 0.2Pt/m‐Al_2_O_3_‐O_2_ could be observed, which were attributed to tetrahedral (AlO_4_ at 7–10 p.p.m.), pentahedral (AlO_5_ at 38 p.p.m.) and octahedral (AlO_6_ at 70 p.p.m.) coordination, respectively (Figure 7 h). The pentahedrally coordinated Al^3+^ species that made up one third of Al^3+^ in m‐Al_2_O_3_ support were mainly formed in calcination and reduction processes. Especially after the NMR combined with XRD, TEM, and IR spectrums could prove the presence of isolated Pt sites on the mesoporous structure and surface area of support. Similarly, the solid‐state MAS‐NMR characterization was also conducted to prove the presence of atomically dispersed Pt sites, and indicated the close binding between the isolated Pt sites and the pentahedral coordination Al^3+^ of *γ*‐Al_2_O_3_ support by oxygen atoms.^[^ ^102^ ^]^

XPS characterization can measure the inner electron binding energy and the chemical shift of the atom more accurately than the auger electron energy spectrum. It provides not only chemical information of molecular structure and valence state, but also a variety of element and compound content, chemical state information, molecular structure, and chemical bond for electronic materials.^[^ ^2^, ^6^, ^15^ ^]^ For instance, Liu et al.^[^ ^97^ ^]^ revealed the valence state of the main‐group element via XPS characterization. The XPS spectrum of N 1s could be ascribed to four peaks, which well corresponded to the pyridinic‐N, pyrrolic‐N, Mg‐N*_x_*, and graphitic‐N (Figure 7i). The Mg 1s and 2p spectrum demonstrated the oxidation state of +2 for Mg, and it suggested the presence of Mg—N and Mg—C bonds. XPS characterization is also conducted to reveal the structure–activity relationship of catalysts. Yang et al.^[^ ^60^ ^]^ demonstrated the presence of C and N elements in defect carbon fiber (Co‐N@DCNF), and revealed that the 10Co‐N@DCNF (4.77%) had higher N content than that of 10CoN@CNF (3.5%), which indicated N species were closely related to the defected structure of carbon. In addition, the main three peaks from the N 1s spectrum located at 398.4, 401.0, and 399.2 eV could be deconvoluted into pyridinic‐N, graphitic‐N, and Co—N*_x_* respectively, and the 10Co‐N@DCNF showed Co—N*_x_* content of 14.43% compared with the 10CoN@CNF (3.91%), which was of vital importance for regulating the catalytic activity. Furthermore, XPS is also used to demonstrate the presence of C—OH functional groups by combining NMR.^[^ ^6c^ ^]^

FTIR is an effective mean to directly monitor the adsorption behaviors of probe host molecules such as CO and NH_3_ on the surface of the catalyst. According to the vibration frequency and intensity of the adsorbed molecules, the characteristic and local structure of the active center can be inferred by proper calibration. When the sample is placed in the optical path of the interferometer, the energy of some frequencies is absorbed, and then the intensity curve of the obtained interferogram changes correspondingly. Through mathematical Fourier transform, each frequency on the interferogram can be converted into the corresponding light intensity. Besides, FTIR can be used to verify the functional groups and the chemical structures of unknown support substrates, and determine the presence of active sites and various intermediates produced during catalytic reactions.^[^ ^30^, ^103^ ^]^

## 4 Synthesis Methods for Atomically Dispersed Metal Site Electrocatalysts

The exploration and development of controlled synthesis methods are the prerequisite for constructing the advanced ADMSEs.^[^ ^24^, ^104^ ^]^ The demand for high‐efficiency electrocatalysts leads to the precise design and synthesis of ADMSEs. At present, ADMSEs are still facing many difficulties for catalyst preparation due to the high surface free energy of isolated metal atoms, which easily causes the aggregation of metal active centers due to the weak interaction between the active metal atoms and the support materials.^[^ ^24^, ^105^ ^]^ A major challenge for the preparation of ADMSEs is to inhibit the aggregation of metal species in synthetic and catalytic reactions.^[^ ^105b^ ^]^ In general, the ideal ADMSEs should have the following several advantages: i) Suitable metal loading for avoiding aggregation into particles; ii) Good dispersion for metal active sites and stable structure for support materials; iii) Strong interaction between the isolated metal atoms and the support materials. Until now, various synthesis strategies have been developed for ADMSEs. The main synthesis strategies can be divided into two types, including bottom‐up and top‐down strategies. The bottom‐up strategy mainly involves the adsorption, reduction and restriction of the targeted metal precursors for the synthesis of ADMSEs. Generally, wet chemistry method, ball milling and atomic layer deposition method (ALD) are classified into bottom‐up strategies.^[^ ^106^ ^]^ For the top‐down strategy, ADMSEs are mainly formed by breaking metal‐metal bonds from bulk metals or metal nanoparticles as precursors into smaller pieces at atomic level, including high‐temperature pyrolysis method and chemical vapor deposition method (CVD) (mainly involving gas‐phase migration method).^[^ ^104^, ^106^, ^107^ ^]^ In the following section, some efficient methods for ADMSEs from the above two synthesis strategies will be discussed.

### 4.1 Wet Chemistry Method

The wet chemistry method is considered as one of the most promising synthesis methods for ADMSEs due to its simplicity of operation, the absence of special equipment and the facilitation of mass production.^[^ ^24^, ^108^ ^]^ As a typical bottom‐up strategy, one of the most important goals for this method is to prepare ADMSEs with well‐defined monodispersity and high metal loading. As a rule, the method for preparing ADMSEs mainly includes three basic processes. First, the metal precursors are introduced and absorbed on the substrates by impregnation and co‐precipitation, and the solvent is then removed by drying treatment. Finally, the reduction and activation processes are implemented by calcination treatment, enhancing the interaction between the monodispersed metal atoms and the substrates.^[^ ^47^, ^109^ ^]^ The strong interaction achieved by chemical coordination between the target metal species and the support materials can effectively hinder the aggregation and improve the dispersion for metal atoms.^[^ ^105b^ ^]^ In fact, due to the high surface free energy for ADMSEs, researchers prefer to reduce the target metal species loading and anchoring site density on the substrate to ensure the successful preparation of ADMSEs.^[^ ^24^, ^105^ ^]^ However, these means obviously decrease the number of accessible active sites on substrates, and thus affect the catalytic activity to some extent. Therefore, developing an effective method is vitally significant for the synthesis of the advanced ADMSEs. Several approaches such as impregnation, co‐precipitation, and spatial confinement methods about wet chemistry method will be discussed separately as follows.

#### 4.1.1 Impregnation Method

**Figure 8:**
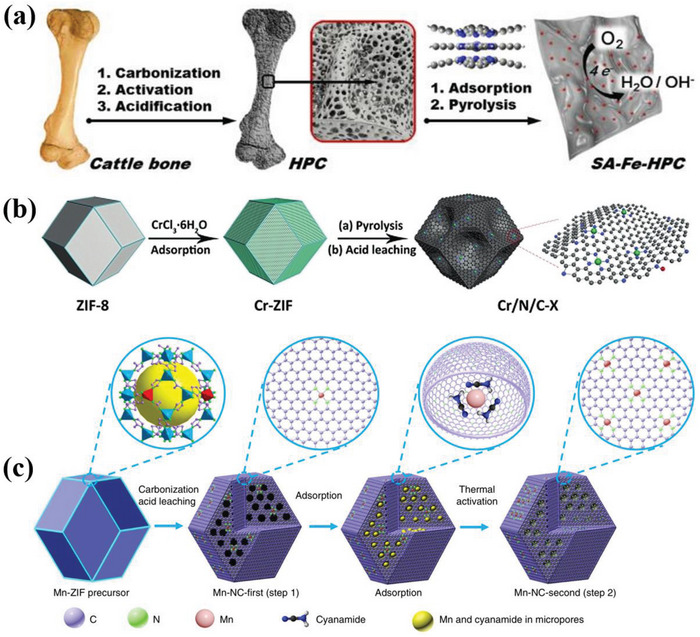
a) The synthetic process for SA‐Fe‐HPC electrocatalyst. Reproduced with permission.^[^ ^111^ ^]^ Copyright 2018, Wiley‐VCH. b) Schematic illustration of Cr SACs electrocatalyst. Reproduced with permission.^[^ ^112^ ^]^ Copyright 2019, Wiley‐VCH. c) Schematic illustration for synthetic process of MnN_4_ site electrocatalyst. Reproduced with permission.^[^ ^113^ ^]^ Copyright 2018, Springer Nature.

In the synthesis process, the precise controlling of the target metal loading and the selectivity of support are key to the successful preparation of the ADMSEs.^[^ ^14^, ^19^, ^110^ ^]^ As a simple and mild synthesis method, the impregnation method has been widely applied to prepare ADMSEs due to the advantage of easily achieving the large‐scale production without specialized equipment. At present, many efforts have been devoted to the development of ADMSEs for ORR via the impregnation method.^[^ ^44^, ^111^ ^]^ For example, the Pt_1_ grafted complexes of Fe, nitrogen and carbon (Pt_1_@Fe‐N‐C) multifunctional electrocatalyst were obtained with iron acetate and chloroplatinic acid hexahydrate as the metal precursors.^[^ ^110^ ^]^ The Fe‐N‐C catalysts were first obtained by simple mixing, and further annealed at 1000 and 900 °C at Ar and NH_3_ atmosphere, respectively. The Pt_1_@Fe‐N‐C electrocatalysts were subsequently produced by the transfer of a Pt atom onto the Fe center with oxygen molecule bridging by mild pyrolysis treatment, and the Pt_1_‐O_2_‐Fe_1_‐N_4_ active moieties effectively protected Fe active sites and prevented the loss of Fe sites under the harmful environment. In addition, the unique active moieties further regulated adsorption energy and improved reduction kinetics. Similarly, atomically dispersed Fe on hierarchical porous carbon (SA‐Fe‐HPC) was developed for ORR by using the impregnation method.^[^ ^111^ ^]^ The whole synthesis process mainly included the preparation of the supports and the loading of single Fe atoms (**Figure** **8**a). The HPC support was first obtained by a series of carbonization and acid‐washing processes, and then SA‐Fe‐HPC electrocatalysts were mainly obtained by the dispersion of isolated Fe atoms and subsequent pyrolysis treatment of unsubstituted phthalocyanine/iron phthalocyanine complexes on the HPC. The XAS and Mössbauer spectroscopy showed that the Fe atoms were coordinated with N in HPC, indicating the successful synthesis of the isolated Fe site catalysts. The enhanced ORR activity for SA‐Fe‐HPC electrocatalysts was mainly ascribed to the good dispersion of isolated Fe metal sites with the optimal coordination environment. Impregnation method is also applied to synthesize the high‐purity pyrrole‐type FeN_4_ sites for ORR. Unlike the traditional FeN_4_ site catalysts (Ar atmosphere), FeCl_3_ as metal precursor was dispersed on Ketjenblack ECP‐600 JD support with polymerization of aniline, and pyrrole‐type FeN_4_ sites catalysts were obtained by high‐temperature pyrolysis treatment at NH_3_ atmosphere.^[^ ^44^ ^]^ In addition, The Fe and Ni atoms were impregnated into the ordered porous carbon with ordered polystyrene as a template, which effectively realized the rapid mass transfer of all accessible isolated active sites. The Fe/Ni dual atom catalysts were fixed on the carbon support by Fe‐N_4_ and Ni‐N_4_ coordination bonds, and showed excellent ORR activity.^[^ ^20a^ ^]^

#### 4.1.2 Co‐precipitation Method

Co‐precipitation is also an effective method for preparing ADMSEs on account of the easy operation during the synthesis process.^[^ ^24^, ^106^ ^]^ Unlike the impregnation method mentioned above, co‐precipitation method mainly involves the simultaneous precipitation of several metal species in chemical reaction. The ratios of different metal precursors are crucial to the successful preparation of ADMSEs, which greatly affects the formation of each metal species in the catalysts. For example, co‐precipitation method was applied to prepare the Pt_1_/FeO*_x_* with chloroplatinic acid and ferric nitrate as metal precursors.^[^ ^14^ ^]^ In chemical reaction, under the condition that the PH value was well maintained at about 8, the Pt/Fe atomic ratio was precisely controlled to achieve the anchoring of monodispersed Pt atoms on the surfaces of FeO*_x_* nanocrystallites, and the Pt_1_/FeO*_x_* catalysts with 0.17% Pt loading were obtained by subsequent annealing treatment. Especially when the Pt/Fe atomic ratio was increased to 1:95, the Pt particles could be clearly observed. The isolated Pt atoms were coordinated with O atoms of FeO*_x_* supports, forming the Pt‐O bonds. The introduction of Pt single atoms effectively reduced the adsorption energy and enhanced the catalytic kinetics for the target molecules, and the charge transfer between the isolated Pt atoms and the FeO*_x_* could further enhance the metal‐support interaction, stabilizing Pt atoms on the supports. For another example, Ir_1_/FeO*_x_* catalysts were obtained by the co‐precipitation process of H_2_IrCl_6_ and Fe(NO_3_)_3_ with appropriate ratios.^[^ ^114^ ^]^ The Ir atoms of 0.01 wt% were added and anchored on surface of FeO*_x_* supports, and no Ir nanoparticle could be observed, indicating that this synthesis strategy could achieve the well‐defined dispersion of Ir single atoms on the FeO*_x_* supports. Moreover, the presence of Ir single atoms greatly enhanced the reductivity of FeO*_x_* supports and the formation of oxygen vacancies, which made the Ir_1_/FeO*_x_* catalysts have excellent performance. Despite huge advances that have been made for co‐precipitation strategy, many metal sites are usually buried on the noncarbon support, causing insufficient catalytic performance, which is not suitable for direct use in electrocatalytic reactions.

#### 4.1.3 Spatial Confinement Method

The spatial confinement method have widely been used as an effective approach for the preparation of ADMSEs.^[^ ^105^, ^115^ ^]^ In general, the basic principle of this strategy is mainly concerned with the spatial confinement of metal atoms in built‐in pores or cavities to stabilize the isolated metal atoms on supports for further inhibiting the migration and aggregation of metal atoms, generating the isolated metal sites of high density.^[^ ^116^ ^]^ The strategy mainly involves two basic steps: i) Achieving the uniform dispersion of metal precursors in porous materials; ii) Stabilizing the atomically dispersed metal species on supports by removing ligands of precursors.^[^ ^117^ ^]^ For instance, Luo et al.^[^ ^112^ ^]^ reported a single‐atom Cr‐N_4_ site electrocatalyst for ORR by using metal organic framework (MOF) as an anchoring matrix (Figure 8b). Cr precursors were first adsorbed and confined in micropores of zeolitic imidazolate frameworks (ZIF) by host‐guest interaction. Instead of replacing Zn nodes, the isolated Cr atoms were only trapped in ZIF‐8, which was proved by the lack of the Cr‐N scattering path. During this synthesis process, the local coordination environment of Cr was changed from the CrCl_3_·6H_2_O, and the second Cr‐O‐Cr path could be observed. The Cr single atom catalysts were not obtained directly, but through the hydrolysis process in micropores of ZIF‐8. One‐pot mechanochemical method was applied to develop the Fe‐N site catalysts (Fe‐N/C) by spatial confinement of ZIF.^[^ ^116c^ ^]^ During the synthesis process, the isolated Fe sites were confined into the built‐in cavities of ZIF matrices, ensuring the successful anchoring of isolated Fe sites on carbon support during the high‐temperature pyrolysis. A two‐step doping and adsorption method was proposed to obtain the atomically dispersed Mn catalysts for ORR, and this strategy effectively increased MnN_4_ site density in the graphitized carbon host from ZIF‐8 by the subsequent spatial confinement method (Figure 8c).^[^ ^113^ ^]^ The Mn‐doped ZIF was first carbonized into the partially graphitized carbon support, and the Mn and N sources were further transferred into the 3D carbon host via subsequent thermal activation. Lots of bright spots could be clearly observed from HAADF‐STEM images, indicating the uniform dispersion of the isolated Mn metal sites of high density in 3D carbon host. Moreover, the XAS and EELS characterizations also proved the co‐existence of the Mn‐N coordination. Similar works are also reported about ZIF.^[^ ^116^, ^118^ ^]^ Apart from ZIF, carbon nanotubes are also used as the carbon host to prepare the ADMSEs by spatial confinement strategy, and the ADMSEs are obtained via subsequent pyrolysis treatment.^[^ ^119^ ^]^ In addition, the template‐assisted confinement strategy has also been reported for the preparation of ADMSEs.^[^ ^119^, ^120^ ^]^ A Si‐coating‐mediated synthetic strategy was applied to obtain the Fe site catalysts with rich Fe‐N_x_ sites, and this strategy could well prevent the generation of less active Fe and Fe_3_C species. Moreover, this strategy could be also broadened to different Fe and N precursors, effectively confining and stabilizing the isolated Fe sites in the carbon support during the high‐temperature pyrolysis.^[^ ^120^ ^]^ Similarly, the mesoporous‐Si‐protected strategy was also used to suppress the aggregation of metal atoms.^[^ ^119c^ ^]^ During the synthesis process, the mesoporous Si shell was coated on the surface of Zn,Co‐ZIF matrices by the hydrolysis of tetraethylorthosilicate in alkaline medium. From the demonstration of many characterizations, the presence of the mesoporous Si shell not only effectively confined and stabilized the single Co sites, but also significantly changed the pore structure including the specific surface area and pore volume during the high‐temperature pyrolysis. In addition, soft‐template surfactants are also proved to be effective for inhibiting the aggregation of metal sites for ADMSEs.^[^ ^121^ ^]^

### 4.2 High‐Temperature Pyrolysis Method

The high‐temperature pyrolysis is one of the effective top‐down approaches to prepare ADMSEs for ORR.^[^ ^82^, ^122^ ^]^ The strategy mainly involves the thermal decomposition of different precursors at different gas atmospheres (e.g., N_2_, H_2_, NH_3_, and A_r_) and temperatures (about 600–1200 °C), and easily leads to different atomic structures, local coordination environments, and electronic structure, affecting the catalytic performance of ADMSEs.^[^ ^37^, ^105^ ^]^ As for the synthesis strategy, the vital focus mainly involves inhibiting the agglomeration of metal atoms and ensuring the monodispersity of metal atoms under the high‐temperature environment for ADMSEs. According to the order of metal atoms in precursors, the high‐temperature pyrolysis strategy can be classified into two types, including metal‐containing specific complexes and metal‐containing irregular complexes.

#### 4.2.1 Metal‐Containing Specific Complexes

As one of the most potential metal‐containing specific complexes, MOF has been greatly developed for the application of ADMSEs due to its unique porous structure and diverse compositions.^[^ ^105^, ^122^ ^]^ MOF, as a crystalline porous material, is mainly composed of metal ions and multifunctional organic linkers. The interconnection inside the cages at a molecular scale makes them accessible to small molecules, and the precise control for the formation of the porous carbon support derived from MOF can effectively regulate catalytic performance.^[^ ^105a^ ^]^ The MOF contains a large number of metal nodes and ligands, and ADMSEs can be prepared by replacing the original metal nodes with target metal atoms.^[^ ^116a^ ^]^ The MOF‐derived materials can provide more anchoring sites for target metal atoms, and the interaction between the target atoms and the ligands can effectively ensure the monodispersity of the target metal atoms.^[^ ^104^, ^116^ ^]^ Particularly, the MOF‐derived materials have high surface area, porous structure, N‐containing coordination environment and monodispersed metal sites after high‐temperature pyrolysis treatment. Among a variety of MOF materials, ZIF materials are commonly considered as the new platform for developing ADMSEs, such as Co‐based ZIF‐67 and Zn‐based ZIF‐8. During the pyrolysis, the MOF materials can provide more anchoring sites for the doped metal atoms, and make the monodispersed metal atoms stabilize in the MOF‐derived support materials.^[^ ^48^, ^69^ ^]^ In addition, high‐temperature pyrolysis treatment easily leads to the reduction of metal nodes by carbonization of the organic linkers of MOF, and the number of N coordination for the M‐N*_x_* sites also changes with pyrolysis temperature, resulting in the formation of the coordinately unsaturated state for ADMSEs. At present, the strategy for ADMSEs from MOF‐derived materials can be divided into the following two ways according to the positions of the target metal atoms, which are the target metal atoms at metal nodes and ligands of MOF.

**Figure 9:**
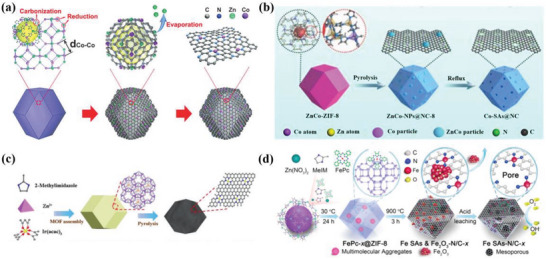
a) The synthetic process of Co SAs/N‐C catalyst. Reproduced with permission.^[^ ^116a^ ^]^ Copyright 2016, Wiley‐VCH. b) Schematic depiction of synthetic process for Co‐SAs@NC catalyst. Reproduced with permission.^[^ ^11a^ ^]^ Copyright 2019, Wiley‐VCH. c) Schematic depiction of host‐guest synthesis strategy for Ir single atom catalyst. Reproduced with permission.^[^ ^69b^ ^]^ Copyright 2019, Wiley‐VCH. d) Schematic depiction of the preparation process for Fe single atom catalyst. Reproduced with permission.^[^ ^50^ ^]^ Copyright 2018, American Chemical Society.

As for the strategy that the target metal atoms are at the metal nodes of MOF, the MOF‐derived materials mainly involve TM atoms due to the strong coordination. For instance, Yin et al.^[^ ^116a^ ^]^ developed a single Co atom catalyst on nitrogen‐doped porous carbon (Co SAs/N‐C catalyst) for ORR by the high‐temperature pyrolysis method (**Figure** **9**a). The bimetallic Zn/Co MOFs were synthesized with Co(NO_3_)_2_·6H_2_O and Zn(NO_3_)_2_ as the metal precursors, and a large number of Co and Zn metal nodes were formed. The N‐doped porous carbon was formed after pyrolysis treatment, and Zn could be selectively evaporated above 800 °C. The presence of Zn atoms not only changed the distance between adjacent Co atoms, but also provided more accessible N coordination atoms, stabilizing the isolated Co atoms in the N‐doped porous carbon support. Noteworthily, low‐temperature pyrolysis treatment could ensure the presence of isolated Co sites, and coordinate with two and four N coordination for 800 and 900 °C, respectively. In another example, the size effect was studied by regulating the atomic ratios of Zn/Co for ORR.^[^ ^11a^ ^]^ During the synthesis process, the bimetallic ZnCo‐ZIF precursors were carbonized at 900 °C for 2 h under Ar atmosphere, the N‐doped porous carbon with mesoporous size of 3–4 nm was formed (Figure 9b). Noteworthily, the mixed Zn acted as a “fence” to widen the distance between the adjacent Co atoms. The Zn and Co metal nodes were significantly reduced during the pyrolysis, and especially, Zn ions could be selectively evaporated to form Co single atom catalysts on N‐doped carbon (Co‐SAs@NC). Low Zn/Co atomic ratios (such as 0:1 and 2:1) easily caused a smaller space between the adjacent Co atoms after the pyrolysis, causing the aggregation of Co atoms into clusters or nanoparticles. However, the evaporation of Zn ions from a higher Zn/Co atomic ratio (8:1) could provide sufficient N anchoring sites, ensuring the presence of isolated Co sites. In addition, isolated Fe sites were also modified on the SiO_2_‐induced MOF template, and the MOF covered with SiO_2_ might produce an outward adsorption force, resulting in anisotropic thermal contraction of the MOF precursor.^[^ ^123^ ^]^ The edge of ZIF‐8 could be maintained during pyrolysis, and Fe^3+^ could be reduced by the obtained N‐doped carbon and bonded with adjacent N/C atoms to form the Fe‐N_4_‐C site.

Unlike the TM atoms, noble metal atoms located at ligand nodes of MOF are also reported. Xiao et al.^[^ ^69b^ ^]^ synthesized an Ir single site catalyst (Ir‐SAC) for high‐efficiency ORR catalysis. During the synthesis, ZIF‐8 was used as support precursor, Ir atoms were encapsulated in organic inkers of ZIF (Ir‐ZIF‐8), and the Ir‐ZIF‐8 crystalline had good crystal texture and structural homogeneity. Moreover, Ir‐ZIF‐8 precursor with a cavity diameter of 11.6 Å was pyrolyzed at 950 °C under Ar/H_2_ atmosphere, and Ir metal atoms were stabilized in abundant N anchoring sites from the MOF, forming Ir single atom catalysts (Ir‐SAC) (Figure 9c). The Ir‐SAC catalysts showed superior ORR catalytic activity than the previously reported SACs and commercial Pt/C. Similarly, Ru atoms were also encapsulated in organic inkers of MOF to investigate the effect of the ligand field for ORR.^[^ ^2a^ ^]^ During the synthesis process, the Ru^3+^ and Zn^2+^ ions were mixed with 2‐methylimidazole, and the metal precursors were trapped, encapsulated, and further self‐assembled into Ru/ZIF‐8 by the hydrothermal method. The Ru/ZIF‐8 intermediates were transformed into Ru single atom catalysts by high‐temperature pyrolysis strategy, and the introduced S coordination during the reaction process also further ensured the synthesis of Ru single atom catalysts.

As for the synthesis of ADMSEs derived from MOF, the metal atoms tend to agglomerate into clusters or nanoparticles during high‐temperature pyrolysis. Particularly, some metal atoms are buried in dense carbon matrix, causing the reduction of the exposure and accessibility of the active sites and low catalytic efficiency. Regarding this issue, acid leaching treatment is usually considered as an effective strategy to ensure the successful synthesis of ADMSEs. Jiang et al.^[^ ^50^ ^]^ developed an atomically dispersed Fe‐N_4_ site catalyst for ORR electrocatalysis (Figure 9d). ZIF‐8 materials were used as the support precursors, and the researchers selectively cleaved the C—N bond and stabilized the Fe atoms by porosity structure derived from ZIF‐8, forming the edge‐hosted Fe‐N_4_ moieties. And then the acid leaching strategy was further used to maintain the presence of atomically dispersed Fe sites by removing the free metallic residues. The Fe single atom catalysts showed a superior *E*_1/2_ of 0.915 V versus RHE and an atom‐utilization efficiency for ORR. Similarly, the Fe(acac)_3_ acted as the metal precursor and was encapsulated in ZIF‐8.^[^ ^124^ ^]^ The atomically dispersed Fe site catalysts (Fe—N—C) were obtained by direct high‐temperature pyrolysis and subsequent acid‐leaching treatment. The strategy could enhance the explosion and atom‐utilization of active sites, which effectively improved the ORR catalytic activity.

#### 4.2.2 Metal‐Containing Irregular Complexes

**Figure 10:**
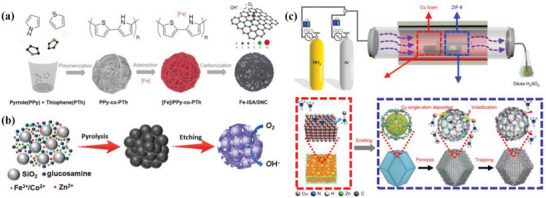
a) The preparation process of Fe‐ISA/SNC catalyst. Reproduced with permission.^[^ ^126^ ^]^ Copyright 2018, Wiley‐VCH. b) Schematic depiction of the preparation process for hierarchically porous Co/Fe single atom catalysts. Reproduced with permission.^[^ ^118^ ^]^ Copyright 2018, Wiley‐VCH. c) Schematic diagram of the device and preparation process of Cu‐SAs/N‐C. Reproduced with permission.^[^ ^129^ ^]^ Copyright 2018, Springer Nature.

As one of the most effective ways to construct ADMSEs, metal‐containing irregular complexes have been widely concerned. Many N‐containing complexes and polymers are usually used as carbon sources, and the induced metal atoms can be confined in the anchoring sites during the pyrolysis, including glucose, dicyandiamide, and melamine.^[^ ^17^, ^85^, ^125^ ^]^ Wu et al.^[^ ^125a^ ^]^ reported a single‐atom Cu catalysts (Cu‐N@C) with Cu(I)‐N active sites within graphene for ORR. In this work, Copper phthalocyanine and dicyandiamide respectively used as the metal and carbon support precursors were uniformly mixed for 24 h, and the Cu‐N@C catalysts were obtained by direct pyrolysis under 800 °C for 2 h at Ar atmosphere. As an electrocatalyst, Copper phthalocyanine was not sensible for ORR due to its saturated coordination structure. Noteworthily, the researchers broke the coordination balance of Copper phthalocyanine by direct pyrolysis treatment with dicyandiamide, and the coordination unsaturated Cu(I)‐N structure was proved as the active site for ORR. A novel copolymer pyrolysis strategy is also used to obtain isolated Fe site catalysts coordinated with N and S atoms for ORR.^[^ ^126^ ^]^ The first step for the synthesis process was the copolymer of pyrrole and thiophene. The black pyrrole‐thiophene copolymer (PPy‐*co*‐PTh) precipitate was then prepared by mixing tetrafluoroboric acid‐diethyl ether complex and pyrrole, and Fe atoms were finally adsorbed and stabilized in polymer nanostructure (**Figure** **10**a). During the pyrolysis, the polymer was carbonized, Fe atoms were confined in support by connecting the N and S atoms, and no Fe—Fe metal bond could be observed. Li et al.^[^ ^127^ ^]^ used the *o*‐phenylenediamine precursors as carbon sources to synthesize the ultrahigh‐loading Zn single atom catalysts and showed superior catalytic activity for ORR. In addition, other carbon materials coordinated with polymer for the synthesis of ADMSEs are also reported widely.^[^ ^99^, ^116^ ^]^ The template method is also a potential approach to prepare ADMSEs for ORR, mainly because the derived 3D porous structure from pyrolysis treatment can provide a large specific surface area, abundant active sites and efficient mass transfer.^[^ ^118^ ^]^ A bimodal template‐based strategy was used to prepare Fe single site catalyst for ORR, and FeCl_3_, ZnCl_2_, and d‐glucosamine respectively used as metal precursors and carbon sources were mixed. The resulting products were freeze‐dried and pyrolyzed, and the added SiO_2_ as a hard template was removed by HF etching for 12 h (Figure 10b). Noteworthily, ZnCl_2_ and SiO_2_ led to the formation of porous carbon. Zn ions were evaporated to create the hierarchically porous structure during the pyrolysis, the removal of SiO_2_ template also induced the porosity, and FeN_2_ was identified as the active site. Similarly, Wu et al.^[^ ^128^ ^]^ also used SiO_2_ as the sacrifice template and dispersed Fe atoms on N and S‐doped hierarchical porous carbon (Fe_1_/N,S‐PC) for ORR. The Fe_1_/N,S‐PC electrocatalysts were synthesized by a two‐step pyrolysis process, including pyrolysis treatment at 600 °C under H_2_/Ar flow and 900 °C under N_2_ flow after the removal of SiO_2_. (NH_4_)_2_S_2_O_8_ and K_3_[Fe(CN)_6_] were used as S and Fe precursors, since the (NH_4_)_2_S_2_O_8_ could induce S source and produce the porous structure during the pyrolysis, forming Fe_1_/N,S‐PC catalyst.

### 4.3 Other Synthesis Methods

Apart from the synthesis strategies mentioned above, other effective synthesis methods have also been reported to construct ADMSEs such as ball milling method, CVD method, and ALD method, which can further accelerate the development of efficient ORR electrocatalysts.^[^ ^15^, ^24^, ^130^ ^]^

Ball milling method as a facile and powerful strategy can efficiently break the binding between the metal atoms, and trap the isolated metal atom on the support substrate for synthesizing ADMSEs. Zhao et al.^[^ ^17a^ ^]^ developed the Fe single atom catalysts (Fe‐NC SAC) for ORR by virtue of ball milling method. The strategy could achieve the well‐defined dispersity of metal atoms, and made the metal atoms stably anchor on porous carbon substrates by chelation of various N sources. The atomically dispersed Fe species with Å level from Fe single site catalysts were observed from HAADF‐STEM characterization, and the Fe‐N*_x_* moieties were found from the N 1s of XPS. In addition, the XAFS characterization also showed the absence of the Fe—Fe bond, further proving the successful synthesis of Fe single site catalysts. Moreover, the strategy could be extended to the synthesis of different M‐N*_x_* catalysts (Mn, Co, Ni, Cu, Mo, and Pt), and achieve the anchoring of high‐loading central metal atoms on substrates. The abundant Cu metal sites were also anchored on the N doped 2D carbon substrates (Cu—N—C) by ball milling method.^[^ ^47^ ^]^ The result showed that Cu metal atoms coordinated with N atoms, and no Cu—Cu bond could be observed. Additionally, the Cu‐N_2_ and Cu‐N_4_ mixed structure were found on Cu—N—C catalysts, indicating the presence of Cu single atom catalysts. Ball milling method is also applied to obtain dual atom catalysts containing isolated Fe and Se sites, exhibiting high‐efficiency ORR performance.^[^ ^131^ ^]^ In addition, ball milling method can also be used to achieve the mass production of ADMSEs.^[^ ^132^ ^]^

CVD is another powerful strategy to precisely prepare ADMSEs by adjusting related experimental conditions. Especially for the gas‐phase migration strategy, the in situ pyrolysis process strategy can effectively avoid unnecessary mechanical mixing of metal species and carbon supports, since the migrated atomic metal species are trapped by supports to form strong metal‐support interactions, thereby increasing the number of effective active sites and maintaining uniform dispersion of metal sites simultaneously.^[^ ^15^, ^133^ ^]^ In addition, the gas‐phase migration strategy is capable of achieving large‐scale production of ADMSEs at industrial levels.^[^ ^129^ ^]^ Qu et al.^[^ ^129^ ^]^ developed an NH_3_‐assisted gas‐migration strategy for the direct conversion of bulk Cu_2_O to a monatomic Cu catalyst at the gram scale (Figure 10c). Under high temperature and Ar atmosphere, ZIF‐8 was thermally pyrolyzed to form an N‐doped carbon carrier with a large number of defect sites. Based on strong Lewis acid‐base interaction, Cu atoms on the surface of Cu foam formed volatile Cu(NH_3_)*_X_* species with NH_3_, which were captured by defects in N‐doped carbon carriers to form isolated copper sites, obtaining an atomically dispersed Cu site catalysts. The ICP‐AES test showed that the loading of Cu atoms was 1.26% from Cu single atom catalysts, and the calculated surface coverage of Cu atoms reached 0.06 atoms nm^−2^. These characteristics made this catalyst especially suitable for ORR reaction. Gas‐phase migration strategy was used to prepare an isolated Fe site catalyst (FeNC‐CVD‐750) for ORR by replacing the Zn atom with flowing iron chloride vapor, achieving the full utilization of active sites.^[^ ^15a^ ^]^ Abundant Zn‐N_4_ sites were changed into Fe‐N_4_ sites, and anchored on the porous carbon substrates. The high‐resolution N 1s XPS spectrum from FeNC‐CVD‐750 was attributed to N‐O*_x_*, N^+^, N_gr_‐N, N‐H, M‐N*_x_*, and pyrinic N, and proved the presence of abundant Fe‐N*_x_* moieties by combining EELS spectroscopy and atomic‐resolution HAADF‐STEM characterizations. It was remarkable that this strategy could achieve a high active site density of 1.92 × 10^20^ sites g^−1^ and active site utilization of 100% for ORR catalysis. Moreover, the commercial CuO power was also sublimated into the isolated Cu site catalysts (Cu ISAS/NC), and the Cu vapor is transferred and confined on the defect‐rich N‐doped carbon substrate.^[^ ^64^ ^]^ A series of characterizations were conducted to prove the presence of isolated Cu metal sites, and no Cu—Cu bond could be observed in Cu ISAS/NC. Electron paramagnetic resonance (EPR) result also proves the coordinatively unsaturated states of the isolated Cu species. Furthermore, this strategy is also extended to the preparation of other single site catalysts by changing metal oxide precursors. Similarly, Zhou et al.^[^ ^133a^ ^]^ developed the atomically dispersed Mn sites as efficient catalysts for ORR by gas‐migration strategy. Under the evaporation of MnCl_2_·4H_2_O at low temperature, the carbonization of ZIF‐8 was completed by a rapid heating process, which was accompanied by the formation of the atomic Mn‐N*_x_* active sites. This strategy not only significantly increased the Mn loading, but also made Mn atoms evenly dispersed throughout the carbon skeleton, thus promoting the exposure of Mn‐N*_x_* active sites.

ALD method is a potential strategy to make the metal species directly deposit and anchor on carbon substrate, achieving the controllable and precise synthesis of ADMSEs. Li et al.^[^ ^66^ ^]^ used the ALD strategy to obtain the Co single atom catalysts by controlling the cycle number, since more cycle numbers tended to cause the formation of nanoparticle. Noteworthily, the presence of Pt single atoms could effectively lower the dissociation energy of Co(Cp)_2_, and ensure the formation of the isolated Co sites. Moreover, this synthesis strategy was also extended to the synthesis of Fe and Ni single atom catalysts, and expanded the application range of monatomic catalysis. The isolated Pt sites were anchored on the pyridinic N‐doped carbon substrate by ALD.^[^ ^69a^ ^]^ The Pt‐N coordination could be observed, and abundant electron vacancy was formed from XAS result. The synergistic effect between the Pt sites and N‐doped sites of the porous carbon substrates could efficiently modify the electronic structure, enhancing the catalytic activity. Similarly, the atomically dispersed Co sites were deposited around the Pt nanoparticles by ALD, and Pt catalysts were modified with isolated Co atoms for ORR and HER. The atomic‐resolution HAADF‐STEM and XAS characterizations were conducted to prove the presence of isolated Co atoms around Pt, and all experimental and theoretical results indicated there is strong electronic synergy between Co and Pt.^[^ ^130^ ^]^

## 5 Effective Strategies for Rational Design of Atomically Dispersed Metal Site Electrocatalysts

**Figure 11:**
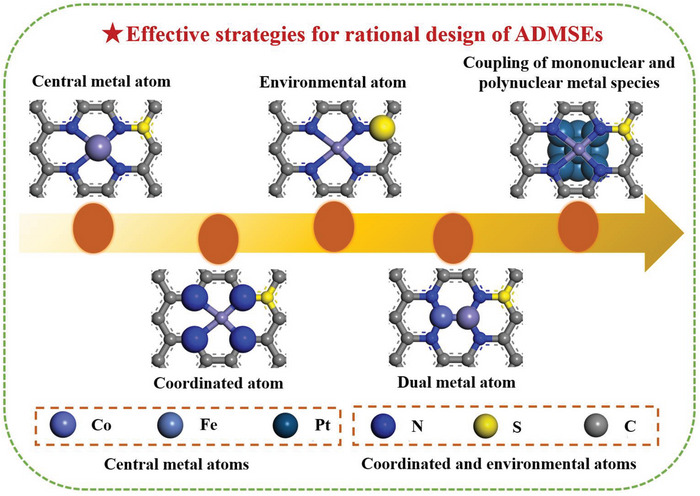
The schematic illustration of strategies for rational design of ADMSEs.

Generally, active sites dominate the whole catalytic reaction, and the effective strategies of selectively tailoring active sites to regulate the intrinsic activity are crucial for the rational design of the advanced ADMSEs.^[^ ^2^, ^134^ ^]^ The electronic structure is closely related to the catalytic performance, and the high‐efficiency electrocatalysts tend to rely on the efficient electronic effects.^[^ ^134^ ^]^ During the electrocatalysis of oxygen, the d orbitals of metal atoms generally interact with p orbitals of oxygen‐containing intermediates to generate new orbitals, which endows them with unprecedented electrocatalytic activity due to the optimal electronic structure.^[^ ^16^, ^37^, ^135^ ^]^ To obtain the high‐efficiency ORR electrocatalyst, tremendous efforts have been made to the rational design of ADMSEs. In this section, the effective strategies for the rational design of ADMSEs are summarized into five parts (**Figure** **11**): i) Central metal atoms: The central metal atoms are generally considered as the active sites and interact with oxygen‐containing intermediates, thus selecting suitable metal atoms can directly maximize the catalytic activity; ii) Coordinated atoms: The electronegativity differences between the central metal atoms and the coordinated atoms generally trigger a redistribution of electrons, and the strong electronic interaction can effectively modulate the intrinsic catalytic activity; iii) Environmental atoms: The presence of environmental atoms can further affect the electronic structure and activate the central metal sites, adjusting the catalytic activity; iv) Dual metal atoms: The dual metal sites can generate a stronger d orbital hybridization between the two adjacent metal atoms, and tend to elongate the O—O bond and reduce the bond cleavage barrier; v) Coupling of mononuclear and polynuclear metal species: Single metal sites modified by polynuclear metal species can optimize the electronic structure, and the composite sites can efficiently facilitate the activation of the O—O bond, reducing the energy barrier of O—O bond cleavage. **Table** **1** provides a summary of ADMSEs based on various design strategies for ORR.

**Table 1:** Summary of ORR catalytic performance of ADMSEs based on various design strategies

Electrocatalyst	Active center	*E*_1/2_ [V] versus RHE	Cycling stability	Electrolyte	Ref.
Central metal site strategy					
Fe‐NC SAC	Fe‐N_4_	0.9	Negligible degradation after 5000 cycles	0.1 m KOH	[17a]
GO‐Fe‐N	Fe‐N_4_	0.87	–	0.1 m KOH	[19a]
Fe_1_‐HNC‐500‐850	Fe‐N_4_	0.842	Negligible decay after 20 000 s	0.1 m KOH	[109]
Fe‐N‐Grs	Fe‐N_4_	0.87	Current retention of 95.6% after 40 000 s	0.1 m KOH	[136]
Co‐CMS	Co‐N_4_	0.83	No obvious voltage fading after 594 h	0.1 m KOH	[6c]
Zn‐B/N‐C/	N_2_‐Zn‐B_2_	0.886	Current density retention of 97% after 36 000 s	0.1 m KOH	[26b]
Mn‐N‐C	MnN_4_C_12_	0.8	17 mV loss after 30 000 cycles	0.5 m H_2_SO_4_	[113]
SnNC	Sn(IV)N*_x_*	0.73	–	0.1 m HClO_4_	[137]
Coordinated atom strategy					
Fe/N/C	Fe‐N_4_O	0.825	10 mV loss after 5000 cycles	0.1 m HClO_4_	[138b]
CuSA@HNCN_x_	Cu‐N*_x_*	0.91	No obvious decay after 5000 cycles	0.1 m KOH	[58]
SW‐N‐C	W‐N_5_	0.88	Mass activity loss of 13.9% after 10 000 cycles	0.1 m KOH	[139]
FeN_4_	Pyrrole‐type Fe‐N_4_	0.8	26 mV loss after 10 000 cycles	0.5 m H_2_SO_4_	[44]
SA‐Fe/NG	Fe‐pyrrolic‐N‐C_8_	0.8	12 mV loss after 5000 cycles	0.5 m H_2_SO_4_	[140]
Fe‐N‐C	Edge‐hosted Fe‐N_4_	0.915	Negligible decay after 10 000 cycles	0.1 m KOH	[50]
Fe‐N/P‐C	Fe‐N_3_P	0.867	Negligible decay after 40 h	0.1 m KOH	[49]
Co‐N,B‐CSs	Co‐N_3_B	0.83	Almost no decay after 5000 cycles	0.1 m KOH	[141]
Cu‐SA/NPSC	Cu−N_3_S_1_	0.84	Negligible degeneration after 10 000 cycles	0.1 m KOH	[142]
S‐Cu‐ISA/SNC	Cu‐S_1_N_3_	0.918	Current retention of 98% after 100 h	0.1 m KOH	[16a]
FeCl1N4/CNS	Fe‐Cl_1_‐N_4_	0.921	8 mV loss after 10 000 cycles	0.1 m KOH	[143]
FeNC	Fe‐N_4_	0.804	Current retention of 98.7% after 10 000 s	0.5 m H_2_SO_4_	[68]
Fe‐N/C	Fe‐N_2_	0.735	–	0.5 m H_2_SO_4_	[144]
Environmental atom strategy					
Ru‐SAS/SNC	RuN_4_‐S	0.861	24 mV loss after 30 000 cycles	0.1 m KOH	[2a]
CoNOC	O‐adjacent C in CoNOC	‐	Faradaic efficiency of ≈95% after 11 h	0.1 m HClO_4_	[48]
FeNC‐S‐MSUFC‐2,	Fe‐N_4_	0.73	Kinetic activity of 19% loss after 3000 cycles	0.5 m H_2_SO_4_	[16b]
Fe‐ISA/SNC	Fe‐N_4_‐S_2_	0.896	Almost no decay after 15 000 cycles	0.1 m KOH	[126]
S‐FeNC‐900	S‐FeNC	0.76	17 mV loss after 10 000 cycles	0.1 m HClO_4_	[145]
Fe‐SAs/NPS‐HC	Fe‐ N_4_PS	0.912	No obvious decay after 5000 cycles	0.1 m KOH	[146]
FeBNC	B doped Fe‐N_4_	0.968	No obvious decay after 40 000 s	0.1 m KOH	[147]
Dual metal site strategy					
Fe‐N_4_/Pt‐N_4_@NC	Fe‐N_4_/Pt‐N_4_ moieties	0.93	8 mV loss after 10 000 cycles	0.1 m KOH	[15b]
A‐CoPt‐NC	a(Co‐Pt)@N_8_V_4_	0.96	Mass activity of 96.4% after 8000 cycles	0.1 m KOH	[70b]
Zn/CoN‐C	ZnCo‐N_6_	0.861	9 mV loss after 10 000 cycles	0.1 m KOH	[21]
Pt_1_@Fe‐N‐C	Pt_1_‐O_2_‐Fe_1_‐N_4_	0.8	12 mV loss after 10 000 cycles	0.5 m H_2_SO_4_	[110]
PtFeNC	Pt‐N*_x_*/Fe‐N*_x_*	0.895	13 mV loss after 5000 cycles	0.1 m KOH	[55]
(Fe,Co)/CNT)	Fe‐N_3_/Co‐N_3_	0.954	Minimal degradation after 10 000 cycles	0.1 m KOH	[148c]
Zn,Co‐N*_x_*‐C‐Sy	ZnCo‐N*_x_*‐S	0.893	4.4% current loss after 20 000 s test	0.1 m KOH	[149]
CoNi‐SAs/NC	Co‐Ni‐N*_x_*	0.76	Activity retention of 90% after 16 h	0.1 m KOH	[53]
CoPNi‐N/C	Co‐N_4_/Ni‐N_4_	0.84	–	0.1 m KOH	[65a]
Co‐N‐C‐10	Co_2_‐N_5_	0.79	–	0.1 m HClO_4_	[56]
Fe_2_‐N‐C	Fe_2_‐N_6_	0.78	20 mV loss after 20 000 cycles	0.5 m H_2_SO_4_	[150a]
Planar‐like Fe_2_N_6_	Fe_2_‐N_6_	0.84	–	0.5 m H_2_SO_4_	[150b]
FeCo‐IA/NC	Fe‐N_4_/Co‐N_4_	0.88	Current retention of 96.4% after 20 000 s	0.1 m KOH	[151]
FeCo_2_‐NPC‐900	FeN*_x_* and CoN*_x_*	0.87	Negligible difference after 10 000 cycles	0.1 m KOH	[148a]
(Fe,Co)/N‐C	FeCo‐N_6_	0863	Negligible decay after 50 000 cycles	0.1 m HClO_4_	[148b]
(Fe,Co)/CNT	FeCo‐N_6_	0.954	Negligible decay after 10 000 cycles	0.1 m KOH	[148c]
FeCo‐ISAs/CN	FeN_4_ and CoN_4_	0.92	Almost no decay after 5000 cycles	0.1 m KOH	[148d]
CoFe@C	FeN*_x_* and CoN*_x_*	0.89	Current retentions of 88% after 20 000 s	0.1 m KOH	[148e]
Fe, Mn‐N/C	Fe‐N_4_ and Mn‐N_4_	0.904	21 mV loss after 10 000cycles	0.1 m KOH	[152a]
Mn‐Fe‐N/S@mC	Fe‐N_4_ and Mn‐N_2_S_2_	0.896	Current retention of 95% after 12 h	0.1 m KOH	[152b]
FeNi‐N6	Fe‐N_3_/Ni‐N_3_	‐	12 mV loss after 5000 cycles	0.1 m HClO_4_	[153]
Fe‐NiNC‐50	Fe‐N_3_/Ni‐N_3_	0.75	9 mV loss after 5000 cycles	0.1 m KOH	[54]
Co/Zn‐NCNF	N_2_‐Co‐N_2_‐Zn‐N_2_	0.797	12 mV loss after 10 000 cycles	0.1 m HClO_4_	[52]
Cu/Zn‐NC	Cu‐N_4_ and Zn‐N_4_	0.83	Almost unchanged after 10 000 cycles	0.1 m KOH	[70a]
Coupling mononuclear and polynuclear species strategy					
FeNC‐S‐Fe*_x_* C/Fe	Fe‐N*_x_*	0.821	Not decay after 10 000 cycles	0.1 m HClO_4_	[154]
Co_NP_/Co_SA_‐N‐C	Deformed Co‐N_4_	0.83	12 mV loss after 50 000 cycles	0.1 m HClO_4_	[62]
Fe@C‐FeNC	Fe‐N*_x_*	0.899	16 mv loss after 5000 cycles	0.1 m KOH	[155]
FeAC@FeSA−N−C	Fe‐N_4_	0.912	Current density retention of 91.8% after 30 000 s	0.1 m KOH	[156]
Pt_A_@Fe_SA_‐N‐C/	Fe‐N_4_	0.923	7 mv loss after 5000 cycles	0.1 m HClO_4_	[157a]
Fe‐N‐HMCTs	Fe‐N*_x_* and Fe_3_C GL NPs	0.872	12 mv loss after 5000 cycles	0.1 m KOH	[157b]
PtCuCo@Co‐N‐C	PtCuCo and CoN_4_	0.95	19 mv loss after 50 000 cycles	0.1 m HClO_4_	[158]
H‐PtCo@Pt_1_N‐C	Pt_3_Co and Pt_1_N‐C	‐	Only a 4.5% loss of diffusion‐limited current density after 10 000 cycles	0.1 m HClO_4_	[159]

### 5.1 Central Metal Site Strategy

Central metal atoms are generally considered as the active sites for the electrocatalysis of oxygen, and changing the types of central metal atoms types is a relatively straightforward and simple approach to the rational design of the advanced ADMSEs.^[^ ^17^, ^137^ ^]^ The selection of central metal atoms is mainly concentrated within noble metal and TM atoms, including Pt, Co, Ni, Fe, Mn, which can be achieved by changing the different metal precursors during the catalyst synthesis.^[^ ^17^, ^136^ ^]^ Different metal atoms can endow ORR with different catalytic activity because of different band structures and electronic effects. Typically, the interaction between the d orbitals of central metal atoms and 2p orbitals of O atoms or intermediates can produce the antibonding state. Shifting the antibonding orbitals can optimize the adsorption energy of RDS reaction, affecting the adsorption strength between central metal atoms and oxygen‐containing intermediates.^[^ ^16^, ^37^ ^]^ And the reaction process will trigger the electron transfer and modulate the electronic structure, realizing the modification of electrocatalytic activity.^[^ ^37^, ^160^ ^]^ Additionally, the valence electrons of the occupied d orbitals of central metal atoms are in close association with the binding energies of the intermediates. As for this strategy, it is difficult to high‐efficiency ADMSEs only through experiments due to unavoidable experimental errors and huge workload, and selecting the proper central metal atoms to construct efficient ADMSEs for ORR still remains challenging. The effective combination of theoretical simulation and experiment is of vital importance to obtain high‐efficiency ORR electrocatalysts.^[^ ^109^, ^136^ ^]^ The theoretical simulation method can predict and compare the catalytic activity of different central metal active sites before the experiments, and provide a valuable guideline for rational design of the high‐efficiency ADMSEs.

**Figure 12:**
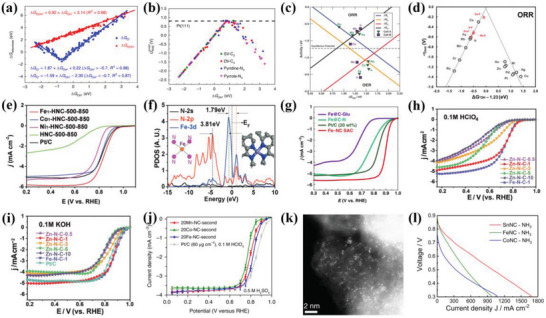
a) The relationship of Δ*G*_OOH*_ and Δ*G*_O*_ versus Δ*G*_OH*_ for all TM single atom catalysts supported on graphene. b) Theoretical onset potential toward ORR versus Δ*G*_OH*_ for all TM single atom catalysts supported on graphene. Reproduced with permission.^[^ ^162^ ^]^ Copyright 2018, Springer Nature. c) Combined volcano plot relationship of activity versus Δ*G*_OH_ for the ORR (up) and OER (below). Reproduced with permission.^[^ ^161a^ ^]^ Copyright 2011, The Royal Society of Chemistry. d) The volcano plot relationship of theoretical overpotential for the ORR versus Δ*G*_OH*_−1.23 eV. Reproduced with permission.^[^ ^161b^ ^]^ Copyright 2017, The Royal Society of Chemistry. e) LSV curves of the as‐prepared M_1_‐HNC‐500‐850 (Fe, Co, and Ni), HNC‐500‐850, and commercial Pt/C in 0.1 m KOH solution. Reproduced with permission.^[^ ^109^ ^]^ Copyright 2020, Wiley‐VCH. f) PDOSs of Fe‐N_4_ local motif structure. Reproduced with permission.^[^ ^109^ ^]^ Copyright 2020, Wiley‐VCH. g) ORR polarization curves of the as‐prepared catalysts and commercial Pt/C. Reproduced with permission.^[^ ^17a^ ^]^ Copyright 2019, Springer Nature. h) Polarization curves of Zn‐N‐C‐*x* (*x* = 0.5, 1, 3, 5, and 10), Fe‐N‐C‐1 and Pt/C electrocatalysts for ORR in O_2_‐saturated 0.1 mm HClO_4_ medium at 1600 rpm. i) Polarization curves of Zn‐N‐C‐0.5, Zn‐N‐C‐1, Zn‐N‐C‐3, Zn‐N‐C‐5, Zn‐N‐C‐10, Fe‐N‐C‐1, and Pt/C electrocatalysts for ORR in O_2_‐saturated 0.1 m KOH medium at a scan rate of 10 mV s^−1^ and 1600 rpm. Reproduced with permission.^[^ ^127^ ^]^ Copyright 2019, Wiley‐VCH. j) ORR polarization curves of M‐N‐C electrocatalysts (M = Fe, Co, and Mn) synthesized from identical procedures. Reproduced with permission.^[^ ^113^ ^]^ Copyright 2018, Springer Nature. k) HAADF‐STEM image of isolated Sn atoms supported in or on graphene layers. l) Fuel cell polarization curves for MNC (M = Sn, Fe, and Co) after NH_3_ activation. The cathode and anode loadings were 4 and 2 mg_Pt_ cm^−2^, respectively. Cell conditions: 100% relative humidity (RH) at H_2_‐O_2_ atmosphere, Tanode = Tcathode = 80 °C at 2 bar absolute pressure. Polarization curves were obtained with a linear sweep voltammetry of 1 mV s^−1^. Reproduced with permission.^[^ ^137^ ^]^ Copyright 2020, Springer Nature.

At present, huge efforts have been made to reveal the role of central metal sites on electrocatalytic reactions, and understand the activity trend of different central metal sites and intrinsic ORR mechanisms.^[^ ^161^ ^]^ The general design principle for assessing the activity of graphene‐based single atom electrocatalysts for ORR was established based on various catalyst models.^[^ ^162^ ^]^ According to the calculated Δ*G*, there were different linear relationships for Δ*G*_OOH*_ and Δ*G*_O*_ related to Δ*G*_OH*_ on the total single atom catalyst system (**Figure** **12**a). The Δ*G*_OOH*_ versus Δ*G*_OH*_ could be correlated and fitted into a good linear relationship. However, the theoretical Δ*G*_O*_ toward Δ*G*_OH*_ showed the piecewise relationships at different Δ*G*_OH*_ ranges. Additionally, the universal volcano relationships between *U*_onset_ and Δ*G*_OH*_ could be obtained for single TM atoms on graphene (Figure 12b). Fe and Co single atom electrocatalysts were generally at the top of the theoretical volcano, especially indicating the great potential of Fe coordinated with pyridine and pyrrole‐N_4_ for ORR. In addition, the macrocyclic metal compounds were also used as alternatives to graphene‐based single atom catalysts. The TM of groups 7 and 9 supported on graphitic materials were also proved to be more active for ORR by constructing the catalyst models of 13 kinds.^[^ ^161a^ ^]^ Among these TM, Fe, Ir, Mn, Ru, and Rh metal sites had more proper adsorption strength with intermediates, and the high‐efficiency catalysts could effectively catalyze oxygen molecules, leading to the lowest ORR overpotentials (Figure 12c). Moreover, different metals were chemically modulated on the metalloporphyrin frameworks, which could effectively regulate the electronic properties.^[^ ^161b^ ^]^ There was a theoretical volcano activity relationship between the overpotential of ORR and the Δ*G* of intermediates (Figure 12d). Noteworthily, the catalysts from group 9 were closest to the top of the volcano curve, and thus considered as the most potential electrocatalysts for ORR owing to the lower overpotential.

Inspired by the theoretical simulation, many experimental studies have been conducted to verify such ORR activity trends.^[^ ^17^, ^19^, ^20^ ^]^ A series of single atom electrocatalysts anchored on N‐doped porous carbon (TM_1_‐HNC) followed the ORR activity sequence of Fe_1_‐HNC‐500‐850 > Co_1_‐HNC‐500‐850 > Ni_1_‐HNC‐500‐850 (Figure 12e).^[^ ^109^ ^]^ The ORR activity differences between these different metal sites mainly stemmed from the different electronic effects. In particular, the strong d‐p orbital coupling between the central metal sites and intermediates could result in spatial charge separation, and endow Fe‐N_4_ with the optimal electron state (Figure 12f). The induced charge transfer effectively overcame the Coulomb barrier, improving the d‐band activity of FeN_4_ catalysts. Besides, Fe‐N_4_ moiety dominated the charge transfer pathway by adjusting the electronic structure, stabilizing the disorder state and regulating the surface electronic activity of carbon support. Similar conclusion was also drawn from Meng’ group.^[^ ^136^ ^]^ ZnO nanoparticles as template precursors were used to achieve the even dispersion of isolated metal sites on graphene. They had demonstrated such catalytic activity trend of Fe > Co > Cu > Ni through both experimental and theoretical simulation. Noteworthily, the superior ORR activity for Fe single atom catalysts was mainly ascribed to the synergistic effect of the reasonable band structure, abundant active sites and robust support. Among these monodispersed metal atom catalysts with ultrahigh loading (Fe, Mn, Co, Ni, Cu, Mo, Pt, etc.) obtained from the cascade anchoring strategy, Fe catalysts with the high metal loading of 8.9% exhibited the best ORR performance than other electrocatalysts (Figure 12g).^[^ ^17a^ ^]^ It was also worth noting that despite the superior ORR activity of single‐atom Fe catalyst, the associated Fenton reaction generally caused poor electrochemical stability, and lowered the energy efficiency during the electrocatalysis process.

Currently, Fe‐free single metal atom catalysts have been widely applied for ORR due to their better catalytic performance.^[^ ^6^, ^18^, ^26^ ^]^ Zn single atom catalyst (Zn‐N‐C) with ultrahigh Zn loading of 9.33 wt% was proved for efficient ORR electrocatalysis.^[^ ^127^ ^]^ Other than the comparable ORR activity than Fe‐N‐C catalysts, Zn‐N‐C catalysts had better electrochemical stability in both acidic and alkaline mediums. In acidic medium, Zn‐N‐C catalysts exhibited a more positive *E*_1/2_ of 0.746 V than that of the Fe‐N‐C catalyst (0.743 V) (Figure 12h,i). Similarly, the same activity trend was also obtained in alkaline medium. It was remarkable that the Zn‐N‐C catalysts showed the ultralow yield of H_2_O_2_ and followed the efficient four‐electron ORR pathway, indicating the outstanding catalytic efficiency for ORR. Moreover, the anticorrosion ability of these single atom catalysts was closely related to the binding strength between the OH^*^ species and isolated metal sites. The transfer of Zn^*^ sites into Zn(OH)^*^ (1.42 eV) tended to require more free energy than that of Fe^*^ sites (1.07 eV), suggesting the better anticorrosion ability for Zn‐N‐C catalysts. Mn single sites on partially graphitic carbon (Mn‐N‐C) catalyst was also considered as the potential ORR catalyst, especially the high‐density Mn‐N‐C catalyst with N coordination which showed more impressive catalytic performance for ORR.^[^ ^113^ ^]^ Mn‐N‐C catalysts had similar *E*_1/2_ and even better electrochemical stability than Fe‐N‐C catalysts in acidic solution (Figure 12j). The high‐density MnN_4_ sites endowed the Mn‐N‐C catalysts with higher activity, and the enhanced stability was mainly attributed to the synergistic effect of the robust MnN_4_ sites and the support with high corrosion resistance. Additionally, Mn‐N‐C catalysts tended to follow the four‐electron reduction pathway by overcoming the energy barrier to cleave the O—O bonds. Similarly, a p‐block Sn single atom catalyst (SnNC catalyst) with a Fenton‐inactive character also followed the four‐electron ORR pathway in acidic medium (Figure 12k).^[^ ^137^ ^]^ The SnNC catalysts could transfer metallic or oxidic Sn species into active sites for achieving the efficient oxygen reduction, showing higher current density than FeNC catalysts (Figure 12l). Notably, the intrinsic active moieties of Sn‐N sites with low pyridinic N coordination endowed the SnNC catalysts with appropriate oxygen chemisorption energy, improving catalytic efficiency for ORR.

### 5.2 Coordinated Atom Strategy

Although the strategy of changing central metal atom types can efficiently regulate ORR performance, the limited metal types usually restrict the range of adjustment to some extent. Apart from readily accessible central metal atoms, regulating the local coordination environment is also a facile route for the rational design of ADMSEs, since the regulation for nonmetallic heteroatoms directly coordinated with central metal atoms can significantly modify the ORR performance for ADMSEs.^[^ ^134^, ^160^ ^]^ According to the Sabatier principle, the interaction between the catalysts and the reactants cannot be too strong or too weak, and appropriate bonding strength can achieve the maximization of catalytic activity.^[^ ^135^ ^]^ As an effective strategy for regulating catalytic activity, the local coordination environment generally determines the binding strength between the central metal atoms and the adsorbed intermediates.^[^ ^49^, ^134^ ^]^ Moreover, regulating the local coordination environments of central metal sites can effectively adjust the electronic structure and change the electronic filling state of d orbital, resulting in the production of new catalytic active sites for ADMSEs.^[^ ^134^ ^]^ Additionally, the strong anchoring effect between the central metal atoms and the coordination atoms can ensure the monodispersity of metal atoms and enhance the stabilization of ADMSEs.^[^ ^58^, ^160^ ^]^ Revealing the relationship between the local coordination environment of ADMSEs and catalytic activity facilitates a better understanding of the real ORR mechanisms, providing a valuable guideline for the rational design of ADMSEs. In general, there are two ways to tailor the local coordination environments for the rational design of ADMSEs, including the coordination atom types and numbers around the central metal atoms.

#### 5.2.1 Modulation of Coordination Atom Type

Electrocatalytic reaction mainly involves the adsorption and desorption between the central metal sites and the intermediates.^[^ ^2a^ ^]^ The electronic interaction between the metal atoms and the coordination atoms can optimize the adsorption free energy of electrocatalysts, improving the ORR performance.^[^ ^28^, ^134^ ^]^ In addition to the conventional N and C atoms, other heteroatoms such as S, P, and B are also introduced to regulate the local coordination environment of ADMSEs, endowing central metal atoms with unique electronic structures.^[^ ^16^, ^49^, ^141^ ^]^ Different coordination atom types generally induce different electronic effects, affecting the intrinsic ORR activity of ADMSEs. Selectively manipulating the coordinated atom types is an efficient way to enhance ORR performance for ADMSEs.^[^ ^138^ ^]^

**Figure 13:**
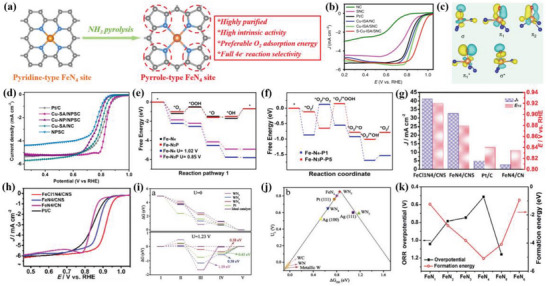
a) Schematic depiction of synthetic process for high‐purity pyrrole‐type FeN_4_ structure. The balls in grey, blue and orange represent C, N, and Fe atoms, respectively. Reproduced with permission.^[^ ^44^ ^]^ Copyright 2020, The Royal Society of Chemistry. b) LSV curves of S‐Cu‐ISA/SNC and other references for ORR. c) Molecular orbitals of O^*^ adsorbed on Cu‐S_1_N_3_ in S‐Cu‐ISA/SNC. *σ* and *σ* ^*^ represent the bonding and antibonding between d_z_^2^ O, *π* ^2^ represents the bonding between d_x_^2^_‐y_^2^ orbital of Cu and p orbital of O, *π*_1_, and *π*_1_^*^ represent the bonding and antibonding between d_yz_/d_xz_ orbital of Cu and p orbital of orbital of Cu and p orbital of O. Reproduced with permission.^[^ ^16a^ ^]^ Copyright 2020, Springer Nature. d) LSV curves of Cu‐SA/NPSC and other references at a rotating rate of 1600 rpm for ORR. Reproduced with permission.^[^ ^142^ ^]^ Copyright 2019, American Chemical Society. e) The change trend for Gibbs free energy of Fe‐N_4_ and Fe‐N_3_P active sites under different potentials during ORR. f) Gibbs free energy diagrams for the optimized Fe‐N_4_‐P_1_ and Fe‐N_3_P‐P_5_ active sites during ORR under alkaline conditions. Reproduced with permission.^[^ ^49^ ^]^ Copyright 2020, American Chemical Society. g) Comparison of kinetic current density at 0.85 V and *E*_1/2_ for different catalysts in KOH medium. h) ORR polarization curves of FeCl_1_N_4_/CNS and other references obtained from 0.1 m KOH solution. Reproduced with permission.^[^ ^143^ ^]^ Copyright 2018, The Royal Society of Chemistry. i) Gibbs free energy diagrams of WN_3_, WN_4_, WN_5_, Pt, and ideal catalyst for ORR under different U (0 and 1.23 V vs RHE), with the RDS highlighted and the values of the limiting energy barrier labelled. i): O_2_(g) + 4(H_2_O + e^−^); ii): OOH^*^ + OH^−^ +3(H_2_O + e^−^); iii): O^*^ + 2OH^−^ + 2(H_2_O + e^−^); iv): OH^*^ + 3OH^−^ + H_2_O + e^−^; v): 2H_2_O(l) + 4OH^−^. j) The volcano plot relationship of *U*_L_ versus Δ*G*_OH_. The Δ*G*_OH_ values of W, WN, and WC catalysts were −1.05, −0.50, and −0.46 eV, respectively. Reproduced with permission.^[^ ^139^ ^]^ Copyright 2019, Elsevier. k) Formation energies and ORR overpotentials of different FeN*_x_* (*x* = 1–6) active sites. Reproduced with permission.^[^ ^68^ ^]^ Copyright 2019, The Royal Society of Chemistry.

As a conventional active site configuration, M‐N*_x_* sites are generally formed by the coordination of central metal atoms and N atoms in the carbon support, and have been proved to be active for ORR.^[^ ^134^, ^160^ ^]^ In particular, different types of N atoms can lead to different local electronic structures of the central metal atoms, affecting the catalytic activity and selectivity of the catalysts due to the optimization of adsorption free energy between the central metal atoms and adsorbates.^[^ ^71^ ^]^ Generally, N atoms in M‐N*_x_* sites are classified into pyridinic, pyrrolic, graphitic, and oxidized N, especially the N coordinated with the central metal atoms, which are usually pyridinic‐N and pyrrolic‐N.^[^ ^58^, ^139^ ^]^ For different types of pyrrole and pyridine N, FeN_4_ sites coordinated with high‐purity pyrrole‐type N possessed more efficient catalytic performance for ORR (**Figure** **13**a).^[^ ^44^ ^]^ The pyrrole‐type FeN_4_ sites had stronger electron loss than that of pyridine‐type FeN_4_ sites, but the electron loss around the adjacent pyrrole N was weaker, indicating the significant charge distribution difference between pyrrole‐type and pyridine‐type FeN_4_ sites. It was especially positive for the valence state of the pyrrole‐type Fe than the pyridine‐type Fe. The pyrrole‐type FeN_4_ had a lower Δ*G* between O_2_ and OOH^*^ and more sufficient 4e^−^ reaction path selectivity, indicating the stronger oxygen adsorption to ORR. In addition, pyrrole‐type FeN_4_ sites also showed a low thermodynamic overpotential from the initial state (O_2_) to the reaction pathway. Similarly, the surfactant‐assisted method was applied to obtain SA‐Fe/NG catalysts, and showed more outstanding stability for ORR in both acidic and alkaline mediums.^[^ ^140^ ^]^ The high ORR activity of Fe single atom catalysts SA‐Fe/NG) mainly stemmed from the Fe‐pyrro‐N species with pyrrole N, since this molecular incorporation easily triggered an order of magnitude increase in the active sites, revealing the puzzle between few Fe sites and high ORR activity. Noteworthily, the low p‐band center of the carbon in Fe@pyridinic‐N made it difficult to adsorb the reaction intermediates, and diffuse toward the Fe in Fe@pyridinic‐N, but was conducive to be adsorbed on the C of Pyrrolic‐N. The pyrrolic‐type N could promote the synergistic interaction between MN_4_ sites and surrounding C atoms, and was more active for ORR than pyridine‐type MN_4_. Inspired by the experimental results, Lin et al.^[^ ^71^ ^]^ also revealed the intrinsic ORR mechanism by theoretical calculation. The prediction resulting from machine learning showed that the Fe@pyrrole‐N_2_C_2_ catalysts were more active than the Fe@pyridine‐N_1_C_3_ catalysts. Moreover, the N atoms located in edge could efficiently modify the adsorption strength between FeN_4_ sites and reaction intermediates, leading to better catalytic activity.^[^ ^50^ ^]^ Additionally, N atom doping can also effectively improve the interaction between metal atoms and carbon substrates via the formation of M‐N bonds.^[^ ^79^, ^163^ ^]^

In addition to N atoms, the local electronic structure of central metal atoms for ADMSEs can also be regulated by coordinating other non‐metallic heteroatoms such as S, P, and B, and theses non‐metallic heteroatoms efficiently modulated the d band center of ADMSEs due to the different electronegativity and atomic radius.^[^ ^49^, ^141^, ^142^ ^]^ Because electronegativity difference significantly causes the electron transfer between the central metal atoms and the surrounding non‐metallic heteroatoms to some extent, the valence electrons from the d orbital of the metal atoms are changed, which contributes to the synergistic interaction between active sites and reaction intermediates. Unlike the conventional CuN_4_ sites, Cu single atom catalysts with S and N coordination (S‐Cu‐ISA/SNC) catalysts with unsymmetrically arranged Cu‐S_1_N_3_ site derived from the porous carbon exhibited more excellent catalytic activity for ORR (Figure 13b).^[^ ^16a^ ^]^ Owing to the electronegativity difference caused by S, more electrons occupied the D_x_^2^_‐y_^2^ orbitals of the Cu atoms in the Cu‐S_1_N_3_ catalysts. Moreover, the *π* bonds originated from the P orbitals of oxygen and the Cu_dyz_, D_XZ_, and D_x_^2^_‐y_^2^ orbitals were different from the Cu‐N_4_, and the additional *π* bonds contributed by Cu D_x_^2^_‐y_^2^ orbital significantly strengthened the bonds between Cu sites and the intermediates, thus improving the ORR performance of Cu‐S_1_N_3_ catalysts (Figure 13c). The presence of S could effectively contribute to the synergistic interaction between the Cu metal sites and carbon substrates, and activated the electronic interaction of Cu‐N_3_S_1_ sites and the oxygenated species, which were active for ORR (Figure 13d).^[^ ^142^ ^]^ Moreover, P atoms also had positive influence on ORR by tailoring the local electronic structure of central metal sites. The N‐P double coordination could efficiently promote ORR performance by regulating the electronic structure of Fe sites.^[^ ^49^ ^]^ Compared with traditional Fe‐N_4_ sites, O_2_ could be easily adsorbed and firmly bounded to the Fe‐N_3_P sites, showing more outstanding ORR activity than that of Fe‐N_4_ (Figure 13e). Both experiment and theoretical calculations proved that Fe‐N_3_P sites could further optimize the adsorption and desorption process of oxygen‐containing intermediates, and accelerate the kinetics of oxygen reduction. In addition, the Fe‐N_3_P‐P_5_ also showed a lower energy barrier than that of Fe‐N_4_‐P_1_, indicating that the introduction of P could effectively reduce the energy barrier (Figure 13f). The Co single atom catalysts with B atom coordination (Co‐N,B‐CSs) were proved to be more active to oxygen electrocatalysis than the traditional Co‐N sites.^[^ ^141^ ^]^ The theoretical results showed that the addition of B atoms could efficiently activate Co‐N sites and contribute to the synergistic interaction between the Co‐N,B sites and oxygenated species. Fe single atom catalysts with Cl and N coordination (FeCl_1_N_4_/CNS) obtained via a thermal‐migrating method could also regulate the local electronic structure of the central metal atom by Cl coordination.^[^ ^143^ ^]^ The FeCl_1_N_4_/CNS catalysts presented a higher *E*_1/2_ of 0.921 V and kinetic current density of 41.11 mA cm^−2^ at 0.85 V than FeN_4_/CNS, FeN_4_/CN and Pt/C (Figure 13g,h). The enhanced ORR performance could be ascribed to the optimal electronic structure of the Fe sites from the strong interaction between Fe and Cl.

#### 5.2.2 Modulation of Coordination Atom Number

In addition to the coordination atom types, changing coordination atom numbers is also an effective strategy for constructing advanced ADMSEs.^[^ ^68^, ^164^ ^]^ The appropriate adjustment for the coordination atom numbers can endow ADMSEs with a reasonable electronic structure, optimize the Gibbs free energy and promote the synergistic interaction between the central metal sites and the oxygenated intermediates.^[^ ^139^ ^]^ WN*_x_* catalysts with different W‐N coordination numbers (*x* = 1, 3, 4, and 5) made a high‐efficiency catalytic performance for ORR.^[^ ^139^ ^]^ The W‐N coordination numbers were precisely regulated by changing relevant synthesis parameters, and WN_5_ catalysts exhibited better ORR performance than the WN_3_ and WN_4_ catalysts in both acidic and alkaline electrolytes. Noteworthily, regulating the W‐N coordination numbers made significant contribution to the synergistic interaction between the OH^−^ and central W sites. The RDS for WN_5_ catalysts required only a smaller barrier or overpotential (0.38 eV) than that of WN_3_ and WN_4_ catalysts, which was highly closed to the conventional Pt catalysts (0.45 eV) (Figure 13i). Especially for W, WN, and WC samples, they were located in the lower left corner of the volcano diagram, suggesting the strong binding with OH^*^, and are not adaptable to ORR. However, the WN_5_ was located at the top of the volcano diagram, indicating the most appropriate binding energy between the W sites and OH^*^ (Figure 13j). Similarly, the catalytic activity of FeN*_x_* catalysts (*x* = 1, 3, 4) followed the order of FeN_4_ > FeN_3_ > FeN_1_, which proved that regulating Fe‐N coordination numbers could efficiently modify ORR electrocatalytic activity.^[^ ^68^ ^]^ Remarkably, the FeN*_x_* catalysts followed a “volcano” relationship, and the FeN_4_ located at the top of the “volcano” diagram had the lowest formation energy (Figure 13k). In addition, different coordination numbers ca cause different adsorption models between the metal sites and intermediates. O atoms tended to occupy Fe‐C bridge sites of the FeN_1_ and FeN_3_ sites, O atoms, however, were usually adsorbed to top sites of Fe sites for FeN_4_ and FeN_5_, triggering different electronic properties for FeN*_x_* catalysts. The five‐coordinated Fe‐N sites could also lead to a lower ORR energy barrier and adsorption energy between the Fe sites and OH intermediates than the Fe‐N/C electrocatalysts with two Fe‐N coordination, demonstrating the crucial role of different coordination numbers for ORR electrocatalysis.^[^ ^144^ ^]^ What's more, many studies have also proved that the coordination number has a close relationship with the central electronic structure and the catalytic performance.^[^ ^164^, ^165^ ^]^

### 5.3 Environmental Atom Strategy

**Figure 14:**
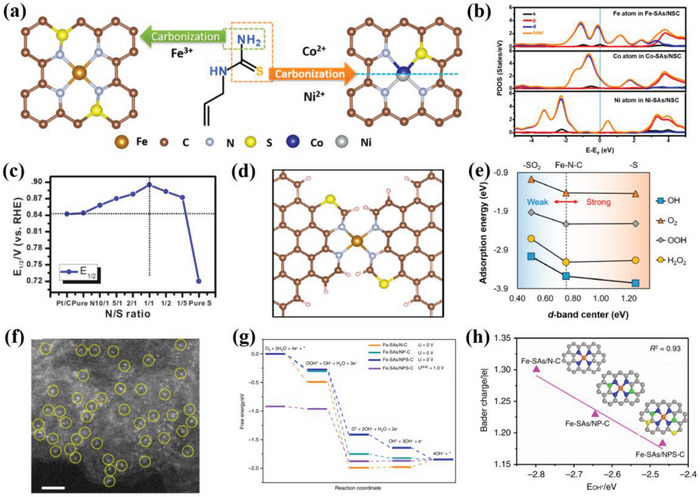
a) The Schematic depiction of synthetic process of M‐SAs/NSC (M = Fe, Co, and Ni) with different coordination environments. b) The calculated partial DOS of different metal atoms in M‐SAs/NSC (M = Fe, Co, and Ni) with the aligned Fermi level. Reproduced with permission.^[^ ^17b^ ^]^ Copyright 2019, American Chemical Society. c) The linear relationship between *E*_1/2_ and Fe‐ISA/SNC catalysts with different N/S ratios. Reproduced with permission.^[^ ^126^ ^]^ Copyright 2018, Springer Nature. d) The optimized structure of Fe‐N_4_‐C catalyst with the functional groups (yellow spheres). The spheres with dark brown, light blue, and light brown spheres for C, N, and Fe atoms, respectively. e) The linear relationship of the adsorption energy for different intermediates versus the d‐band center. Reproduced with permission.^[^ ^16b^ ^]^ Copyright 2019, American Chemical Society. f) The AC‐HAADF‐STEM image of Fe‐SAs/NPS‐HC electrocatalyst. g) ORR Gibbs free energy diagram of Fe‐SAs/N‐C, Fe‐SAs/NP‐C, and Fe‐SAs/NPS‐C under different *U*_RHE_ (*U*_RHE_ = 0 and 1 V). h) The linear relationship of Bader charge versus OH^*^ binding energy for isolated Fe atoms in Fe‐SAs/N‐C, Fe‐SAs/NP‐C, and Fe‐SAs/NPS‐C, respectively. The balls from model with orange, blue, green, yellow, and gray for Fe, N, P, S, and C atoms, respectively. Reproduced with permission.^[^ ^146^ ^]^ Copyright 2018, Springer Nature.

Unlike the coordinated atoms mentioned above, environmental atoms are indirectly connected to the central metal atoms by coordination atoms, and are generally located at the farther coordination shell, such as the second and third coordination shells.^[^ ^2^, ^48^ ^]^ Compared with the coordinated atoms, environmental atoms have a greater range of regulating catalytic performance due to the presence of more coordination shells. As another effective strategy for the rational design of ADMSEs, environmental atoms can effectively activate the central metal sites, modify the electronic structure of the central metal site, lower energy barrier of catalytic reaction and promote the interaction between the metal sites and the adsorbed intermediates for ADMSEs.^[^ ^16^, ^126^ ^]^ S, as the environmental atom, was used to activate Fe single atom catalysts anchored on porous N, S codoped carbon substrates (Fe‐SAs/NSC), and directly coordinated with Fe central metal site by N (**Figure** **14**a).^[^ ^17b^ ^]^ Unlike Fe‐SAs/NSC with FeN_4_S_2_ sites, metal‐S bonds were found for Co‐SAS/NSC catalysts with CoN_3_S_1_ sites and Ni‐SAS/NSC catalysts with NiN_3_S_1_ sites due to different metal precursors. The outstanding ORR performance mainly stemmed from higher charge density and lower energy barrier for the FeN_4_S_2_ catalysts. Compared with Co (0.09) of CoN_3_S_1_ and Ni (0.03) of NiN_3_S_1_, Fe center sites in FeN_4_S_2_ had a higher charge density of 0.11. In addition, Fe showed the highest occupied states nearing the Fermi level mainly because the Fe d orbitals greatly promoted the DOS of M‐SAs/NSC. (Figure 14b). Similarly, there was a “volcano” diagram relationship between the ratio of N/S and the *E*_1/2_ of the Fe‐ISA/SNC electrocatalysts with S as environmental atom (Figure 14c).^[^ ^126^ ^]^ One‐step strategy was used to synthesize S‐doped Fe single site catalysts by in situ introducing S, and these catalysts exhibited enhanced *E*_1/2_ in both acid and alkaline mediums.^[^ ^145^ ^]^ Environmental atoms can also contribute to the interaction between support substrates and single metal sites. Mun et al.^[^ ^16b^ ^]^ modulated the electronic transfer property of carbon substrate and modified the catalytic reaction kinetic of Fe‐N_4_ sites by introducing S atoms (Figure 14d). The oxidized sulfur lowered the d‐band center of single Fe atoms, and increased the adsorption strength of the intermediates, promoting the ORR electrocatalytic activity (Figure 14e). P and S co‐doping as the environmental atoms on porous carbon substrates could also regulate the electronic properties of central Fe sites (Fe‐SAs/NPS‐C) (Figure 14f).^[^ ^146^ ^]^ The introduction of S and P atoms triggered the long‐distance interaction between Fe central sites and heteroatoms, and modulated the electronic structure of central Fe sites, improving the reaction kinetics for ORR in acid and alkaline mediums. The desorption of OH intermediates was the RDS for Fe‐SAs/N‐C and Fe‐SAs/NP‐C from the free energy diagram, while Fe‐SAs/NPS‐C exhibited stronger reactivity and higher reduction kinetics than Fe‐SAs/N‐C and Fe‐SAs/NP‐C (Figure 14 g). Environmental atoms significantly contributed to the electron transfer from the S and P to the Fe central sites, promoting the Bader charge and weakening the binding strength of Fe‐SAs/NPS‐C with OH^−^ (Figure 14 h). In addition, B as the environmental atom was also introduced to activate the conventional FeN_4_ sites in porous carbon, and the incorporation of B further improved the overall ORR performance.^[^ ^147^ ^]^

### 5.4 Dual Metal Site Strategy

Apart from single metal site catalysts, dual metal site catalysts have also been proved to be highly active for ORR electrocatalysis.^[^ ^15^, ^16^ ^]^ Generally, single metal atoms coordinated with heteroatoms can modify the catalytic activity of ADMSEs, but cannot increase the active sites for the electrocatalytic reaction. Unlike single metal site catalysts, the d orbitals of two adjacent metal sites usually hybridize during the reaction, which reduces the energy barrier of key reaction steps, cleaves the O—O bond, and modulates the electronic structure,^[^ ^16^, ^70^ ^]^ which is distinct to the p‐d orbital hybridization effect of single metal sites and the surrounding heteroatoms.^[^ ^21^, ^166^ ^]^ Moreover, dual metal sites tend to dissociate the O_2_ into OOH^*^ and OH^*^ by the lower energy barrier, endowing ADMSEs with the high‐efficiency ORR. Inspired by this, huge efforts have been made for atomically dispersed dual metal site catalysts, which has become an attractive direction for the rational design of advanced ORR electrocatalysts for researchers.^[^ ^16^, ^55^, ^151^ ^]^

**Figure 15:**
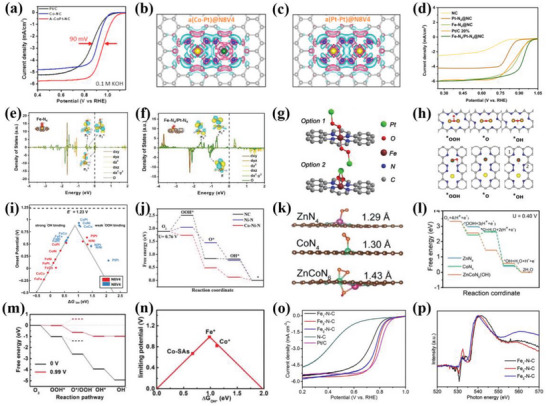
a) LSV of the as‐prepared samples and Pt/C catalyst in 0.1 m KOH solution. b) Top view of the charge densities of a(Co‐Pt)@N_8_V_4_. c) Top view of the charge densities of a(Pt‐Pt)@N_8_V_4_. Reproduced with permission.^[^ ^70b^ ^]^ Copyright 2019, American Chemical Society. d) ORR polarization curves of various electrocatalysts in a KOH solution with a scan rate of 5 mV s^−1^. e) The calculated PDOS of Fe‐N_4_ catalyst with OOH^*^ intermediate adsorption. f) The calculated PDOS of Fe‐N_4_/Pt‐N_4_ catalyst with OOH^*^ intermediate adsorption. Reproduced with permission.^[^ ^15b^ ^]^ Copyright 2021, Wiley‐VCH. g) The schematic models for Pt_1_@FeNC catalyst with Pt_1_‐O_2_‐Fe_1_‐N_4_‐C_12_ as the active moiety. Reproduced with permission.^[^ ^110^ ^]^ Copyright 2017, Wiley‐VCH. h) The model structures of the binding mode between the N_6_V_4_ and N_8_V_4_ and the intermediates (OOH^*^, O^*^, and OH^*^) for ORR. i) ORR activity volcano plot between the onset potential and Δ*G*_OH_ for the pores N_6_V_4_ and N_8_V_4_ labeled by red and blue, respectively. Reproduced with permission.^[^ ^167^ ^]^ Copyright 2019, American Chemical Society. j) Gibbs free energy diagram for NC, Ni‐N, and Co‐Ni‐N electrocatalysts during ORR. Reproduced with permission.^[^ ^53^ ^]^ Copyright 2019, Wiley‐VCH. k) Optimized geometry of ZnN_4_, CoN_4_, and ZnCoN_6_(OH) with O_2_ adsorption. Brown, blue, yellow, green, purple, and red balls represented C, N, O, Zn, Co, and H atoms, respectively. l) Gibbs free energy diagram for ZnN_4_, CoN_4_, and ZnCoN_6_(OH) during the ORR under acidic solution at *U* = 0.40 V. Reproduced with permission.^[^ ^21^ ^]^ Copyright 2019, Wiley‐VCH. m) Free energy diagram of (Fe,Co)/CNT with OH^*^ on Fe site for ORR at 0 and 0.99 V. The dashed lines represented the 2e^−^ ORR pathway. C, N, Fe, Co, O, and H atoms are presented by gray, blue, orange, green, red, and white spheres, respectively. n) Volcano plot relationship of ORR activity versus Δ*G*_OH*_. Fe^*^, Co^*^, and Co‐SAs from Fe and Co sites of the OH^*^ anchored (Fe,Co)/CNT and Co sites of Co SAs/N‐C, respectively. Reproduced with permission.^[^ ^148c^ ^]^ Copyright 2018, The Royal Society of Chemistry. o) Polarization curves of Fe*_x_*‐N‐C (*X* = 1, 2, and 3), N/C and Pt/C for ORR in 0.5 m H_2_SO_4_ medium at a scan rate of 10 mV s^−1^ and 1600 rpm. p) O K‐edge NEXAFS spectrum of Fe*_x_*‐N‐C (*X* = 1, 2, and 3) catalysts. Reproduced with permission.^[^ ^150a^ ^]^ Copyright 2019, Elsevier.

Atomically dispersed dual metal site catalysts based on precious metals have been extensively studied.^[^ ^55^, ^110^, ^167^ ^]^ Dual site catalysts with Co‐Pt coupling coordination (A‐CoPt‐NC) possessed outstanding catalytic performance for ORR.^[^ ^70b^ ^]^ A‐CoPt‐NC catalysts had higher *E*_1/2_ and mass activity than that of the commercial Pt/C catalysts (**Figure** **15**a). Moreover, A‐CoPt‐NC showed a higher selectivity for 4e^−^ reaction pathway for ORR, and lower H_2_O_2_ production and O—O dissociation energy. Noteworthily, the d‐2p orbital hybridization between dual Pt‐Co metal sites and oxygen led to the formation of the new orbitals during the catalytic reaction, and the energy of d orbital for the a(Co‐Pt)@N_8_V_4_ (N_8_ and N_4_ represent the number of N and vacant atoms, respectively) was closer to Fermi level compared with a(Pt‐Pt)@N_8_V_4_, indicating the stronger adsorption ability to oxygen. Additionally, the strong electronic interactions from the asymmetric distribution of Co‐Pt dual sites significantly triggered the transfer and accumulation of more electrons around Co atoms of a(Co‐Pt)@N_8_V_4_ than the a(Pt‐Pt)@N_8_V_4_ (Figure 15b,c). Fe‐N_4_/Pt‐N_4_@NC electrocatalyst with isolated Fe‐N_4_ and Pt‐N_4_ dual metal sites encapsulated on N‐doped carbon substrates exhibited an extremely high *E*_1/2_ of 0.93 V versus RHE for ORR in KOH solution (Figure 15d).^[^ ^15b^ ^]^ The presence of Pt‐N_4_ sites efficiently facilitated the activation of O_2_ molecules adsorbed on the Fe‐N_4_ sites, and triggered the rehybridization between the d orbitals of Fe and the p orbitals of O, which optimized the ORR kinetic process. Compared to single Fe‐N_4_ sites with weaker Fe‐OOH binding ability (Figure 15e), the neighboring Pt‐N_4_ sites significantly modulated the d orbital energy level and spatial distribution of Fe‐N_4_ sites, and the reconstruction of spatial charge distribution gave rise to the change of the net charge of Fe atom in Fe‐N_4_/Pt‐N_4_@NC from +0.541 to +0.537 (Figure 15f). Similarly, many works about Pt‐Fe dual metal sites have also been developed for ORR electrocatalysis.^[^ ^55^, ^110^ ^]^ PtFe dual site electrocatalysts were constructed by encapsulating Pt sites into Fe‐doped ZIF, showing an ultrahigh *E*_1/2_ of 0.895 V versus RHE and a specific capacity of 807 mAh g^−1^ at 10 mA cm^−2^ for Zn–air batteries. Compared with the Pt and Fe single sites, the Pt‐Fe dual metal sites had a smaller Δ*G*, and energetically tended to cleave the O—O bonds.^[^ ^55^ ^]^ Unlike the conventional dual metal sites, a new active moiety of Pt_1_‐O_2_‐Fe_1_‐N_4_ was also engineered by connecting the Pt and Fe sites with oxygen molecules (Figure 15 g). This moiety effectively protected the Fe site from foreign harsh environment damage and strengthened the proton adsorption and O_2_ reduction ability.^[^ ^110^ ^]^ Encouraged by these experiment results about the dual metal site catalysts, Hunter et al.^[^ ^167^ ^]^ also explored the catalytic activity of single and dual‐atom catalysts composed of different TM atoms such as Pt, Co, Fe, and Ni for ORR via DFT method. It was worth noting that different transition atoms dispersed on graphene with different N coordination numbers generally caused different adsorption modes between the intermediates and metal sites. The reaction intermediates tended to be adsorbed on a bridge site between the dual metal sites for the CoPt@N_6_V_4_ (N_8_ and N_4_ represent the number of N and vacant atoms, respectively). The reaction intermediates were, however, stably adsorbed on the Co sites of CoPt@N_8_V_4_ with a single bond (Figure 15h). Moreover, the CoPt@N_8_V_4_ catalysts with Pt‐Co dual sites exhibited lower overpotential (0.30 V) than that of Co‐Ni (0.35 V) and Co‐Co (0.37 V) dual sites, and the optimal Δ*G*_OH_ for ORR (Figure 15i).

Apart from the noble metals, many dual metal sites composed of TM have also been explored, such as FeCo,^[^ ^26^, ^148^, ^151^ ^]^ FeMn,^[^ ^152^ ^]^ FeNi,^[^ ^54^, ^153^ ^]^ CoNi,^[^ ^53^, ^65^ ^]^ ZnCo,^[^ ^21^, ^52^, ^149^ ^]^ ZnCu.^[^ ^168^ ^]^ Dual site electrocatalysts by encapsulating the connected Fe‐Co dual metal sites on N‐doped carbon nanotubes ((Fe,Co)/CNT) possessed a superior *E*_1/2_ of 0.954 V and an energy density of 260 mW cm^−2^ than Pt/C in Zn–air batteries.^[^ ^169^ ^]^ The enhanced catalytic activity mainly originated from the activation of oxygen by lowering the dissociation energy of O—O bonds, Fe‐Co dual metal sites could effectively restrict the 2e^−^ reaction pathway, and had high selectivity for the 4e^−^ reaction pathway (Figure 15j). In addition, the Fe sites of (Fe,Co)/CNT showed the maximum theoretical limiting potential from the ORR volcano plot (Figure 15k). The Zn/CoN‐C electrocatalyst with isolated Zn‐Co dual site also revealed a superior *E*_1/2_ of 0.861 and 0.796 V in alkaline and acid mediums for ORR, respectively.^[^ ^21^ ^]^ Compared with isolated ZnN_4_ (1.29 Å) and CoN_4_ (1.3 Å) sites, Zn‐Co dual metal sites tended to cleave O—O bonds energetically by elongating the bond length of O—O from 1.23 to 1.42 Å (Figure 15l). Noteworthily, the reduction of OH^*^ intermediates was a spontaneous process, and was inclined to form ZnCoN_6_‐(OH) structure with Zn‐Co sites, generating a more stable ORR process. Moreover, the overall reaction was exothermic, and all elementary reaction steps were downhill in both alkaline and acid mediums from the free energy diagram, indicating that the whole ORR process was more likely to occur on ZnCoN_6_(OH) (Figure 15m). The catalysts by confining the Zn‐Co dual sites in dentric carbon (Zn,Co‐N*_x_*‐C‐S*_y_*) were also proved to be beneficial to the activation and cleavage of O—O bonds.^[^ ^149^ ^]^ In particular, the introduction of S heteroatom could further optimize the electronic structure around Zn and Co sites, enhancing the synergistic effect between the active sites and reaction intermediates. CoNi‐SAs/NC electrocatalysts with Co‐Ni dual metal sites on N‐doped hollow carbon nanocubes could efficiently optimize the adsorption and desorption of reaction intermediates, and lower the reaction barrier for ORR process.^[^ ^53^ ^]^ Compared with NC (1.09 eV) and Ni‐N (0.63 eV), the Co‐Ni dual metal sites energetically improved the ORR reaction kinetic, and decreased the limiting barrier for the hydrogenation of O_2_ to 0.35 eV. Additionally, the first reaction steps were elevated for NC and Ni‐N, while all reaction steps for Co‐Ni dual metal sites became downhill, demonstrating the crucial role of the dual site for the superior ORR activity (Figure 15n). Similarly, Co‐Ni dual metal sites on porous carbon frameworks are also proved to greatly contribute to the electronic transfer, and optimize the electronic properties for ADMSEs.^[^ ^65a^ ^]^

Apart from different TM atoms, homologous dual‐metal site catalysts have also been developed.^[^ ^56^, ^150^ ^]^ Fe*_x_* site catalysts (*x* = 1, 2, and 3) on N‐doped carbon (Fe*_x_*‐N‐C) derived from the pore of the MOF were constructed for ORR.^[^ ^150a^ ^]^ This precise tunability of Fe atom numbers facilitated the changing of O_2_ adsorption from superoxide to peroxide configuration, and enabled the adjustment of N species in C matrix during pyrolysis process. Fe_2_‐N‐C exhibited more excellent ORR activity in acidic medium than Fe_1_‐N‐C and Fe_3_‐N‐C, with a higher *E*_1/2_ of 0.78 V versus RHE and an outstanding durability of only −20 mV shift after 20 000 cycles (Figure 15o). Compared with Fe1‐N‐C (1.34Å), the adding of Fe atoms significantly promoted the elongation of O—O bonds (1.48 Å for Fe_2_‐N‐C and 1.49 Å for Fe_3_‐N‐C). Fe_2_‐N‐C especially exhibited higher transition identity at 532 eV from O K‐edge NEXAFS result than Fe_1_‐N‐C and Fe_3_‐N‐C due to the electronic transfer and activation of O_2_ (Figure 15p). Similarly, the planar‐like Fe_2_N_6_ dual sites catalysts from the thermal migration led to the formation of dual‐side adsorption by connecting O atoms, facilitating the dissociation of O—O bonds. However, the isolated FeN_4_ sites only showed end‐on adsorption, which usually caused a high energy barrier of O—O bond dissociation.^[^ ^150b^ ^]^ The Co_2_N_5_ dual metal sites were also constructed, and the ORR catalytic activity followed the order of Co_2_N_5_ > CoN_4_ > Co@C, which was mainly ascribed to the lack of thermodynamic barrier around the Co_2_N_5_(OH) sites. In addition, the pre‐absorbed OH on Co_2_N_5_ could further improve the catalytic reaction.^[^ ^56^ ^]^

### 5.5 Coupling Strategy of Mononuclear and Polynuclear Metal Species

Apart from the single and dual metal site electrocatalysts, coupling the mononuclear and polynuclear metal species is considered as an emerging efficient strategy for rational design of the advanced ORR electrocatalysts.^[^ ^62^, ^154^ ^]^ Unlike the electrocatalysts composed of only isolated metal atoms, this unique structural advantage can greatly facilitate the synergistic effect between mononuclear and polynuclear metal species by virtue of the presence of composite sites.^[^ ^62^ ^]^ During the electrocatalysis, mononuclear metal species are generally considered as active sites, and polynuclear metal species mononuclear can provide conductive channels for the electronic transfer.^[^ ^155^ ^]^ Constructing the composite sites can efficiently facilitate the activation of O—O bonds, reduce the energy barrier of O—O bond cleavage and optimize the electronic properties of ADMSEs. Moreover, the synergistic effect between the mononuclear and polynuclear metal species can break the limitation of the proportional relationship between the electrocatalytic reactions, especially improving the anti‐corrosion capabilities of catalysts due to the encapsulation of carbon layer around the polynuclear metal species.^[^ ^170^ ^]^

**Figure 16:**
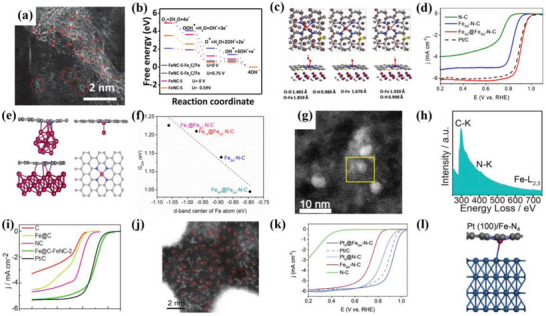
a) The HAADF‐STEM image of FeNC‐S‐Fe*_x_* C/Fe catalysts. b) Gibbs free energy diagram of FeNC‐S‐Fe*_x_* C/Fe and FeNC‐S models for ORR in an alkaline condition. c) The relaxed atomic structure of the FeNC‐S‐Fe*_x_* C/Fe model obtained by DFT calculations. The brown, yellow, blue, gray, and white spheres represent Fe, S, N, C, and H atoms, respectively.^[^ ^154^ ^]^ Copyright 2018, Wiley‐VCH. d) Polarization curves of the as‐synthesized samples and Pt/C in O_2_‐saturated 0.1 m KOH condition with a rotation rate of 1600 rpm and a scan rate of 10 mV s^−1^. e) The calculated structure models of Fe_1_@Fe_SA_‐N‐C, Fe_13_@Fe_SA_‐N‐C, Fe_NP_@Fe_SA_‐N‐C, and Fe_SA_‐N‐C. f) The linear relationship between the d‐band center and Δ*G*_OH*_ for Fe‐N‐C electrocatalyst systems. Reproduced with permission.^[^ ^156^ ^]^ Copyright 2019, American Chemical Society. g) HAADF‐STEM image of Fe@C‐FeNC‐2 electrocatalyst. h) EELS spectrum of Fe@C‐FeNC‐2 electrocatalyst. i) LSV curves of different electrocatalysts for ORR with steady state. Reproduced with permission.^[^ ^155^ ^]^ Copyright 2016, American Chemical Society. j) HAADF‐STEM image of Pt_A_@Fe_SA_‐N‐C electrocatalyst. k) Polarization curves of various electrocatalysts with in O_2_‐saturated 0.1 m HClO_4_ condition at 1600 rpm. l) Computational models of Pt(100)/Fe‐N_4_, Fe‐N_4_, and Pt(100) obtained DFT. Reproduced with permission.^[^ ^157a^ ^]^ Copyright 2020, The Royal Society of Chemistry.

In recent years, the coupling of single metal sites and nanoclusters has been widely reported.^[^ ^154^, ^156^ ^]^ Qiao et al.^[^ ^154^ ^]^ used a synchronous sulfurization method to construct a Fe‐N‐C electrocatalyst containing Fe*_x_* C/Fe species (FeNC‐S‐Fe*_x_* C/Fe) for ORR electrocatalysis (**Figure** **16**a). The heterogeneous Fe*_x_* C and Fe subatomic clusters, which were sulfurated to form Fe—S bonds greatly improved the utilization rate of Fe atoms, and maximized the ORR activity. The relatively large atomic radius of S led to defects in the carbon support, and the low electronegativity of S could change the electronic structure of the Fe‐N active centers. The EXANFS, XANES, XPS, and Mössbauer spectroscopy proved the presence of FeN_4_ single sites around Fe*_x_* C. DFT results showed that the enhanced ORR activity for FeNC‐S‐Fe*_x_* C/Fe resulted from the synergistic effect of Fe‐N active center, Fe*_x_* C/Fe micro‐clusters and S‐containing species (Figure 16b,c). Similarly, the electrocatalyst with isolated Fe sites and Fe atomic clusters derived from covalent organic framework (Fe_AC_@Fe_SA_‐N‐C) possessed a higher *E*_1/2_ of 0.912 V versus RHE than that of commercial Pt/C (0.897 V), Fe_SA_‐N‐C (0.844 V), and most of the non‐Pt group metal catalysts (Figure 16d).^[^ ^156^ ^]^ Noteworthily, the enhanced ORR activity mainly originated from the Fe‐N_4_ active centers, and the coupling of Fe atomic clusters further activated and endowed Fe‐N_4_ sites with outstanding catalytic activity. In addition, the synergetic effect between isolated Fe sites and Fe atomic clusters significantly led to the drop of d‐band center and the decrease of OH binding energy (Figure 16e,f).

In addition, huge efforts have been put into the coupling of single metal atoms and nanoparticles.^[^ ^155^, ^157^ ^]^ Coupling Fe‐N*_x_* single sites and Fe/Fe_3_C nanocrystals (Fe@C‐FeNC) can effectively enhance the catalytic performance for ORR.^[^ ^155^ ^]^ The unique site structure with the Fe‐N*_x_* sites around Fe/Fe_3_C nanocrystals was successfully proved by a series of characterizations (Figure 16g,h). The Fe@C‐FeNC catalysts showed the highest *E*_1/2_ of 0.899 V than that of the N‐doped carbon support, Fe/Fe_3_C nanocrystals and Fe‐N*_x_*, implying that the presence of Fe/Fe_3_C nanocrystals better activated Fe‐N*_x_* sites (Figure 16i). Remarkably, Fe‐N*_x_* sites dominated the overall catalytic activity of catalysts, and the blocking of Fe‐N*_x_* sites greatly degraded the overall activity of Fe@C‐FeNC catalyst. Moreover, the synergistic effect between the Fe‐N*_x_* sites and Fe/Fe_3_C nanocrystals contributed to the electronic transfer from the additional Fe atoms to N atoms and reduced the Mulliken charges of central Fe atoms, optimizing the adsorption energy of O_2_. The strong coupling effect between metal nanoparticles (M‐NPs) and atomically dispersed M‐N_4_ sites was also revealed.^[^ ^171^ ^]^ Stable M‐NPs doping at Fe sites was achieved by controllable doping of FeCo in the Zn‐ZIF precursor. The synthesized catalysts containing the composite site of M‐NPs and MN_4_ showed higher ORR activity. The strong interaction between M‐NP and FeN_4_ effectively activated the O—O bond after O_2_ adsorption. The special side‐on adsorption for O_2_ on Fe sites of M/FeN_4_ could extend the corresponding O—O length to 1.422 Å, facilitating the cleavage of O—O bond and high selectivity of 4e^−^ process. CoNi nanoparticles as “solid‐ligands” could also activate Co‐N*_x_*‐C active sites, effectively regulating the electronic structure and accelerating the oxygen catalytic kinetics.^[^ ^172^ ^]^ It was worth noting that the electronic coupling effect caused by CoNi alloy nanoparticles as a solid ligands also weakened the adsorption strength of OH^*^ on the active center of Co‐N*_x_*‐C, thus favoring the desorption of OH^*^ intermediates. Moreover, the coupling effect effectively promoted electronic transfer from d‐orbital electrons of Co‐N*_x_*‐C to CoNi atoms of nanoparticles, reducing the d‐band center in Co‐N*_x_*‐C. Similarly, encapsulating the ordered Pt‐alloy core‐shell nanoparticles on carbon substrates with Fe‐N‐C sites (Pt_A_@Fe_SA_‐N‐C) could greatly optimize the binding strength between metal sites and intermediates (Figure 16j).^[^ ^157a^ ^]^ The Pt_A_@Fe_SA_‐N‐C catalysts exhibited ultrahigh *E*_1/2_ of 0.923 V versus RHE and power density of 1.31 W cm^−2^ (Figure 16k), and possessed a higher onset potential than that of Pt(100) and Fe‐N_4_, which was mainly ascribed to the synergistic effect between the Pt alloy and Fe‐N_4_ site (Figure 16l). Noteworthily, the different electronegativity from the Fe atoms to Pt atoms further facilitated the electronic transfer and resulted in the weaker binding strength between the OH^*^ and single Fe sites. Co nanoparticles and Co‐N_4_ electrocatalyst (CoNP‐CoN_4_) with CoNP‐CoN_4_ composite sites was also favorable to facilitate the activation of oxygen molecules, and presented more positive *E*_1/2_ of 0.83 V versus RHE and outstanding durability with 12 mV loss after 50 000 cycles.^[^ ^62^ ^]^ Moreover, the unique composite sites could further inhibit the yield of H_2_O_2_ and optimize the adsorption of radical oxygen species, enhancing the activity and durability of catalysts. Likewise, atomically dispersed Fe sites and graphitic layer‐wrapped Fe_3_C nanoparticles (Fe_3_C@GL NPs) simultaneously encapsulated on 1D N‐doped hollow carbon tubes also possessed higher ORR activity, and the enhanced catalytic performance was certified from the unique active site configurations of Fe‐N*_x_* sites incorporated Fe_3_C@GL NPs.^[^ ^157b^ ^]^ Huang et al.^[^ ^158^ ^]^ designed an efficient electrocatalyst containing the ternary PtCuCo alloy and isolated graphitic Co‐N‐C site (PtCuCo@Co‐N‐C). The result also demonstrated that the synergistic effect between the porous Pt‐based alloy and Co‐N‐C site efficiently improved the catalytic property. The isolated Pt sites around the PtCo nanoparticles could modify the catalytic and the novel Pt_1_N‐C shells effectively protected hollow Pt_3_Co from agglomeration.^[^ ^159^ ^]^ In addition, such unique structure of coupling the atomically dispersed metal sites and nanoparticles has also been applied in other catalytic reactions.^[^ ^173^ ^]^

## 6 Key Issues and Applications of Atomically Dispersed Metal Site Electrocatalysts for Electrochemical Energy Devices

Developing efficient, stable and low‐cost ADMSEs is of great significance for the development of electrochemical energy devices. Generally, there are many performance differences between half cells and full cells. For instance, half cells only achieve good catalytic performance at low current density due to insufficient oxygen solubility in acid solution, and cannot predict reliable information from full cells according to the electrochemical system from half cells. Therefore, it is highly crucial that electrocatalysts are evaluated in full cells for practical application of ADMSEs.^[^ ^174^ ^]^ In spite of various advantages, the practical application of ADMSEs still faces great challenges, such as the low active site density and insufficient stability. In the following section, we will first discuss the present challenges to be addressed in practical application, and then summarize the application of ADMSEs in fuel cells and Zn–air batteries.

### 6.1 Key Issues to be Addressed for Practical Application

Generally, active sites are inextricably linked to the catalytic performance, and the number of accessible active site densities should be directly proportional to the electrocatalytic ORR performance measured in electrochemical energy devices.^[^ ^15^, ^133^ ^]^ Highly catalytic active sites can effectively lower the RDS of catalytic reactions and optimize the binding strength between the active site and oxygen‐containing intermediates, enhancing the catalytic efficiency for ADMSEs.^[^ ^36^, ^37^ ^]^ Low site density and insufficient stability are still the crucial hindrances to the practical application of ADMSEs.

#### 6.1.1 Active Site Density Issue for Atomically Dispersed Metal Site Electrocatalysts

For the active site density, increasing the metal site loading and achieving high accessibility of active sites for ADMSEs are of great importance for the practical application of ADMSEs.^[^ ^129^, ^175^ ^]^ This goal currently faces two main challenges: 1) The simultaneous formation of inactive or low active metal species and monodispersed metal sites during pyrolysis; 2) The low utilization rate for sites buried in the N‐doped carbon matrix.^[^ ^15a^ ^]^ To overcome the above challenges, more efforts have been devoted to the regulation of active site density, and much progress has been made for ADMSEs.^[^ ^175^, ^176^ ^]^

##### Increasing Metal Site Loading

The active site density is of great importance for catalytic performance for ORR, since the metal loading is usually proportional to the number of active sites.^[^ ^124^, ^176^, ^177^ ^]^ The template method has been widely used to prepare ADMSEs with high metal loading.^[^ ^175^, ^176^ ^]^ SiO_2_ template was first dispersed in Co source solution before the addition of other precursors, and hollow N‐doped carbon spheres were obtained by template‐assisted pyrolysis and subsequent etching treatment.^[^ ^176^ ^]^ Noteworthily, the Co metal atom loading reached 2.2 wt%, and this strategy effectively improved the exposure of Co active sites and the mass transfer efficiency of ORR‐relevant species and generation of OH^*^ intermediates. The optimal Co single site catalysts exhibited higher *E*_1/2_ of 0.773 V in acidic solution, and negligible change in the polarization curve was observed after 10 000 cycles. Similarly, Fe sites anchored on industrial Vulcan XC‐72 by a Si‐assisted strategy also could achieve the similar result.^[^ ^175^ ^]^ The introduction of the Si template not only facilitated the formation of active Fe‐N_4_ sites, but also prevented the generation of inactive metal species of Fe, Fe*_x_* C, or Fe*_x_* O*_y_* during the synthesis. In addition, the facile transition behavior of the Fe(II) in out plane to Fe(III) in plane endowed Fe‐N‐C catalysts with better ORR performance than that of commercial Pt/C in alkaline solution. Though the template method can increase metal atom loading, the additional synthetic complexity of removing the template usually causes a lot of uncertainty for the preparation of ADMSEs, which is inconducive to the large‐scale production of ADMSEs.^[^ ^175^, ^178^ ^]^ Zn coordination sites of high density as sacrificial metal were used to achieve the high loading of Fe sites for PEMFCs.^[^ ^178b^ ^]^ The Zn‐NC was first obtained from the high‐temperature pyrolysis under 900 °C, and the catalysts were finally obtained by the pyrolysis of Fe^2+^, Zn‐NC, and dicyandiamide (DCDA) under mixed H_2_/N_2_ atmosphere (Fe‐NC^Δ‐DCDA^). Both HAADF‐STEM and EDS proved the uniform distribution of Fe sites in the carbon matrix. Noteworthily, the content of isolated Fe atoms reached 7.10 wt%, which was determined by ICP‐MS and the measured site density reached 4.7–7.8 × 10^19^ sites g^−1^ for Fe‐NC^Δ‐DCDA^. PEMFCs with Fe‐NC^Δ‐DCDA^ catalyst exhibited a higher current density of 41.3 and 145 mA cm^−2^ for H_2_‐O_2_ system at 0.90 V_IR‐free_ and H_2_‐air system at 0.80 V at 0.80 V_IR‐free_, respectively. Pluronic F127 block copolymer as surfactant is also applied to obtain the isolated Co single atom catalysts by carbon‐shell confinement strategy for fuel cell.^[^ ^178a^ ^]^ It was remarkable that the unique confinement effect effectively inhibited the aggregation of Co sites and maintained the integrity of the pore structure for ZIF‐8. The presence of the surfactant F127 significantly increased the metal loading of Co atoms, and the corresponding number of active sites also doubled. The optimal Co single site catalysts with F127 assist exhibited higher *E*_1/2_ of 0.84 V and power density of 0.87 W cm^−2^ for half cells and H_2_‐O_2_ fuel cells, respectively. The enhanced catalytic could be attributed to the presence of substantial CoN_2+2_ sites. The synthesis strategies generally involve the high‐temperature pyrolysis process for ADMSEs. Since metal atoms easily agglomerate into nanoclusters or nanoparticles due to high surface energy, acid leaching is generally applied to remove additional metal species. However, this strategy still remains a great challenge for developing the advanced ADMSEs with high loading of metal atoms.^[^ ^124^, ^177^ ^]^ Jiang et al.^[^ ^179^ ^]^ developed an efficient plasma engineering strategy to obtain Fe/Co diatomic catalysts with high metal loading on porous nitrogen‐doped carbon nanofibers (Fe, Co SAS‐PNCF). The Fe/Co diatomic catalyst precursor was first synthesized via electrospinning, and Fe, Co SAs‐PNCF catalysts were obtained by N_2_ plasma treat without any acid leaching. Noteworthily, plasma engineering could produce a large number of defects in N‐doped carbon nanofibers, providing the possibility to achieve high metal loading, and the loading of the Fe/Co double sites reached 9.8 wt%. The catalyst exhibited excellent ORR performance in both alkaline and acidic medium. The confinement strategy was also used to obtain high‐loading Fe single site catalysts, and the isolated Fe atom loading reached 3.5 wt% by the molecules‐confined pyrolysis strategy.^[^ ^180^ ^]^ The formation of metal particles or clusters was restricted by one‐step pyrolytic reduction with the addition of phenanthroline organic small molecule isolator, achieving the synthesis of Fe monatomic catalyst without further etching and pyrolysis. The synthesized Fe monoatomic catalysts had good ORR performance in PEMFCs and Zn–air batteries. The maximum power density and mass capacity of Zn–air batteries reached 225 mW cm^−2^ and 636 mAh g^−1^, respectively, and the peak power density reached 0.35 W cm^−2^ for PEMFCs under H_2_/air. A sequential coordination method was proposed to develop single atom catalysts by doping a large number of metal atoms (Ir, Rh, Pt, Pd) into ZIF, and then pyrolyzed into N‐coordinated single atom catalysts (M_1_‐N/C) with a metal load of up to 1.2–4.5 wt%.^[^ ^41b^ ^]^

In addition, while regulating site density, more attention should be paid to the effect of site distance on catalytic performance.^[^ ^6^, ^181^ ^]^ It was proved that the inter‐site distance (*D*_site_) and catalytic performance using a universal strategy of anchoring single atoms in hydrogel precursors had a close relationship.^[^ ^6b^ ^]^ The isolated Fe sites exhibited enhanced ORR kinetic rate with the decrease of *D*_site_, and the strong interaction between sites effectively regulated the electronic structure, improving the intrinsic ORR performance. In particular, when the *D*_site_ between Fe sites was greater than 1.2 nm, the mass current density had no obvious dependence on *D*_site_. As the *D*_site_ decreased, the mass activity significantly increased. However, the intrinsic catalytic activity did not continue to increase when it was lower than 0.7 nm. This related work well emphasized the existence and importance of synergistic effects between metal sites. Similarly, Han et al.^[^ ^181^ ^]^ also revealed the relationship between the intersite distance and ORR performance. Limiting potential was associated with the neighboring effects of two adjacent Fe sites, and the regulation of intersite distance could effectively optimize the nature of the active centers. Noteworthily, the reaction path of 2e^−^ and 4e^−^ changes only when the distance between the sites was less than 4 angstroms.

##### Improving the Accessibility of Active Sites

Generally, there is a certain proportional relationship between the metal loading and the number of active sites. However, not all metal sites act as active sites. The inaccessible metal sites do not participate in the catalytic reaction, while only those exposed on the catalyst surface can promote the catalytic reaction.^[^ ^182^ ^]^ Therefore, improving the accessibility of active sites is of great importance for maximizing effective site density and improving the exposure of the active site during catalytic reactions for ADMSEs.

Many strategies have been applied to improve the accessibility of active sites for ADMSEs.^[^ ^64^, ^129^, ^183^ ^]^ Especially for the gas‐phase migration strategy, the in situ pyrolysis strategy effectively prevents the aggregation and migration of metal atoms, facilitating the increase and accessibility of active sites for ADMSEs.^[^ ^15^, ^133^ ^]^ In addition, the gas‐phase migration strategy can also achieve large‐scale production of ADMSEs at industrial levels.^[^ ^64^, ^129^ ^]^ A NH_3_‐assisted gas‐migration strategy was developed for the direct conversion of bulk Cu_2_O to a monatomic Cu catalyst at the gram scale.^[^ ^129^ ^]^ During the high‐temperature pyrolysis in Ar atmosphere, Cu atoms from the surface of Cu foam were transformed into the volatile Cu(NH_3_)*_x_* species with NH_3_, and were confined by abundant defects in N‐doped carbon supports to form the isolated Cu sites. The isolated Cu atom loading and the calculated surface coverage were respectively 1.26% and 0.06 atoms nm^−2^. Gas‐phase migration strategy was also used to achieve the full utilization of active sites.^[^ ^15a^ ^]^ The isolated Fe site was formed for ORR by replacing the Zn atom with flowing iron chloride vapor under 750 °C.^[^ ^15a^ ^]^ Abundant Zn‐N_4_ sites were changed into Fe‐N_4_ sites, and anchored on the porous carbon substrates. The high‐resolution N 1s XPS spectrum from FeNC‐CVD‐750 could be ascribed to N‐O*_x_*, N^+^, N_gr_‐N, N‐H, M‐N*_x_*, and pyrinic N, and the presence of abundant Fe‐N_x_ moieties was proved by combining EELS spectroscopy and atomic‐resolution HAADF‐STEM characterizations. Notably, this strategy could achieve high active site density of 1.92 × 10^20^ sites g^−1^ and active site utilization of 100% for ORR catalysis. The Cu_2_O on the surface of the commercial CuO (I) powder was also sublimated to flowing steam, which was captured and reduced by N‐rich carbon support during the pyrolysis temperature of 1500 K, forming the isolated Cu ISAS/NC catalysts.^[^ ^64^ ^]^ The EPR result also proved the coordinatively unsaturated states of the isolated Cu species, and no Cu‐Cu bind could be observed in Cu ISAS/NC. The volatilization of Cu_2_O effectively avoided the corrosion of NH_3_ and improved accessibility of the Cu sits, which is benefited for large‐scale production and practical application. Similarly, gas‐migration strategy was used to achieve high loading and exposure of Mn‐N_x_ active sites for ORR.^[^ ^133a^ ^]^ Under the evaporation of MnCl_2_·4H_2_O at low temperature, the carbonization of ZIF‐8 was completed by a rapid heating process, accompanied by the formation of the atomic Mn‐N_x_ sites. This strategy effectively promoted the accessibility of Mn‐N*_x_* sites.

In addition, many other strategies are also applied to regulate active site density for ADMSEs.^[^ ^77^, ^182^, ^183^, ^184^ ^]^ A cooperative strategy of ordered template and steam etching was used to maximize the density of Fe‐N_4_ sites with interconnected ordered porous structures.^[^ ^184^ ^]^ Since the steam etching strategy could corrode the inactive amorphous carbon and selectively protect the active sites. The isolated Fe‐N_4_ sites were uniformly distributed on porous carbon, and the density of active sites (8.7 × 10^20^ sites g^−1^) was significantly increased by 4.6 times via steam etching. Moreover, the PEMFCs with the steam‐etching catalysts exhibited a higher peak power density of 0.78 W cm^−2^ and better durability compared with the Ar‐treated catalysts (0.63 W cm^−2^). Similarly, a densely exposed surface FeN_4_ (Sur‐FeN_4_‐HPC) on N‐doped porous carbon (NHPC) as a scaffold was achieved by anchoring of Fe ions and a subsequent pyrolysis strategy.^[^ ^183^ ^]^ The high surface area of NHPC with abundant surface Fe anchor sites ensured the formation of the densely accessible FeN_4_ active sites on Sur‐FeN_4_‐HPC (34.7 × 10^19^ sites g^−1^). Noteworthily, the current density of PEMFCs with Sur‐FeN_4_‐HPC was 24.2 mA cm^−2^ at 0.9 V_IR_ at 1.0 bar O_2_, and the maximum peak power density at 1.0 bar air reached 0.412 W cm^−2^. In addition, high‐density Fe sites effectively enhanced mass transport efficiency by exposing the less accessible Fe‐N_4_ sites.^[^ ^182a^ ^]^ The mesoporous Si coated ZIF‐8 was first preheated in Ar atmosphere of 650 °C, and then the Si layer was etched away, which made the positively charged Fe source more easily adsorbed. Finally, the precursor was pyrolyzed and carbonized in Ar/NH_3_ mixture to obtain Fe single atom catalyst. It was remarkable that the strategy significantly improved active site utilization of Fe sites, and the fuel cells performance for the first time met the non‐platinum catalyst activity target set by DOE in 2018. Chen et al.^[^ ^77^ ^]^ also constructed densely accessible Fe‐N*_x_* active sites on layered porous carbon for ORR. During the synthesis, the presence of Zn could facilitate the generation of microporous structure and avoid the formation of Fe_3_C@C species, achieving the formation of densely accessible Fe‐N_x_ active sites after subsequent leaching. In addition, ZnCl_2_ was also used to activate MOF for maximizing active site density, achieving the high‐performance ORR.^[^ ^182b^ ^]^ One‐step hydrothermal method was also applied to obtain meso/macro‐pores covalent organic polymer with highly accessible Fe‐N*_x_* sites, and the appropriate hierarchical pore structure effectively improved the mass transport efficiency of reaction intermediates and ensured the utilization of Fe sites for high‐efficiency PEMFCs.^[^ ^185^ ^]^

#### 6.1.2 Stability Issue for Atomically Dispersed Metal Site Electrocatalysts

Apart from the active site density, stability is also a crucial issue for the practical application of ADMSEs. Many efforts have been devoted to reducing the gap between the performance behaviors from before of life and end of life for ADMSEs, and addressing stability challenges is essential for the practical application of ADMSEs.^[^ ^186^ ^]^ At present, the stability evaluation for ADMSEs usually remains at the RDE level by comparing the activity loss before and after accelerated stress tests (AST), and the stability evaluation under real working conditions is insufficient for ADMSEs, hindering deeper understanding of the real degradation mechanism under the practical application. The main focus of the current work should be on the comprehensive and in‐depth understanding for the degradation mechanism, since a deeper insight for the degradation mechanism can provide a more valuable guideline to the rational design of efficient ADMSEs.^[^ ^186^, ^187^ ^]^ Generally, the proposed degradation mechanism for ADMSEs in electrochemical energy devices can be divided into the following parts: i) Carbon corrosion, ii) demetallation of active sites, and iii) the production of H_2_O_2_ and the attack of radicals.^[^ ^110^, ^186^, ^187^, ^188^ ^]^

##### Enhancing the Corrosion Resistance of Carbon Support

Carbon corrosion is a crucial issue for the stability of ADMSEs in practical application.^[^ ^187^, ^189^ ^]^ In general, carbon corrosion is not apparent during the RDE test, but it becomes a major challenge to deteriorate the active site in practical application. During the catalytic reaction, the by‐product of H_2_O_2_ is generally produced from the undesired two‐electron path, and carbon corrosion is generally induced by electro‐oxidation reaction under extreme operating conditions (e.g., high potential, high temperature, and high oxygen concentration) or the attack of reactive oxygen species (ROS) from H_2_O_2_ during the operation.^[^ ^187^, ^190^ ^]^ Carbon corrosion tends to reduce the active area and weaken the anchoring effect for the support, causing deactivation and decomposition of active sites.^[^ ^191^ ^]^ In particular, the ROS from the Fenton reactions of Fe centers greatly changes the surface chemical composition and electronic structure of support with oxygen‐containing carbon surface groups, causing the generation of carbon corrosion products and the degradation of the intrinsic stability of active sites.^[^ ^187^, ^189^ ^]^

The influence of different reaction atmospheres had been revealed. The presence of O significantly caused more severe performance degradation after AST than other reaction environments, especially the activity degradation for Fe‐N sites after the O_2_‐load‐cycling protocol (mass activity loss of 66%) reached more than four times than operation after the Ar‐load‐cycling AST (mass activity loss of 16%).^[^ ^191a^ ^]^ Unlike the conventional activity degradation at high potential (0.6–1.0 V vs RHE), much unexpected carbon corrosion was clearly observed at low potential (0.5–0.9 V vs RHE or even 0.3–0.7 V vs RHE) at O_2_ atmosphere due to the attack of ROS from the Fenton reactions of Fe sites, indicating that higher electrochemical potentials (up to 1.0 V vs RHE) didn't cause more significant performance degradation during O_2_‐load‐cycling protocol. Many related characterizations demonstrated the presence of Fe oxide nanoparticles after O_2_‐load‐cycling protocol, and the initial Fe sites from the leaching of Fe‐N_x_ tended to aggregate into larger clusters and particles due to carbon corrosion. In general, H_2_O_2_ or ROS appears in situ during ORR not only leads to slight surface oxidation, but also causes irreversible carbon corrosion. Noteworthily, the formation of volatile products CO and CO_2_ during ORR also resulted in the formation of new pore structures and increase of carbon area, destroying the structure of active sites. The activity loss of Fe‐N*_x_* sites mainly involving carbon corrosion and destruction of active sites was further demonstrated.^[^ ^192^ ^]^ Compared with potential cycles of 0.6–1 V, more significant performance degradation was found under a constant potential of 0.85 V in O_2_ saturated acid solution. Notably, part of ORR activity could be recovered in the 0–1 V range during durability tests, and this reversible decay might result from the surface functionalization of the carbon support. However, the degradation caused by demetallation was not recovered throughout the durability test. After 100 h endurance test, the activity was only 30% of the original performance. The HAADF‐STEM also demonstrated the formation of nanoparticles after the durability test, indicating the isolated Fe atoms tended to aggregate into Fe clusters or nanoparticles with the decay of the catalyst, which might be one of the reasons for the catalyst inactivation.

Similarly, some research has been undertaken about the formation of the irreversible carbon corrosion caused by ROS, which gives rise to the deactivation of Fe‐N*_x_* sites.^[^ ^187^, ^193^ ^]^ A deeper insight was devoted to the degradation of Fe sites at different temperature ranges of 20–80 °C in acidic medium.^[^ ^187a^ ^]^ At the condition of high temperature (e.g., 80 °C), the activity degradation of Fe sing atom catalysts was closely related to carbon corrosion and parallel non‐preferential dissolution of Fe‐N*_x_* sites, and decreased the charge and mass transport efficiency, causing the destruction of the electrode. Notably, some Fe particles and FeN_x_ sites were still present at high voltage, and corresponding signals could be identified by Mössbauer spectroscopy, suggesting that the high‐voltage cycling did not preferentially destroy Fe particles or Fe‐N*_x_* sites. To inhibit the carbon corrosion, more efforts have been made to improve the graphitization degree for the carbon support, and the strategy can effectively extend the durability of ADMSEs under real operation conditions.^[^ ^194^ ^]^

##### Mitigating the Demetallation of Active Sites

Demetallation of active sites generally leads to the decrease of site density directly, causing the activity degradation of ADMSEs. In the ORR process, some monodispersed metal sites mainly come from the demetallation of metal particles.^[^ ^189^, ^195^ ^]^ Although the catalytic activity is not affected in the short term, a large amount of Fe ions will further catalyze the Fenton reaction to generate the undesired ROS, which will further attack the membrane and catalyst, leading to the decline of catalytic efficiency and hindering the practical application of ADMSEs.^[^ ^196^ ^]^

The demetallation is not an independent behavior, but the result of carbon corrosion.^[^ ^189^ ^]^ At low potential, the demetallation behavior mainly came from ferrous iron species, and the solvated Fe^2+^ cation could be also found at low pH atmosphere (*E* < 0.77 V_SHE_). Especially for low potential, the leaching of Fe mainly came from the surface‐oxidized Fe particles. However, these leaching behaviors did not decrease the ORR performance, indicating the inactivity or low activity of Fe crystalline structures for ORR in acidic environment. Noteworthily, the CO_2_ and CO evolution behaviors could be observed at high potential, implying the essential role of carbon corrosion in the degradation mechanism. The removal of inactive Fe particles is of great importance for improving the stability of catalysts.^[^ ^195^ ^]^ However, due to the formation of insoluble Fe species under high potential (e.g., 0.9 V_RHE_), Fe particles were difficult to be completely removed under acidic conditions, and additional Fe particles could be dissolved and penetrate into the membrane under operating conditions, causing performance degradation of PEMFCs. Several methods have also been proposed to inhibit the demetallation of Fe sites for improving the intrinsic stability, including the synthesis of isolated Fe site catalysts without Fe particles and postsynthesis removal of exposed Fe particle catalysts. The operando spectroscopic analysis proved that the demetallation behaviors of Fe sites were obviously weakened for the above synthesis strategies. Noteworthily, similar behavior of activity degradation was also found during the short‐term durability test, indicating the less responsibility of Fe particles for activity loss. In like manner, Chenitz et al.^[^ ^188a^ ^]^ also proved that the demetallation behavior of Fe‐N_4_ sites in the micropores was the main cause for the initial activity loss of catalysts in fuel cells. Water quickly flowed in fuel cells with a width of less than 0.7 nm because they did not interact with the hydrophobic walls of the micropores, and the Fe‐N_4_ sites were demetallized in the water flow of the microporous.

In addition, the adsorption and binding of O_2_ on active sites also decrease the interaction of metal sites and coordinated atoms, causing more severe demetallation than other operations under an inert atmosphere, including electrochemical potential and electrolyte.^[^ ^197^ ^]^ DFT was also applicated to reveal the acid stability and demetallation of Fe‐N_4_ for ORR and the intrinsic relationship among site structure, the environment conditions and reaction intermediates.^[^ ^198^ ^]^ In particular, the stability of Fe‐N_4_ is closely related to PH and electric potential, and there was a certain linear relationship among them. Generally, the selected Fe‐based structures were not acid stable in the absence of ligands or binding ORR intermediates, suggesting that the demetallation was the main reason for the stability degradation for Fe‐N_4_ sites. In addition, ROS also attacked the protective ligands, which increased the exposure of Fe‐N_4_ sites, causing the dissolution of center sites at low PH environment.

##### Inhibiting the Production of H_2_O_2_ and Weakening the Attack of Reactive Oxygen Species

H_2_O_2_ as a byproduct of ORR is generally produced from the undesired 2e^−^‐pathway Fenton reaction, and the ROS from the H_2_O_2_ generally attacks the M‐N_x_ sites of ADMSEs and membrane of electrochemical energy devices, causing the performance degradation of ADMSEs.^[^ ^188^, ^199^ ^]^ The attack of hydroxyl free radicals (•OH) from H_2_O_2_ for Fe sites can especially cause a significant loss of ORR activity.^[^ ^200^ ^]^ From the DFT, the desorption of •OH produced from the Fenton reaction was determined as the rate‐limiting step for the elementary reactions, which was mainly due to the strong binding strength between the •OH and Fe sites. Moreover, as the number of the adsorbed •OH increases, the Fe sites were more easily destroyed. Noteworthily, three •OH were enough to turn the Fe sites into Fe ions in acidic medium, and the leached Fe ions further penetrated into the membrane and catalytic layer, causing the increase of membrane resistance and catalyst layer proton conduction resistance during the operation. In addition, the work also proved the incorrectness of the previous conclusion that the quick performance loss of Fe sites was mainly dominated by water flooding within the catalyst layer. Inhibiting the production of H_2_O_2_ and weakening the attack of ROS still remains the focus of current research, and how to address the stability challenge for ADMSEs has been thrust into the limelight, including enhancing the selectivity of 4e^−^ pathway and focusing on non‐iron site catalysts during ORR.^[^ ^113^, ^201^ ^]^

Apart from the attack of ROS, especially for fuel cells, the protonation behavior of active sites is also regarded as the potential cause for the performance degradation of ADMSEs.^[^ ^202^ ^]^ At present, the proposed protonation mechanism mainly involves N‐protonation and anion‐binding effects, and the protonation of N and binding of anion in acidic medium generally cause activity degradation.^[^ ^202^ ^]^ The protonation of the surface N‐groups could occur at PH1, and followed by binding to anions, this behavior generally caused significant activity degradation.^[^ ^202^ ^]^ However, the anions adsorbed on the surface during the acid wash could be removed by chemical and thermal treatments to restore the ORR activity, and the recovery of ORR activity was attributed to the acid‐resistant FeN_4_ sites. Noteworthily, the turnover frequency of FeN_4_ sites for ORR was dominated by chemical state of basic N‐groups. The protonated N‐groups but not yet anion bound generally led to a high turnover frequency, while the turnover frequency of ORR was relatively low when the protonated basic N‐groups were neutralized by anions.

### 6.2 Applications of Atomically Dispersed Metal Site Electrocatalysts for Electrochemical Energy Devices

The practical application of ADMSEs is of vital importance for the development of a sustainable society. As the promising electrochemical energy devices, fuel cells and Zn–air batteries are regarded as the next‐generation potential energy conversion and storage technologies. At present, ADMSEs have showed the efficient ORR performance in both acidic and basic mediums, and are widely developed and applied in fuel cells and Zn–air batteries.^[^ ^24^, ^41^, ^85^, ^146^, ^203^ ^]^ In the following part, we will focus on the practical application of ADMSEs in fuel cells and Zn–air batteries.

#### 6.2.1 Atomically Dispersed Metal Site Electrocatalysts for Fuel Cells

At present, Pt group metals show the outstanding ORR performance, and are extensively used as cathode catalysts for fuel cells. However, the high cost and insufficient durability of Pt group metal catalysts greatly hamper their practical application. ADMSEs are considered as the promising substitutes for Pt group metal catalysts, tremendous efforts have been invested in their development, showing great application prospects in fuel cells.^[^ ^24^, ^204^ ^]^

**Figure 17:**
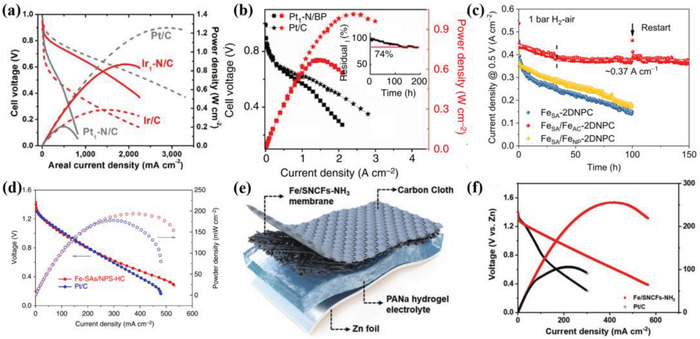
a) Polarization curves and power density curves of H_2_‐O_2_ PEMFCs with different catalysts. Reproduced with permission.^[^ ^41b^ ^]^ Copyright 2020, Wiley‐VCH. b) The polarization curves and power density curves of the acidic fuel cells in H_2_/O_2_ with different samples as cathode catalysts. Reproduced with permission.^[^ ^204a^ ^]^ Copyright 2017, Springer Nature. c) The changes of current density (0.5 V) with time. Reproduced with permission.^[^ ^205^ ^]^ Copyright 2022, Springer Nature. d) The discharging polarization curves and power density curves of Zn–air batteries with Fe‐SAs/NPS‐HC and commercial Pt/C as cathode catalysts. Reproduced with permission.^[^ ^146^ ^]^ Copyright 2018, Springer Nature. e) The simplified schematic of the Flexible Zn–air batteries with a (PANa)‐KOH‐Zn(CH_3_COO)_2_ hydrogel as the electrolyte. Copyright 2021, Wiley‐VCH. f) The discharge polarization curves and corresponding power density of the Zn–air batteries with Fe/SNCFs‐NH_3_ as air cathode catalysts. Reproduced with permission.^[^ ^85^ ^]^ Copyright 2021, Wiley‐VCH.

Recent years have seen the wide application of ADMSEs in fuel cells.^[^ ^41^, ^62^, ^203^ ^]^ A series of precious metal single‐atom catalysts (Ir, Rh, Pt and Pd) had been developed through a sequential coordination method.^[^ ^41b^ ^]^ The sequential synthesis strategies could efficiently modulate the matching degree between the OH^*^ adsorption and the electronegativity of coordinating anions. In the acidic half‐cell test, Ir and Rh single‐atom catalysts exhibited more outstanding ORR performance than commercial Ir/C and Rh/C. It was notable that the PEMFCs from Ir single‐atom catalysts could achieve a higher power density of 870 mW cm^−2^ and mass specific power density of 6.44 W mg^−1^_Ir_ with H_2_ and O_2_ feeds, which was higher than that of Ir/C (387 mW cm^−2^ and 1.15 W mg^−1^_Ir_) (**Figure** **17**a). In addition, the H_2_‐air PEMFCs also showed a high peak power density of 0.380 W cm^−2^. Ir single‐atom catalysts‐based PEMFCs also exhibited high current retention of 50% during 50 h test at 0.5 V, embodying the great application potential for Ir single‐atom catalysts. Similarly, ultralow Pt loading also achieved high cell performance polymer electrolyte membrane fuel cells.^[^ ^204a^ ^]^ Unlike the bulk Pt, oxygen molecules tended to be adsorbed on Pt metal sites in a side‐on configuration. Noteworthily, the Pt adsorption on Pyridinic N_1_‐doped graphene efficiently promoted the decomposition of H_2_O_2_ into OH groups, improving catalytic performance in fuel cells. Pt single atom catalysts showed comparable cell voltage and power density to bulk Pt/C. Under the durability test of constant 0.5 V potential, the acidic fuel cell with Pt_1_‐N/BP as cathodic catalysts (0.01 mg_Pt_ cm^−2^) could remain the current of 90% and 74% at 70 and 80 °C after 200 h, respectively, implying better durability than other catalysts (Figure 17b). In addition to noble catalysts, non‐noble catalysts are widely applied to fuel cells.^[^ ^52^, ^62^, ^203^, ^204^ ^]^ Atomically dispersed Fe sites anchored on hierarchical ordered porous carbon (FeN_4_/HOPC) could observably promote catalytic efficiency.^[^ ^203^ ^]^ The optimal FeN_4_/HOPC catalysts exhibited comparable half‐cell performance with an *E*_1/2_ of 0.80 V in acidic electrolyte to commercial Pt/C (0.82 V). For PEMFCs application, FeN_4_/HOPC‐c‐1000 exhibited a higher power density of 0.42 W cm^−2^ at 0.57 V and open circuit voltage of 0.98 V than FeN_4_/C, and respectively achieved current densities of 0.75 and 0.69 A cm^−2^ at 0.6 V for 1 bar H_2_‐O_2_ and H_2_‐air than that of FeN_4_/C, indicating outstanding output performance for FeN_4_/HOPC‐c‐1000. An N‐anchored Fe nanocluster with a satellite Fe‐N_4_ site (FeSA/FeAC‐2DNPC) was successfully synthesized on 2D porous carbon as a highly efficient ORR electrocatalyst for acid fuel cells.^[^ ^205^ ^]^ For the special catalyst structure, Fe clusters effectively reduced the ORR energy barrier by introducing an OH ligand, thereby increasing the activity of the satellite Fe‐N_4_ sites. Noteworthily, the Fe‐N bond amplitude of the satellite Fe‐N_4_ was shortened by the interaction of Fe clusters and isolated Fe sites in the PEMFC, and the demetallation of Fe‐N_4_ was reduced by 60%. FeSA/FEAC‐2DNPC exhibited more outstanding durability than that of traditional Fe‐N‐C monatomic catalysts (Figure 17c). In addition, dual metal sites can also achieve high performance in fuel cells.^[^ ^52^, ^148^, ^153^, ^204^ ^]^ The Fe‐Co dual sites embedded on N‐doped porous carbon ((Fe, Co)/N‐C) showed excellent catalytic performance in acid fuel cells.^[^ ^204b^ ^]^ The catalyst presented comparable initial potential (*E*_onset_, 1.06 V vs 1.03 V) and *E*_1/2_ (0.863 V vs 0.858 V) to that of commercial Pt/C in half cells. Fuel cell tests exhibited that (Fe, Co)/N‐C catalysts had superior catalytic performance to most Pt‐free catalysts reported for H_2_/O_2_ and H_2_/air. Besides, (Fe, Co)/N‐C catalysts also showed more stable activity after the durability test of 50 000 cycles of electrode measurements and 100 h in H_2_/air condition. The enhanced catalytic performance could be ascribed to the O‐O activation on the double sites. For another example, atomically dispersed Co/Zn atomic pairs efficiently lowered the dissociative barrier of the intermediate during catalytic reaction, PEMFC with Co/Zn dual‐sites as cathodic catalysts could achieve a high power density of 0.603 W cm^−2^ at H_2_‐O_2_, and exhibited long‐term stability after 150 h at current density of 400 mA cm^−2^.^[^ ^52^ ^]^ Since the production of hydrogen peroxide usually led to the generation of free radicals, causing the rapid decay of the membrane electrode from fuel cells, it was the importance to inhibit the occurrence of the Fenton reaction for the development of the advanced fuel cells. The Co nanoparticle and CoN_4_ composite sites with deformed CoN_4_ were constructed for the development of advanced PEMFCs.^[^ ^62^ ^]^ Theoretical study showed that both Co nanoparticle and CoN_4_ composite sites and deformed CoN_4_ could promote the activation of O_2_. Noteworthily, the composite sites effectively inhibited the formation of H_2_O_2_, enhanced the adsorption of free radical oxygen and greatly reduced carbon layer erosion. For the fuel cell test, the designed Co nanoparticle and CoN_4_ composite sites based PEMFCs had an excellent power density of 1.207 W cm^−2^ at 0.36 V under 1 bar H_2_/O_2_ and exhibited higher current densities retention at cell voltage of 0.6 V for 100 h, indicating better durability for the composite sites. CoN_4_ sites confined into 3D porous carbon also achieved comparable catalytic performance to Fe‐based catalysts, and inhibited the production of hydrogen peroxide, exhibiting better cell performance in PEMFCs.^[^ ^206^ ^]^

#### 6.2.2 Atomically Dispersed Metal Site Electrocatalysts for Zn–Air Batteries

Zn–air batteries have attracted great interests of researchers because of their high theoretical energy density, and ADMSEs have been proved to have an extremely high potential for Zn–air batteries in practical application.^[^ ^24^, ^51^, ^55^, ^85^ ^]^ Many indicators are used to evaluate the cell performance of Zn–air batteries in practical applications, including open circuit voltage, current density, power density, specific capacity, and energy density, etc.^[^ ^55^, ^146^ ^]^

Chen et al.^[^ ^146^ ^]^ developed a single Fe site catalyst on N, P, and S co‐doped hollow carbon (Fe‐SAs/NPS‐HC) by means of the Kirkendall effect for Zn–air batteries. The synthesis strategy efficiently improved catalytic performance for Zn–air batteries, and the enhanced ORR kinetics and activity could be ascribed to electronic interaction between active metal sites and long‐range S and P. For application, SAs/NPS‐HC‐based Zn–air batteries exhibited more outstanding open circuit voltage of 1.45 V than commercial Pt/C. In addition, the maximum power density for SAs/NPS‐HC‐based Zn–air batteries reached 195.0 mW cm^−2^ at current density of 375 mA cm^−2^ (Figure 17d). Pt and Fe dual atom catalysts (PtFeNC) by encapsulating Pt sites into Fe‐doped ZIF were also developed for Zn–air batteries.^[^ ^55^ ^]^ The synergistic effect between Pt and Fe sites efficiently improved the catalytic activity, and inhibited the occurrence of side reactions. The PtFeNC‐based Zn–air batteries exhibited a considerable open circuit voltage of 1.492 V, which was close to the theoretical value of 1.667 V. In addition, the Zn–air batteries also showed a specific capacity of 807 mAh g^−1^ at a discharge current density of 10 mA cm^−2^. A novel bimetallic monatomic catalyst (FeMn‐DSAC) on 2D ultra‐thin N‐doped carbon nanosheets with adjacent Fe‐N_4_ and Mn‐N_4_ sites was reported for flexible low‐temperature Zn–air batteries.^[^ ^90^ ^]^ The synergistic effect of Fe‐Mn double sites effectively promoted the decomposition of OOH. The peak power density of the assembled Zn–air batteries with FeMn‐DSAC as the cathode catalysts reached 30 mW cm^−2^ at −40 °C. Other dual site catalysts were also proved to be effective for Zn–air batteries.^[^ ^148^, ^149^, ^152^ ^]^ Furthermore, ADMSEs have also been demonstrated to has great potential for flexible Zn–air batteries.^[^ ^85^, ^149^, ^207^ ^]^ A highly exposed atomic Fe‐N_4_/C active site (Fe/SNCFS‐NH_3_) anchored on a free‐standing flexible carbon fiber membrane was developed for flexible Zn–air batteries, and the rapid penetration of reactants and high density of Fe‐N_4_/C sites were achieved (Figure 17e).^[^ ^85^ ^]^ The S and N co‐doped carbon matrix not only firmly immobilized the Fe‐N_4_/C site on the entangled CFs, but also regulated the electronic structure of the Fe‐N_4_/C site through long‐range interaction, ensuring the stability and catalytic activity of Fe‐N_4_/C. The liquid Zn–air batteries with Fe/SnCFS‐NH_3_ catalysts as air cathode exhibited a higher open‐circuit voltage of 1.38 V and a peak power density of up to 255.84 mW cm^−2^ than that of commercial Pt/C (106.08 mW cm^−2^) (Figure 17f). Almost no decay in cell performance could be observed under continuous galvanostatic discharging‐charging test of 1 mA cm^−2^, and the assembled Zn–air batteries also showed good cyclic stability under various bending states. Similarly, A pH‐universal Fe single‐atom catalyst derived from double‐layer MOF (Fe_1_/d‐CN) also exhibited excellent catalytic performance for flexible Zn–air batteries.^[^ ^207^ ^]^ The open circuit voltage and peak power density of Zn–air batteries was up to 1.50 V and 78.0 mW cm^−2^, respectively. Negligible degradation could be observed after durability test of 30 000 cycles at the full pH range. The enhanced cell performance was mainly ascribed to the regulation of electronic structure and the good electron/proton transport ability from rich defects and porous structure for Fe_1_/d‐CN catalysts.

## 7 Summary and Outlook

**Figure 18:**
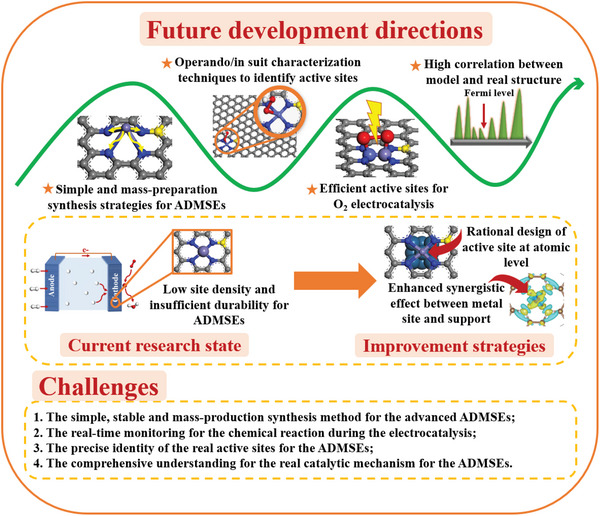
The current research state, improvement strategies, great challenges, and future development direction in the rational design of the state‐of‐art ADMSEs for the renewable energy conversion and storage techniques.

As one of the promising next‐generation electrocatalysts for future energy conversion and storage techniques, ADMSEs not only act as the indispensable bridge between the homogeneous and heterogeneous catalysis, but also provide the well‐defined model active sites for a deeper insight into the catalytic mechanism. The electronic properties are closely related to the catalytic performance, and dominate the whole catalytic reaction. Optimizing the electronic structure of ADMSEs effectively modulates the electronic filling state of d orbital, facilitates the p‐d orbital hybridization effect between active metal sites and reactants or intermediate species and reduces the energy barrier of RDS for ADMSEs. Particularly, the strong electron coupling between the central metal site and surrounding coordination environment greatly triggers the charge density redistribution, regulates the valence state and optimizes the binding strength between active sites and reaction intermediate species. Effective design strategies for atomic structure can provide a valuable guideline for the rational design of ADMSEs. In this review, we summarized the crucial advances for the advanced ADMSEs in fuel cells and Zn–air batteries, with a discussion on the catalytic mechanism, characterization techniques and synthesis method for ADMSEs. A special emphasis is placed on the critical strategies for the rational design of ADMSEs. The ultimate goal is to provide a comprehensive and profound insight for the understanding of the ORR mechanism and the rational design of the advanced ADMSEs. Despite many progresses have been obtained, some pivotal issues have not been resolved for the development of the advanced ADMSEs. Here, the current research states, improvement strategies, the great challenges, and future development directions for ADMSEs in fuel cells and Zn–air batteries are summarized below (**Figure** **18**).

### 7.1 Revealing the Catalytic Mechanism for Atomically Dispersed Metal Site Electrocatalysts

A comprehensive understanding of ORR catalytic mechanism is highly desired for the rational design of ADMSEs. At present, many theoretical calculation simulations along with the advanced techniques have been applied to reveal the structure–activity relationship from the catalytic reaction thermodynamics and kinetics. Although more emphasis has been placed on ADMSEs, the optimal electronic structure still remains unclear, and more efforts should be encouraged to explore the favorable active site for ORR. Most of the theoretical calculation results are mainly based on pre‐structure instead of the actual structure for ADMSEs, which can't reflect the real catalytic reaction processes, especially when little consideration is given to the deformation of support substrate for the theoretical calculation model, causing the lack of the accurate understanding for catalytic mechanism.^[^ ^3^, ^34^, ^62^ ^]^ Moreover, the theoretical calculations are generally conducted under the simplified conditions, including vacuum and absolute zero, so the relevant simulation conditions should be further optimized and enhanced for obtaining real catalytic mechanisms. The machine learning is also proposed to predict and optimize the most potential ORR catalysts before the experiment, and more efforts are desired to develop the appropriate descriptors for precisely revealing and reflecting the intrinsic catalytic mechanism. In addition, to reduce the complexity of the catalytic mechanisms, the rational design of model active sites with a well‐defined local coordination environment is highly necessary.

### 7.2 Identifying the Real Atomic Structure for Atomically Dispersed Metal Site Electrocatalysts

A deeper insight for the atomic structure is regarded as one of the greatest challenges for revealing the structure–activity relationship of ADMSEs. At present, some crucial issues regarding characterization techniques such as the precise identity of the atomic structure (e.g., STEM and XAS) haven't been addressed. STEM is usually applied to directly present the spatial distribution of the isolated metal sites, while there are some limitations to show the spatial distribution of proximal light atoms and achieve the chemical identity of the heteroatoms around the central metal atoms with low Z contrast, such as the C, N, P, and S from the surrounding coordination shells. The local coordination environment and electronic structure of ADMSEs are generally explored by the XAS, including the valence state of central metal atom, bond length, and coordination atom type and number. However, many information about the surrounding coordinated environment is not directly obtained, but is determined by the results of experimental and theoretical calculations. Moreover, due to the complexity of adsorption and desorption behaviors during ORR, the characterization technique can't precisely identify the real active sites due to its real‐time change under the corrosive working environment, and the probe for the intermediates adsorbed on the active sites still remains challenging. Direct identification of the active sites is extremely crucial for the rational design of the advanced ADMSEs, especially the in situ/operando XAS can provide a real‐time monitoring of the catalytic reaction for ADMSEs. Currently, the fact that despite many works have been conducted about the in situ/operando characterization techniques, the details of the real active sites, reaction pathways, reaction intermediates and catalytic mechanism have not been accurately determined yet. To precisely reveal the atomic structure and its structure evolution caused by the chemical interaction with reaction intermediates for ADMSEs, various characterization techniques should be combined to verify the experimental results.

### 7.3 Developing Simple, Stable, and Mass‐Production Synthesis Strategy for Atomically Dispersed Metal Site Electrocatalysts

In terms of synthesis, the efficient synthesis method is generally considered as the prerequisite for the rational design of the advanced ADMSEs, and developing a simple, stable and batch‐production method of controllably preparing ADMSEs still remains a huge challenge. At present, the practical application of ADMSEs is still faced with problems such as low active site density and insufficient durability, and many synthetic strategies are usually used to obtain ADMSEs by reducing the targeted metal loading, which causes the decrease of the active metal sites. Moreover, these synthesis strategies generally include many complicated and tedious procedures, and are difficult to achieve the stable and large‐scale preparation for the practical application of ADMSEs. Additionally, many of the key issues impeding commercialization still remain, including the agglomeration of metal atoms into clusters or nanoparticles due to the high free energy during the synthesis process and the deactivation of ADMSEs due to the agglomeration during the catalytic reaction. Stability is also crucial for the practical application of ADMSEs under the harsh corrosive environment. Especially for the acidic solution, these corrosive environments tend to deteriorate the catalytic performance of ADMSEs, causing poor stability and sluggish reaction kinetics. More efforts should be encouraged to focus on the synergistic interaction between the metal sites and the support substrates for obtaining efficient ADMSEs.

### 7.4 Designing Rational Atomic Structure for Atomically Dispersed Metal Site Electrocatalysts

Generally, active sites dominate the catalytic performance of ADMSEs, and thus an imperative and arduous task is to rationally design active sites at the atom level. In fact, many effective strategies have been proposed for the rational design of ADMSEs, mainly involving the regulation of central metal atoms, coordinated atoms and the coupling of mononuclear and polynuclear metal species. However, it is difficult to figure out the real catalytic mechanism for the lack of the model catalyst, and some confusions still remain unresolved about these strategies: 1) The role of the strategy. Due to the lack of the well‐defined model active site configurations, it is difficult for some works to precisely reflect the real role of different strategies on the enhanced ORR performance, which hinders the development of effective regulation strategies. 2) The simultaneous development of various strategies. At present, little work involves the simultaneous use of various strategies for ADMSEs in the absence of controllable synthesis techniques. Moreover, the effects from the simultaneous use of these strategies still remain unclear due to the complexity of the synergistic effect between the central metal site and coordination environment, and it is difficult to understand that the strategies promote or worsen the whole ORR performance of ADMSEs. 3) Effective coordination environment range. Many studies have demonstrated the positive effect of coordination environment on catalytic performance, while the regulation strategies mainly involve the first and second coordination shells, and the effective coordination environment range for regulating ORR performance has not been clarified.^[^ ^2^, ^16^, ^49^ ^]^ It is essential to understand the effect of the long‐distance coordination environment on the catalytic performance such as the third and fourth coordination shell. Moreover, since the ORR performance of ADMSE is still not comparable to the noble metal based electrocatalysts, more efforts should be devoted to exploring the effective regulation strategies for the development of the advanced ADMSEs. Especially the effective coupling between mononuclear and polynuclear metal species is also a potential way for the advanced electrocatalysts.

All in all, ADMSEs are regarded as the potential ORR electrocatalysts for the future energy conversion and storage techniques. Currently, despite the challenges still exist around ADMSEs, their huge application potentials make us believe that the co‐development of other advanced technologies such as characterization techniques, synthesis methods, and theoretical calculation methods will bring the catalytic performance of ADMSEs to a new level for meeting the needs of practical applications and promoting the development of a clean energy society.

## Conflict of Interest

The authors declare no conflict of interest.
